# Causal relationship between serum metabolites and chronic myeloid leukemia: A bidirectional Mendelian randomization study

**DOI:** 10.1097/MD.0000000000045217

**Published:** 2025-10-10

**Authors:** Haohan Ye, Jun Tang, Yuanheng Liu, Xiaoli Li

**Affiliations:** a School of Basic Medical Sciences, Chongqing Medical University, Chongqing, China; b Center for Experimental Teaching Management, Chongqing Medical University, Chongqing, China; c Laboratory of Developmental Biology, Department of Cell Biology and Genetics, School of Basic Medical Sciences, Chongqing Medical University, Chongqing, China.

**Keywords:** causal relationship, chronic myeloid leukemia, Mendelian randomization, metabolic pathways, serum metabolites

## Abstract

Previous studies have found a link between serum metabolite levels and chronic myeloid leukemia (CML), yet their exact causal relationship remains unexplored. Using genome-wide association datasets, we conducted bidirectional Mendelian randomization (MR) analyses to explore the potential causal relationship between 486 serum metabolites and CML. We conducted sensitivity analysis to assess the presence of heterogeneity and pleiotropy. Our Mendelian randomization analysis identified 20 metabolites exerting significant causal effects on CML, including 19 known and 1 unidentified metabolite. Among the 19 identified metabolites, 10 metabolites exhibit a risk effect in CML, whereas 9 manifested a protective effect. Notably, the amino acid metabolite 4-methyl-2-oxopentanoate displayed the strongest positive causal relationship with CML. The CML-associated metabolites were predominantly enriched in the following metabolic pathways: caffeine metabolism, glycerolipid metabolism, glycerophospholipid metabolism, and valine, leucine, and isoleucine biosynthesis. These findings advance our understanding of metabolic interactions in CML, providing critical insights for diagnosis and guiding strategies for prevention and treatment.

## 1. Introduction

Chronic myeloid leukemia (CML) is classified within the spectrum of myeloproliferative neoplasms, a group of disorders characterized by dysregulated proliferation of myeloid cells across distinct stages of maturation. Clinically, patients with CML typically manifest in 3 distinct disease phases: chronic phase (CP), accelerated phase (AP), and blast phase (also termed blast crisis, BC). Initially, the majority of patients present in CP; however, without therapeutic intervention, disease progression typically ensues through AP to BC. A minority of patients may bypass the AP and transition directly to the BC. The clinical manifestations of CML are generally nonspecific, encompassing fever, fatigue, and weight loss. Notably, disease progression to the BC is often accompanied by exacerbation of symptoms, with the emergence of additional features such as bleeding and bone pain.^[1]^ According to global cancer statistics, the incidence rate of CML is ~0.8 to 2.1 per 100,000 population.^[2]^ Over the past 2 decades, the prognosis of patients with CML in the CP has witnessed remarkable improvement, primarily attributed to the incorporation of small-molecule tyrosine kinase inhibitors (TKIs) into clinical CML management. Nonetheless, monotherapy with TKIs may fail to achieve a complete eradication of Philadelphia chromosome-positive (Ph+) leukemic cells, and therapeutic responses in the AP or blast phase of the disease often remain transient,^[3]^ ultimately necessitating lifelong TKI therapy.^[4]^ Furthermore, a subset of CML patients receiving TKI treatment still develops resistance, which exhibits heterogeneous and patient-specific characteristics. The precise mechanisms underlying this resistance remain incompletely understood, representing a major challenge in CML management. Therefore, elucidating the molecular mechanisms driving CML pathogenesis and developing novel therapeutic strategies are critical for improving clinical outcomes.

Although the precise etiology of CML remains incompletely understood, its pathogenesis is intricately linked to genetic predispositions, immune dysfunction, and environmental elements. Emerging investigations further underscore the complex and intimate interplay between serum metabolites and CML.^[5,6]^ An investigation employed liquid chromatography-mass spectrometry metabolite profiling coupled with multivariate statistical methods to analyze plasma and white blood cell samples from patients newly diagnosed with CML, patients receiving hydroxyurea and TKIs (imatinib, dasatinib, nilotinib) treatment, and healthy controls. Key findings revealed that primary metabolic alterations across these groups were predominantly localized to glycolysis, citric acid cycle, and amino acid metabolism.^[7]^ A study has revealed that serum kynurenine levels are significantly elevated in patients with CML, and a robust positive correlation has been observed between KYN and uric acid levels.^[8]^ A study utilized chromatography-mass spectrometry-based metabonomic approach to analyze plasma samples from CML patients and control groups, identifying 9 differential metabolites between CML patients and healthy controls. These metabolites included isoleucine, glycerol, lactic acid, glycine, D-sorbitol, D-galactose, D-glucose, myristic acid, and inositol. Notably, glycerol and myristic acid exhibited the most significant association with TKI treatment efficacy.^[9]^ Accumulated evidence supports a strong association between serum metabolites and CML. Nevertheless, the majority of supporting data originate from preclinical investigations, which are inherently prone to confounding variables and reverse causality. Thus, the causal relationship between serum metabolites and CML warrants systematic exploration.

Mendelian randomization (MR) leverages genetic variants as instrumental variables (IVs) to elucidate causal associations between exposures and diseases within observational research frameworks.^[10]^ MR can generate unbiased estimates of genotype determination at conception and is generally impervious to confounding factors and reverse causation.^[11]^ MR has been extensively applied in oncological research, providing critical insights into the etiological mechanisms underlying various malignancies. Zhuang et al applied 2-sample MR to identify serum metabolites and immune cells as key causal drivers of lymphoma, while Chen et al demonstrated the role of gut microbiota-derived metabolites in leukemia via MR, highlighting the method’s value in dissecting metabolic mechanisms across blood cancers.^[12,13]^ In this study, we utilized 2-sample MR to investigate the causal association between serum metabolites and CML. This investigation aims to elucidate potential causal associations between serum metabolites and CML, with the objective of identifying novel therapeutic targets to advance disease prevention and targeted therapeutic strategies.

## 2. Materials and methods

### 2.1. Study design

**Figure 1.**
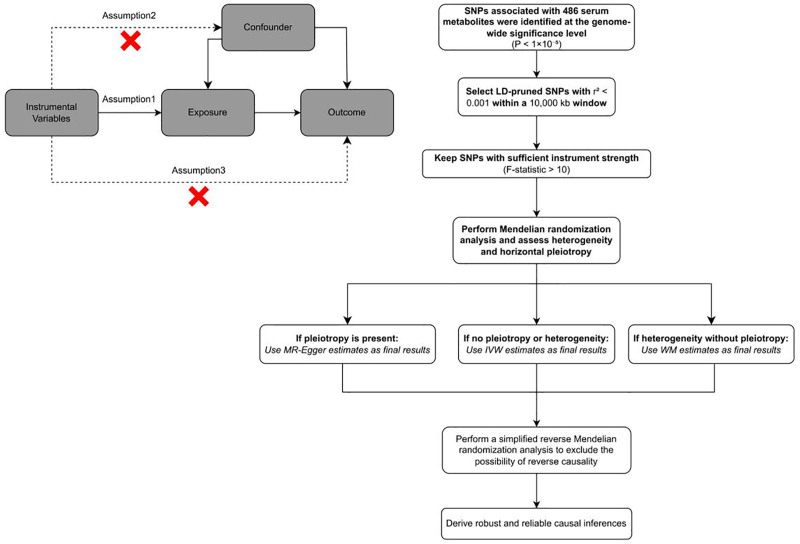
The overall designation of this study.

The study workflow is presented in Figure 1. Using a bidirectional MR approach, we examined the causal associations between 486 serum metabolites and CML. MR employs genetic variants as instrumental proxies for risk factors and evaluates the robustness of its findings through sensitivity analyses. Valid IVs in MR must adhere to 3 core assumptions: the genetic instruments are directly associated with the exposure; the genetic instruments are uncorrelated with any confounding factors between the exposure and outcomes; the genetic instruments influences outcomes solely through the exposure. Since our data were derived from public repositories, ethical approval was exempted. To ensure the robustness of our findings, comprehensive sensitivity analyses were conducted. Finally, we performed a metabolic pathway enrichment analysis on the serum metabolites identified in this study. All analyses were conducted using R software (version 4.3.3) with the TwoSampleMR package (version 0.5.11).

### 2.2. Data source

The genome-wide association study (GWAS) data for serum metabolites were derived from the study by Shin et al,^[14]^ which remains the most comprehensive analysis of serum metabolites documented to date. This GWAS identified 2.1 million single nucleotide polymorphisms (SNPs) associated with 486 serum metabolites via high-throughput metabolic profiling. The cohort comprised 7824 participants, including 1768 individuals from Germany and 6056 from the United Kingdom. All participants provided written informed consent, and ethical approval was granted by local ethics committees. Of the 486 metabolites, 177 were uncharacterized, whereas 309 identified metabolites were classified into 8 distinct categories: carbohydrates, amino acids, cofactors and vitamins, lipids, peptides, energy products, nucleotides, and xenobiotics metabolites. The CML GWAS data were derived from the R12 version of the FinnGen database (https://www.finngen.fi/en).^[15]^ The dataset IDs were CML (https://storage.googleapis.com/finngen-public-data-r12/summary_stats/release/finngen_R12_CML.gz). These datasets included 301 adult CML cases and 497,256 controls.

### 2.3. Selection of IVs

IVs were selected through the following steps. Initially, genetic instruments associated with the 486 metabolites were pinpointed using a genome-wide significance threshold (*P* < 1 × 10⁻⁵). Second, SNPs were selected for independence to mitigate the influence of linkage disequilibrium (LD) on the results. The LD threshold was set to *r*² < 0.001, with a 1000-kb window size to avoid LD-caused bias.^[16]^ Third, the strength of individual SNPs was evaluated through F-statistic computation, with SNPs retaining an F-statistic > 10 selected for downstream analysis.^[17]^ The F-statistic was computed using the formula *F* = *R*^2^ × (n−*k*−1)/ [*k* × (1−*R*^2^)], where *R*² represents the proportion of variance in the exposure variable explained by the IV, given by *R*² = 2 × (1−MAF) × MAF×β^2^. In this equation: n denotes the sample size specific to the exposure; *k* is the number of IVs; MAF is the minor allele frequency; and β corresponds to the effect size.^[17,18]^

### 2.4. Mendelian randomization analysis

In this study, the inverse variance weighted (IVW) method served as the primary analytical approach to evaluate the causal association between serum metabolites and CML. Under the assumption that each genetic variant adheres to the core assumptions of an IV, IVW method is regarded as generating the most precise causal effect estimates. Consequently, IVW has been widely accepted as the primary approach for evaluating causal relationships.^[19,20]^ Assuming all SNPs are valid IVs and independent, IVW approach constrains the regression intercept to zero and applies the reciprocal of the outcome variance as weights during model fitting.^[21]^ To assess the robustness of our findings, we performed additional analyses using MR-Egger and weighted median (WM) methods.^[22,23]^ MR-Egger regression generates an intercept and its associated *P*-value: an intercept close to zero reflects concordance with the IVW results, whereas a significant deviation from zero suggests underlying horizontal pleiotropy among IVs. The WM method estimates the causal effect size by calculating the WM value and subsequently ranking the effect estimates of individual SNPs. Compared with IVW approach, WM reduces the type I error rate under conditions of limited sample size. Assessing consistency by comparing the current results with those derived from the MR-Egger significantly strengthens the reliability of causal effect estimates. The MR-Steiger test is employed to assess the directionality of the IV’s effect on the outcome, mitigating potential reverse causation bias, thereby validating whether the results align with the initial hypothesis. A Steiger test *P*-value > .05 for the IV suggests the presence of reverse causality.^[24,25]^

### 2.5. Sensitivity analysis

Cochran Q test was employed to evaluate heterogeneity among the selected SNPs. A significant Q statistic (*P* < .05) indicates statistically significant heterogeneity in the effect estimates of the analyzed SNPs.^[26]^ Pleiotropy was typically evaluated via the MR-Egger regression intercept and MR pleiotropy residual sum and outlier (MR-PRESSO) test. MR-PRESSO, a novel MR method, enables the detection of horizontal pleiotropy outliers, thereby yielding precise causal effect estimates.^[27]^ Statistical significance (*P* < .05) in both the MR-Egger intercept and MR-PRESSO global test indicates the existence of pleiotropy.^[28,29]^ Moreover, a leave-one-out sensitivity analysis was carried out to examine whether the causal effect estimates were significantly influenced by any single SNP.^[28]^

### 2.6. Metabolic pathway and functional enrichment analyses

Metabolic pathway enrichment analysis was carried out via the MetaboAnalyst 6.0 platform (https://www.metaboanalyst.ca/).^[30]^ Employing the pathway enrichment module, this study sought to characterize novel metabolic pathways implicated in the biological processes underlying CML pathogenesis. In this analysis, the Kyoto encyclopedia of genes and genomes (KEGG) was chosen as the primary pathway database. Enrichment analysis was carried out using the hypergeometric test, where a significance threshold of 0.05 was set for the identification of statistically enriched metabolic pathways. To enhance the biological interpretation of metabolic pathways, we performed an indirect Gene Ontology (GO) enrichment analysis by mapping differentially abundant metabolites to their associated genes (e.g., enzymes, transporters) using public databases. This step aimed to characterize the functional roles of the genes underlying the identified metabolic pathways. We extracted 19 differentially abundant metabolites from our KEGG results and mapped them to human genes using the KEGG pathway database (for enzyme EC numbers) and the Human Metabolome Database (for transporter/gene symbols). Using the clusterProfiler package (v4.10.0) in R, we performed GO enrichment on the deduplicated list of 177 associated genes, with the background set to all human protein-coding genes. The hypergeometric test was used for enrichment calculation, with false discovery rate (FDR) correction (Benjamini–Hochberg method) applied to control for multiple testing (FDR < 0.05).

## 3. Results

### 3.1. Strength of the IVs

We performed a 2-sample MR analysis to investigate the causal associations between serum metabolites and CML by leveraging GWAS summary datasets. Among the 486 analyzed metabolites, each was associated with IVs derived from 3 to 307 SNPs (fructose and ergothioneine yielded the least IVs: 3 SNPs, and 2-methoxyacetaminophen sulfate* produced the most IVs: 307 SNPs). Additionally, the smallest F-statistic across all IVs was 17.45, which confirmed the robustness of all IVs for the 2-sample MR analysis of the 486 metabolites (F-statistic > 10).

### 3.2. Mendelian randomization analysis results

**Figure 2.**
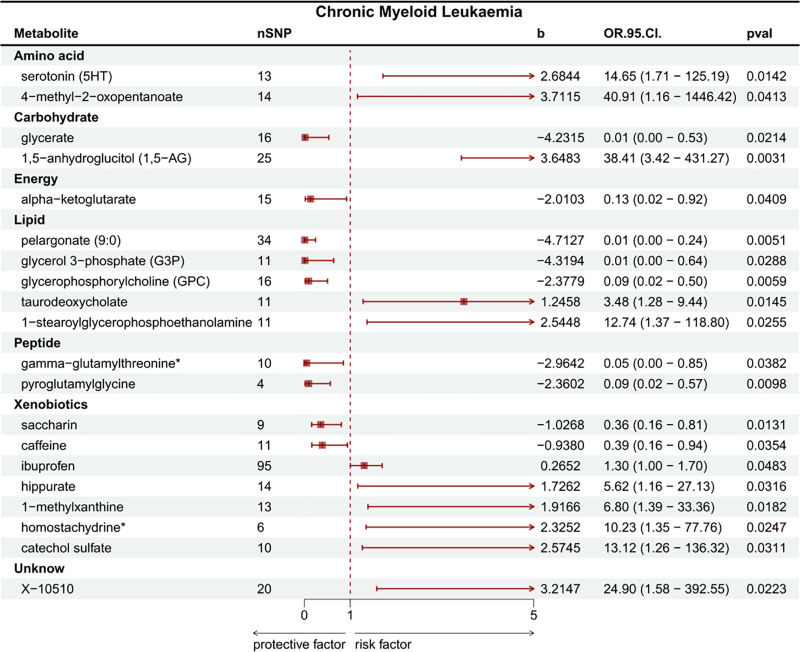
MR analysis of serum metabolites and CML. CML = chronic myeloid leukemia, MR = Mendelian randomization.

**Figure 3.**
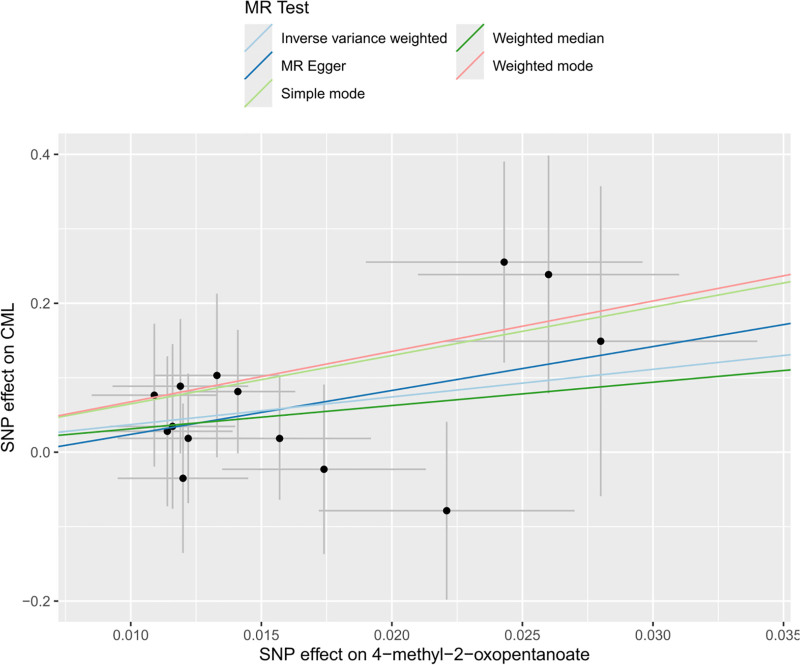
Scatter plot of the genetic association between 4-methyl-2-oxopentanoate and CML risk. CML = chronic myeloid leukemia.

In the investigative analysis of 486 metabolites via MR analysis employing the IVW method, 20 metabolites were identified as significantly associated with CML (of which 19 were identified metabolites and 1 were unidentified), as illustrated in Figure 2 and Table 1. Ten identified metabolites positively associated with CML were ibuprofen (OR: 1.3037, 95% CI: 1.0019–1.6963, *P* = .0484), taurodeoxycholate (OR: 3.4757, 95% CI: 1.2800–9.4379, *P* = .0145), Hippurate (OR: 5.6192, 95% CI: 1.1638–27.1300, *P* = .0316), 1-methylxanthine (OR: 6.7978, 95% CI: 1.3852–33.3586, *P* = .0182), homostachydrine* (OR: 10.2288, 95% CI: 1.3456–77.7592, *P* = .0247), 1-stearoylglycerophosphoethanolamine (OR: 12.7403, 95% CI: 1.3663–118.7996, *P* = .0255), catechol sulfate (OR: 13.1243, 95% CI: 1.2636–136.3190, *P* = .0311), serotonin (5HT) (OR: 14.6495, 95% CI: 1.7142–125.1939, *P* = .0142), 1,5-anhydroglucitol (OR: 38.4103, 95% CI: 3.4210–431.2691, *P* = .0031), and 4-methyl-2-oxopentanoate (OR: 40.9140, 95% CI: 1.1573–1446.4180, *P* = .0413), the unidentified metabolites positively associated with CML was X-10510 (OR: 24.8966, 95% CI: 1.5790–392.5455, *P* = .0223). The remaining 9 identified metabolites demonstrated a significant association with a reduced risk of CML. These include pelargonate (9:0) (OR: 0.0090, 95% CI: 0.0003–0.2432, *P* = .0051), glycerol 3-phosphate (G3P) (OR: 0.0133, 95% CI: 0.0003–0.6399, *P* = .0288), glycerate (OR: 0.0145, 95% CI: 0.0004–0.5343, *P* = .0214), gamma-glutamylthreonine* (OR: 0.0516, 95% CI: 0.0031–0.8514, *P* = .0382), glycerophosphorylcholine (OR: 0.0928, 95% CI: 0.0171–0.5042, *P* = .0059), pyroglutamylglycine (OR: 0.0944, 95% CI: 0.0158–0.5658, *P* = .0098), alpha-ketoglutarate (OR: 0.1340, 95% CI: 0.0200–0.9206, *P* = .0409), saccharin (OR: 0.3582, 95% CI: 0.1591–0.8064, *P* = .0132), and caffeine (OR: 0.3914, 95% CI: 0.1634: 0.9378, *P* = .0354). Among these, the amino acid metabolite 4-methyl-2-oxopentanoate displayed the strongest positive causal association with CML onset, while elevated levels of lipid metabolite pelargonate (9:0) showed the most significant negative causal association with CML (Fig. 3 and Fig. S1, Supplemental Digital Content, https://links.lww.com/MD/Q342).

**Table 1:** MR model estimated causal relationship between 20 metabolites and the risk of CML and tested for heterogeneity and horizontal pleiotropy.

Metabolite	nSNP	Cochran Q test		MR-Egger intercept		MR-Presso	
		IVW	MR-Egger	Egger intercept	*P*	Global Test RSSobs	*P*
1,5-anhydroglucitol (1,5-AG)	25	0.148	0.118	−0.003	.966	33.437	.176
1-methylxanthine	13	0.794	0.742	0.021	.656	8.990	.829
1-stearoylglycerophosphoethanolamine	11	0.415	0.573	0.126	.136	12.553	.466
4-methyl-2-oxopentanoate	14	0.928	0.899	−0.035	.726	7.841	.920
alpha-ketoglutarate	15	0.925	0.976	0.073	.158	8.451	.935
caffeine	11	0.999	0.996	0.013	.917	1.952	.998
catechol sulfate	10	0.363	0.527	0.117	.135	12.758	.411
gamma-glutamylthreonine*	10	0.401	0.368	0.073	.444	12.678	.356
glycerate	16	0.349	0.617	-0.182	.049	18.533	.404
glycerol 3-phosphate (G3P)	11	0.516	0.690	−0.128	.136	11.641	.536
glycerophosphorylcholine (GPC)	16	0.177	0.134	0.001	.982	22.765	.345
hippurate	14	0.203	0.240	−0.081	.242	18.983	.245
homostachydrine*	6	0.547	0.405	0.011	.940	5.4890	.599
ibuprofen	95	0.736	0.713	−0.013	.757	86.819	.729
pelargonate (9:0)	34	0.234	0.203	0.022	.728	40.624	.270
pyroglutamylglycine	4	0.870	0.927	−0.123	.532	1.347	.887
saccharin	9	0.356	0.478	−0.113	.174	10.758	.427
serotonin (5HT)	13	0.299	0.237	−0.015	.790	15.689	.365
taurodeoxycholate	11	0.461	0.373	0.024	.841	11.864	.535
X-10510	20	0.857	0.827	−0.028	.625	13.855	.870

CML = chronic myeloid leukemia, IVW = inverse variance weighted, MR = Mendelian randomization, SNPs = single nucleotide polymorphisms.

* Indicates metabolites for which reference spectra of the pure substances were not directly measured on the Metabolon platform.

### 3.3. Sensitivity and reverse causality analysis results

When assessing the heterogeneity among the 20 metabolites significantly associated with CML, no significant heterogeneity was detected in both IVW and MR-Egger models via Cochran Q test (*P* > .05) (Table 1). Furthermore, MR-PRESSO analysis did not identify any outliers, with both the global test *P*-value and the MR-Egger intercept *P*-value exceeding .05, which indicated no significant horizontal pleiotropy (Fig. S2, Supplemental Digital Content, https://links.lww.com/MD/Q342). Notably, the MR-Egger intercept *P*-value for the metabolite glycerate was .0493, slightly below the significance threshold, indicating a potential level of pleiotropy. Leave-one-out analysis further confirmed that the causal effects of individual metabolites were not dominated by any SNP, thereby strengthening confidence in the robustness of the analytical results (Fig. S3, Supplemental Digital Content, https://links.lww.com/MD/Q342). Additionally, The MR-Steiger directionality test validated the exposure-outcome directionality across all IVs, with each demonstrating *P*_Steiger_ < .05. *P*-values from tests for reverse association between individual metabolites and CML were all <.05, indicating no evidence of potential reverse causation. This conclusion was further corroborated by IVW analyses, which yielded concordant results, strengthening the robustness of our findings. Detailed results are provided in Table S1, Supplemental Digital Content, https://links.lww.com/MD/Q341.

### 3.4. Metabolic pathway and functional enrichment analyses

**Figure 4.**
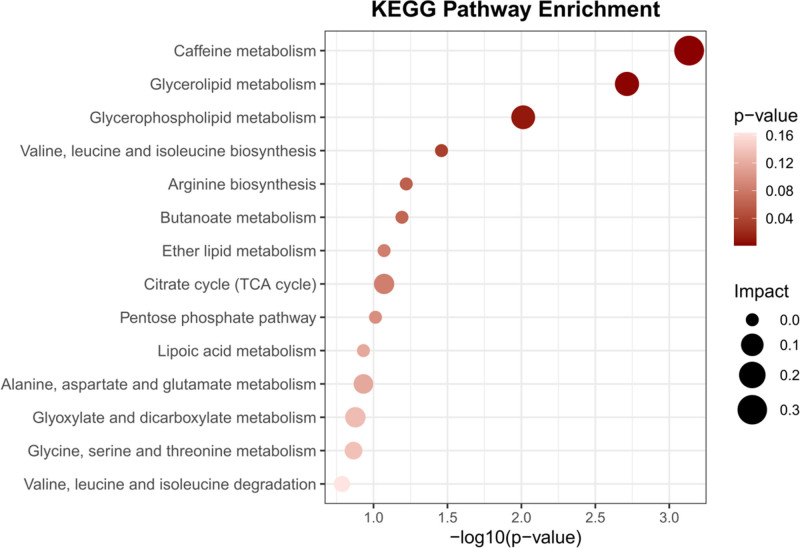
Displays the enrichment pathways of metabolites in KEGG. KEGG = Kyoto encyclopedia of genes and genomes.

Metabolic pathway analysis identified 4 key metabolic pathways primarily associated with CML (Fig. 4 and Table S2, Supplemental Digital Content, https://links.lww.com/MD/Q341). Enrichment analysis results further underscored these critical pathways, including the caffeine metabolism, glycerolipid metabolism, glycerophospholipid metabolism, and valine, leucine, and isoleucine biosynthesis. To further explore the biological relevance of the differentially abundant metabolites, we performed indirect GO enrichment analysis on their associated genes (as described in Materials and methods). This analysis identified a significant enrichment of several biological processes (FDR < 0.05), including glycerolipid metabolic process, glycerophospholipid metabolic process, glycerolipid catabolic process, and response to caffeine (Fig. S4, Supplemental Digital Content, https://links.lww.com/MD/Q342). These results align with our KEGG findings in CML pathogenesis. Specifically, the enrichment of cellular response to caffeine is consistent with KEGG’s caffeine metabolism enrichment, highlighting the potential role of caffeine metabolism pathways in CML pathogenesis. Additionally, the enrichment of glycerolipid metabolic process, consistent with KEGG’s identification of glycerolipid metabolism as a key pathway, suggests dysregulation in lipid synthesis or storage may contribute to leukemogenesis.

## 4. Discussion

To the best of our knowledge, no prior investigations have explored the causal associations between serum metabolites and CML using MR analysis. Our systematic investigation utilized MR analysis combined with pathway enrichment analysis to examine the relationships between serum metabolites and CML. Utilizing GWAS data, our investigation identified 20 serum metabolites (19 known and 1 unknown) associated with CML from a pool of 486 candidate metabolites. Among the identified metabolites, 10 metabolites exhibited adverse associations with an elevated risk of CML, whereas 9 metabolites exhibited protective associations with reduced CML risk. Furthermore, pathway enrichment analysis identified 4 significantly enriched metabolic pathways: caffeine metabolism, glycerolipid metabolism, glycerophospholipid metabolism, and valine, leucine, and isoleucine biosynthesis, which are predominantly associated with CML.

Our study identified elevated levels of amino acid metabolite 4-methyl-2-oxopentanoate as the strongest risk factor for CML onset. 4-methyl-2-oxopentanoate, an atypical metabolite of leucine, together with branched-chain amino acids (BCAA), has been documented to contribute to adverse effects on multiple diseases. Recent mediation analysis conducted in a Japanese cohort demonstrated that 4-methyl-2-oxopentanoate and BCAA mediated over 20% of the association between the cardio-ankle vascular index and the nonalcoholic fatty liver disease.^[31]^ 4-Methyl-2-oxopentanoate plays a critical role in energy production, cholesterol biosynthesis, and the metabolism of other isoprenoid compounds.^[32,33]^ Elevated circulating levels of 4-methyl-2-oxopentanoate have been demonstrated to correlate with type 2 diabetes mellitus and insulin resistance in both human and animal models.^[34,35]^ Previous study has demonstrated that BCAA induce inflammation, oxidative stress, and migration of human peripheral blood mononuclear cells.^[36]^ A study has demonstrated that the ratio of α-ketobutyrate to 4-methyl-2-oxopentanoate reflects energy metabolism, as well as the metabolic profiles of serine, alanine, and isoleucine, thereby regulating CCR2 expression and activity, and subsequently influencing monocyte migration and inflammatory responses.^[37,38]^ Previous studies have suggested that 4-methyl-2-oxopentanoate may play a critical role in pro-inflammatory processes. In our investigation, we identified 4-methyl-2-oxopentanoate as the strongest risk factor for CML, which implies its potential contribution to the pro-inflammatory pathogenesis of CML and positions it as a key biomarker for CML. The above findings indicated that 4-methyl-2-oxopentanoate may play a critical role in pro-inflammatory processes. In our study, we identified 4-methyl-2-oxopentanoate as the strongest risk factor for CML. This metabolite might contribute to CML progression through pro-inflammatory responses and serve as a key biomarker for CML. To gain deeper insights into the role of metabolites in CML, further clinical research and experimental verification were necessary.

The metabolites identified in our study were significantly enriched in the caffeine metabolism, glycerolipid metabolism, glycerophospholipid metabolism, and valine, leucine, and isoleucine biosynthesis. Caffeine metabolism was the most significantly enriched metabolic pathways. Caffeine is a xanthine alkaloid that exerts its stimulatory effects via adenosine receptors. Prenatal exposure to caffeine (including intake) in pregnant women has been suggested to play a potential role in neonatal leukemia risk.^[39]^ Menegaux et al investigated maternal coffee consumption, alcohol intake, and parental smoking as potential risk factors for childhood leukemia. Their findings indicated a potential association between high maternal coffee consumption (defined as ≥3 cups per day) and an increased risk of acute leukemia.^[40]^ The study proposed that caffeine may modulate fetal development via its impacts on DNA replication and repair mechanisms, potentially inducing oncogenic mutations. Bonaventure et al explored the interaction between maternal caffeine intake and genetic susceptibility in a case-control study. Their results revealed an elevated risk of acute lymphoblastic leukemia (ALL) in offspring of mothers with heightened caffeine consumption during pregnancy, particularly among children harboring-specific metabolic polymorphisms that modulate caffeine metabolism. These findings underscored the critical role of gene–environment interactions in regulating leukemia susceptibility.^[41]^ Milne et al reported a significantly increased risk of ALL in children associated with higher maternal coffee consumption, with evidence of a dose-dependent association. This study strengthened the hypothesis that caffeine exposure may impair fetal hematopoiesis or disrupt immune system development, potentially elevating leukemia susceptibility.^[42]^ Their findings further validated a modest yet statistically significant elevation in ALL risk associated with higher maternal coffee consumption during pregnancy.^[43,44]^ The authors hypothesized that caffeine’s capacity to induce oxidative stress and disrupt DNA integrity may contribute to the pathogenesis of leukemia. In summary, caffeine metabolism may contribute to CML development through mechanisms involving the induction of oxidative stress, disruption of DNA integrity, impairment of DNA replication and repair, and disruption of hematopoietic or immune system development.

Our study has several limitations. First, the statistical power of IVs is primarily determined by the sample size of GWASs; thus, larger datasets are required to enhance the precision of CML risk predictions. Second, the validity of MR analysis relies heavily on the explanatory power of IVs for the exposure. Consequently, expanding the sample size is critical to enable more precise evaluation of the genetic influences on metabolite levels. Third, although MR has been validated as a robust approach for inferring causal relationships between serum metabolites and CML, its findings necessitate validation through additional experimental studies. Fourth, the current dataset is derived from individuals of European ancestry, which may limit the generalizability of the results to non-European populations. Therefore, extending the analysis to include individuals of diverse genetic backgrounds is warranted to improve the broader applicability of our conclusions. Fifth, our analysis integrated KEGG pathway and GO enrichment analyses. However, the lack of direct gene expression data limits the GO analysis, potentially missing functional insights from transcriptionally active genes. This may overlook context-specific biological processes relevant to CML. Future studies will integrate GO and single-sample Gene Set Enrichment Analysis using multi-omics data (e.g., transcriptomics) to provide a more comprehensive functional interpretation of the identified metabolic pathways. Finally, while our study identified multiple metabolites associated with increased CML risk, further investigations are needed to elucidate their specific roles in the pathogenesis of CML.

## 5. Conclusion

In conclusion, this MR study identified a total of 20 metabolites exhibiting potential causal associations with CML pathogenesis, comprising 19 known and 1 unknown metabolites. The amino acid metabolite 4-methyl-2-oxopentanoate emerged as the key risk driver for CML, potentially promoting disease progression through pro-inflammatory mechanisms. Additionally, 4 key metabolic pathways potentially implicated in CML pathogenesis were uncovered. Collectively, these findings provide critical insights into the potential application of these identified biomarkers for developing targeted therapeutic strategies against CML.

## Acknowledgments

We wish to express our gratitude to the researchers who performed the initial GWAS studies and shared their summary statistics with the public.

## Author contributions

**Conceptualization:** Xiaoli Li.

**Data curation:** Haohan Ye.

**Formal analysis:** Haohan Ye, Jun Tang, Yuanheng Liu.

**Funding acquisition:** Xiaoli Li.

**Investigation:** Haohan Ye.

**Project administration:** Xiaoli Li.

**Resources:** Xiaoli Li.

**Software:** Haohan Ye.

**Supervision:** Xiaoli Li.

**Validation:** Haohan Ye.

**Writing – original draft:** Xiaoli Li, Haohan Ye.

**Writing – review & editing:** Xiaoli Li.

## Supplementary Material





## References

[1] Minciacchi VR Kumar R Krause DS. Chronic myeloid leukemia: a model disease of the past, present and future. Cells. 2021;10:117. 33435150 10.3390/cells10010117 PMC7827482

[2] Sung H Ferlay J Siegel RL. Global Cancer Statistics 2020: Globocan estimates of incidence and mortality worldwide for 36 cancers in 185 countries. CA Cancer J Clin. 2021;71:209–49. 33538338 10.3322/caac.21660

[3] Baccarani M Deininger MW Rosti G. European leukemianet recommendations for the management of chronic myeloid leukemia: 2013. Blood. 2013;122:872–84. 23803709 10.1182/blood-2013-05-501569 PMC4915804

[4] Ito K Ito K. Leukemia stem cells as a potential target to achieve therapy-free remission in chronic myeloid leukemia. Cancers (Basel). 2021;13:5822. 34830976 10.3390/cancers13225822 PMC8616035

[5] Jiang CF. Platelet indices and the causal relationship with myeloid leukemia: a Mendelian randomization study with dual samples. Clin Lab. 2025;71:e240733. 10.7754/Clin.Lab.2024.240733 39808133

[6] Caocci G Deidda M Noto A. Metabolomic analysis of patients with chronic myeloid leukemia and cardiovascular adverse events after treatment with tyrosine kinase inhibitors. J Clin Med. 2020;9:1180. 32326001 10.3390/jcm9041180 PMC7231160

[7] Karlíková R Široká J Friedecký D. Metabolite profiling of the plasma and leukocytes of chronic myeloid leukemia patients. J Proteome Res. 2016;15:3158–66. 27465658 10.1021/acs.jproteome.6b00356

[8] Vonka V Humlova Z Klamova H. Kynurenine and uric acid levels in chronic myeloid leukemia patients. Oncoimmunology. 2015;4:e992646. 25949913 10.4161/2162402X.2014.992646 PMC4404910

[9] Yang B Wang C Xie Y Xu L Wu X Wu D. Monitoring tyrosine kinase inhibitor therapeutic responses with a panel of metabolic biomarkers in chronic myeloid leukemia patients. Cancer Sci. 2018;109:777–84. 29316075 10.1111/cas.13500 PMC5834806

[10] Greenland S. An introduction to instrumental variables for epidemiologists. Int J Epidemiol. 2018;47:358. 29294084 10.1093/ije/dyx275

[11] Hemani G Zheng J Elsworth B. The Mr-Base platform supports systematic causal inference across the human phenome. Elife. 2018;7:e34408. 29846171 10.7554/eLife.34408 PMC5976434

[12] Zhuang X Zhang X Yin Q. Causal pathways in lymphoma: the role of serum metabolites and immune cells determined by Mendelian randomization. Int Immunopharmacol. 2025;144:113593. 39591822 10.1016/j.intimp.2024.113593

[13] Chen G Kuang Z Li F Li J. The causal relationship between gut microbiota and leukemia: a two-sample Mendelian randomization study. Front Microbiol. 2023;14:1293333. 38075916 10.3389/fmicb.2023.1293333 PMC10703164

[14] Shin SY Fauman EB Petersen AK. An atlas of genetic influences on human blood metabolites. Nat Genet. 2014;46:543–50. 24816252 10.1038/ng.2982 PMC4064254

[15] Kurki MI Karjalainen J Palta P. Finngen provides genetic insights from a well-phenotyped isolated population. Nature. 2023;613:508–18. 36653562 10.1038/s41586-022-05473-8 PMC9849126

[16] Hou Y Xiao Z Zhu Y Li Y Liu Q Wang Z. Blood metabolites and chronic kidney disease: a Mendelian randomization study. BMC Med Genomics. 2024;17:147. 38807172 10.1186/s12920-024-01918-3 PMC11131213

[17] Papadimitriou N Dimou N Tsilidis KK. Physical activity and risks of breast and colorectal cancer: a Mendelian randomisation analysis. Nat Commun. 2020;11:597. 32001714 10.1038/s41467-020-14389-8 PMC6992637

[18] Gill D Efstathiadou A Cawood K Tzoulaki I Dehghan A. Education protects against coronary heart disease and stroke independently of cognitive function: evidence from Mendelian randomization. Int J Epidemiol. 2019;48:1468–77. 31562522 10.1093/ije/dyz200 PMC6857750

[19] Burgess S Butterworth A Thompson SG. Mendelian randomization analysis with multiple genetic variants using summarized data. Genet Epidemiol. 2013;37:658–65. 24114802 10.1002/gepi.21758 PMC4377079

[20] Li S Xu Y Zhang Y. Mendelian randomization analyses of genetically predicted circulating levels of cytokines with risk of breast cancer. npj Precis Oncol. 2020;4:25. 32923685 10.1038/s41698-020-00131-6 PMC7462857

[21] Pagoni P Korologou-Linden RS Howe LD. Causal effects of circulating cytokine concentrations on risk of Alzheimer’s disease and cognitive function. Brain Behav Immun. 2022;104:54–64. 35580794 10.1016/j.bbi.2022.05.006 PMC10391322

[22] Bowden J Davey Smith G Burgess S. Mendelian randomization with invalid instruments: effect estimation and bias detection through egger regression. Int J Epidemiol. 2015;44:512–25. 26050253 10.1093/ije/dyv080 PMC4469799

[23] Bowden J Davey Smith G Haycock PC Burgess S. Consistent estimation in Mendelian randomization with some invalid instruments using a weighted median estimator. Genet Epidemiol. 2016;40:304–14. 27061298 10.1002/gepi.21965 PMC4849733

[24] Hemani G Tilling K Davey Smith G. Orienting the causal relationship between imprecisely measured traits using gwas summary data. PLoS Genet. 2017;13:e1007081. 29149188 10.1371/journal.pgen.1007081 PMC5711033

[25] Xiao G He Q Liu L. Causality of genetically determined metabolites on anxiety disorders: a two-sample Mendelian randomization study. J Transl Med. 2022;20:475. 36266699 10.1186/s12967-022-03691-2 PMC9583573

[26] Greco MF Minelli C Sheehan NA Thompson JR. Detecting pleiotropy in Mendelian randomisation studies with summary data and a continuous outcome. Stat Med. 2015;34:2926–40. 25950993 10.1002/sim.6522

[27] Verbanck M Chen CY Neale B Do R. Detection of widespread horizontal pleiotropy in causal relationships inferred from Mendelian randomization between complex traits and diseases. Nat Genet. 2018;50:693–8. 29686387 10.1038/s41588-018-0099-7 PMC6083837

[28] Burgess S Thompson SG. Interpreting findings from Mendelian randomization using the Mr-Egger method. Eur J Epidemiol. 2017;32:377–89. 28527048 10.1007/s10654-017-0255-x PMC5506233

[29] Su D Ai Y Zhu G Yang Y Ma P. Genetically predicted circulating levels of cytokines and the risk of osteoarthritis: a Mendelian randomization study. Front Genet. 2023;14:1131198. 36999058 10.3389/fgene.2023.1131198 PMC10043178

[30] Pang Z Lu Y Zhou G. Metaboanalyst 6.0: towards a unified platform for metabolomics data processing, analysis and interpretation. Nucleic Acids Res. 2024;52:W398–406. 38587201 10.1093/nar/gkae253 PMC11223798

[31] Hirata A Harada S Iida M. Association of nonalcoholic fatty liver disease with arterial stiffness and its metabolomic profiling in Japanese community-dwellers. J Atheroscler Thromb. 2024;31:1031–47. 38311416 10.5551/jat.64616 PMC11224684

[32] Wang L Yao J Tu T Yao B Zhang J. Heterotrophic and autotrophic production of L-Isoleucine and L-Valine by engineered cupriavidus necator H16. Bioresour Technol. 2024;398:130538. 38452952 10.1016/j.biortech.2024.130538

[33] Xu H Zhang Y Guo X. Isoleucine biosynthesis in leptospira interrogans serotype lai strain 56601 proceeds via a threonine-independent pathway. J Bacteriol. 2004;186:5400–9. 15292141 10.1128/JB.186.16.5400-5409.2004 PMC490871

[34] Giesbertz P Padberg I Rein D. Metabolite profiling in plasma and tissues of Ob/Ob and Db/Db mice identifies novel markers of obesity and type 2 diabetes. Diabetologia. 2015;58:2133–43. 26058503 10.1007/s00125-015-3656-y

[35] Perng W Gillman MW Fleisch AF. Metabolomic profiles and childhood obesity. Obesity (Silver Spring). 2014;22:2570–8. 25251340 10.1002/oby.20901 PMC4236243

[36] Zhenyukh O Civantos E Ruiz-Ortega M. High concentration of branched-chain amino acids promotes oxidative stress, inflammation and migration of human peripheral blood mononuclear cells via mtorc1 activation. Free Radic Biol Med. 2017;104:165–77. 28089725 10.1016/j.freeradbiomed.2017.01.009

[37] Yang L Chu Z Liu M. Amino acid metabolism in immune cells: essential regulators of the effector functions, and promising opportunities to enhance cancer immunotherapy. J Hematol Oncol. 2023;16:59. 37277776 10.1186/s13045-023-01453-1 PMC10240810

[38] Lynch CJ Adams SH. Branched-chain amino acids in metabolic signalling and insulin resistance. Nat Rev Endocrinol. 2014;10:723–36. 25287287 10.1038/nrendo.2014.171 PMC4424797

[39] Dube R Kar SS Bahutair SNM. The fetal effect of maternal caffeine consumption during pregnancy-a review. Biomedicines. 2025;13:390. 40002803 10.3390/biomedicines13020390 PMC11852448

[40] Menegaux F Ripert M Hémon D Clavel J. Maternal alcohol and coffee drinking, parental smoking and childhood leukaemia: a french population-based case-control study. Paediatr Perinat Epidemiol. 2007;21:293–9. 17564585 10.1111/j.1365-3016.2007.00824.x

[41] Bonaventure A Rudant J Goujon-Bellec S. Childhood acute leukemia, maternal beverage intake during pregnancy, and metabolic polymorphisms. Cancer Causes Control. 2013;24:783–93. 23404349 10.1007/s10552-013-0161-9

[42] Gaskins AJ Rich-Edwards JW Williams PL Toth TL Missmer SA Chavarro JE. Pre-pregnancy caffeine and caffeinated beverage intake and risk of spontaneous abortion. Eur J Nutr. 2018;57:107–17. 27573467 10.1007/s00394-016-1301-2 PMC5332346

[43] Milne E Greenop KR Petridou E. Maternal consumption of coffee and tea during pregnancy and risk of childhood all: a pooled analysis from the childhood leukemia international consortium. Cancer Causes Control. 2018;29:539–50. 29600472 10.1007/s10552-018-1024-1

[44] Milne E Royle JA Bennett LC. Maternal consumption of coffee and tea during pregnancy and risk of childhood all: results from an Australian case-control study. Cancer Causes Control. 2011;22:207–18. 21113653 10.1007/s10552-010-9688-1

