# Supplementary material for: Causal relationship between serum metabolites and chronic myeloid leukemia: A bidirectional Mendelian randomization study
Source: Medicine (Baltimore). 2025 Oct 10;104(41):e45217. doi: 10.1097/MD.0000000000045217 (PMC12517889; doi:10.1097/MD.0000000000045217)
Supplement: Supplementary file 1 [file medi-104-e45217-s001.docx]

Table S1 The reverse MR analysis of association metabolites and CML using two methods.

| Metabolite | nSNP |  | IVW | |  | Steiger |
| --- | --- | --- | --- | --- | --- | --- |
|  |  |  | b | pval |  | pval |
| 1,5-anhydroglucitol (1,5-AG) | 25 |  | -0.002 | 0.759 |  | 2.58E-127 |
| 1-methylxanthine | 13 |  | 0.012 | 0.300 |  | 5.77E-113 |
| 1-stearoylglycerophosphoethanolamine | 11 |  | 0.001 | 0.893 |  | 2.22E-57 |
| 4-methyl-2-oxopentanoate | 14 |  | 0.000 | 0.963 |  | 1.16E-70 |
| alpha-ketoglutarate | 15 |  | -0.005 | 0.661 |  | 2.97E-72 |
| caffeine | 11 |  | -0.001 | 0.988 |  | 8.98E-66 |
| catechol sulfate | 10 |  | 0.004 | 0.708 |  | 9.70E-44 |
| gamma-glutamylthreonine* | 10 |  | 0.004 | 0.622 |  | 7.12E-47 |
| glycerate | 16 |  | -0.011 | 0.064 |  | 1.87E-73 |
| glycerol 3-phosphate (G3P) | 11 |  | -0.012 | 0.063 |  | 7.92E-50 |
| glycerophosphorylcholine (GPC) | 16 |  | 0.002 | 0.855 |  | 1.63E-71 |
| hippurate | 14 |  | -0.001 | 0.912 |  | 2.97E-62 |
| homostachydrine* | 6 |  | 0.002 | 0.895 |  | 1.36E-27 |
| ibuprofen | 95 |  | -0.008 | 0.814 |  | 0.000 |
| pelargonate (9:0) | 34 |  | -0.006 | 0.381 |  | 1.68E-164 |
| pyroglutamylglycine | 4 |  | 0.031 | 0.140 |  | 1.03E-18 |
| saccharin | 9 |  | -0.019 | 0.501 |  | 8.50E-41 |
| serotonin (5HT) | 13 |  | -0.013 | 0.428 |  | 5.96E-60 |
| taurodeoxycholate | 11 |  | 0.015 | 0.563 |  | 8.00E-51 |
| X-10510 | 20 |  | -0.011 | 0.075 |  | 2.58E-127 |

Table S2. Metabolic pathway associated with CML.

| Pathway | Total | Expected | Hits | Raw p | FDR | Impact |
| --- | --- | --- | --- | --- | --- | --- |
| Caffeine metabolism | 10 | 0.044 | 2 | 0.001 | 0.059 | 0.308 |
| Glycerolipid metabolism | 16 | 0.070 | 2 | 0.002 | 0.077 | 0.137 |
| Glycerophospholipid metabolism | 36 | 0.158 | 2 | 0.010 | 0.259 | 0.128 |
| Valine, leucine and isoleucine biosynthesis | 8 | 0.035 | 1 | 0.035 | 0.694 | 0.000 |
| Arginine biosynthesis | 14 | 0.062 | 1 | 0.060 | 0.841 | 0.000 |
| Butanoate metabolism | 15 | 0.066 | 1 | 0.064 | 0.841 | 0.000 |
| Ether lipid metabolism | 20 | 0.088 | 1 | 0.085 | 0.841 | 0.000 |
| Citrate cycle (TCA cycle) | 20 | 0.088 | 1 | 0.085 | 0.841 | 0.059 |
| Pentose phosphate pathway | 23 | 0.101 | 1 | 0.097 | 0.841 | 0.000 |
| Lipoic acid metabolism | 28 | 0.123 | 1 | 0.117 | 0.841 | 0.000 |
| Alanine, aspartate and glutamate metabolism | 28 | 0.123 | 1 | 0.117 | 0.841 | 0.048 |
| Glyoxylate and dicarboxylate metabolism | 32 | 0.141 | 1 | 0.133 | 0.841 | 0.060 |
| Glycine, serine and threonine metabolism | 33 | 0.145 | 1 | 0.137 | 0.841 | 0.025 |
| Valine, leucine and isoleucine degradation | 40 | 0.176 | 1 | 0.164 | 0.934 | 0.011 |
