# Supplementary material for: Causal relationship between serum metabolites and chronic myeloid leukemia: A bidirectional Mendelian randomization study
Source: Medicine (Baltimore). 2025 Oct 10;104(41):e45217. doi: 10.1097/MD.0000000000045217 (PMC12517889; doi:10.1097/MD.0000000000045217)

Supplementary Figure S1. Scatter plots of the genetic association of remaining 19 metabolites on the risk of CML.

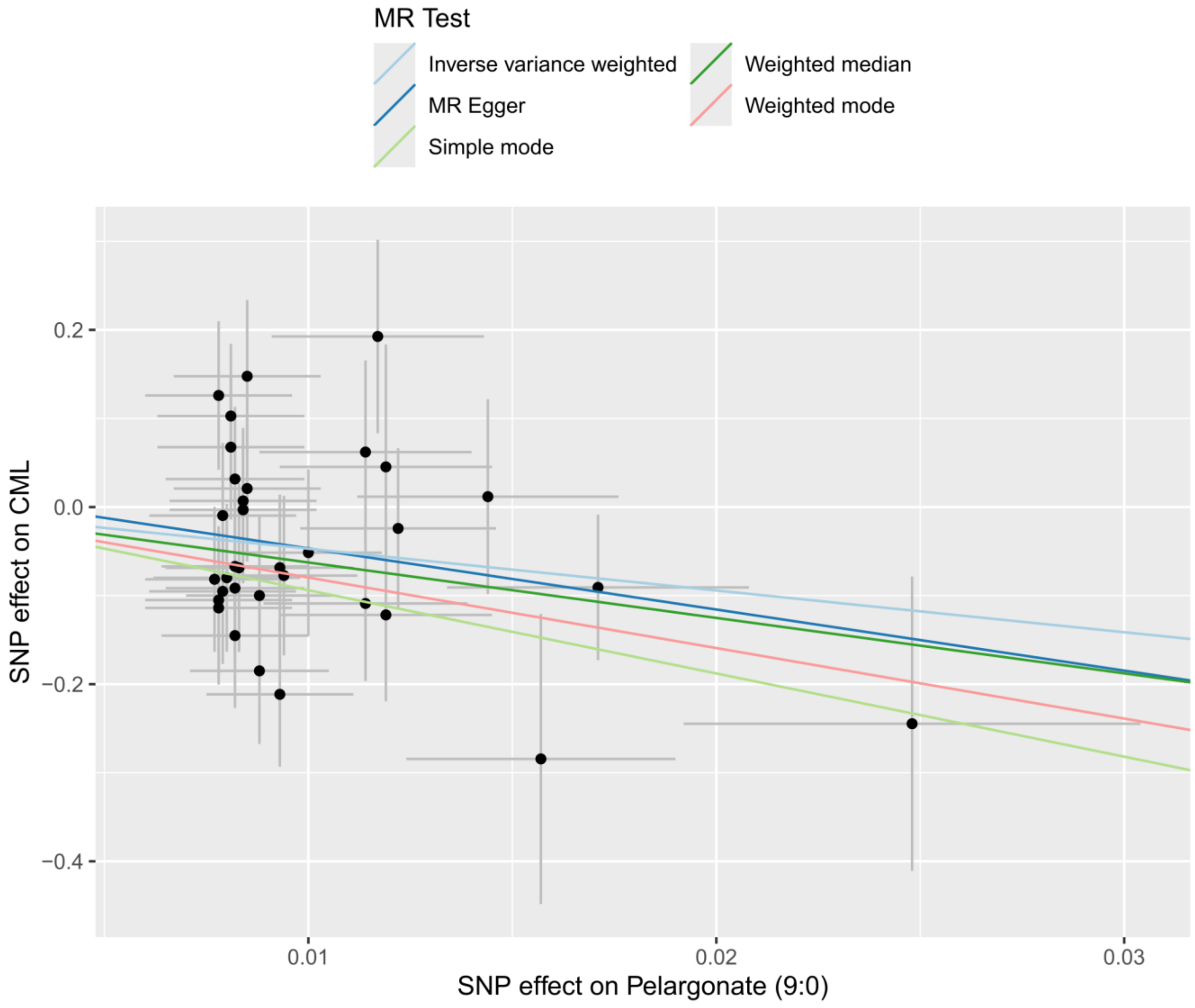

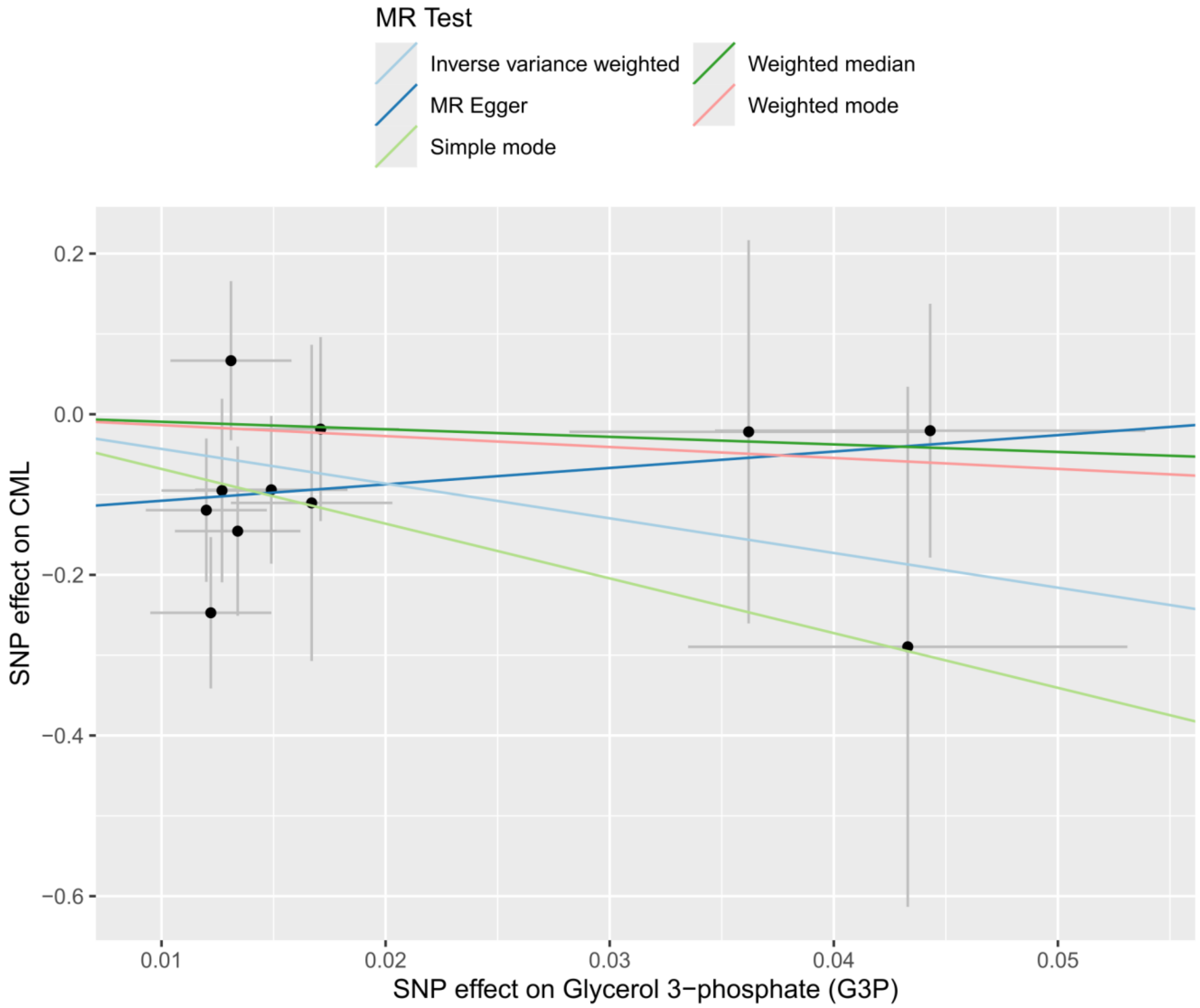
MR Test
Inverse variance weighted
MR Egger
Simple mode
Weighted median
Weighted mode
0.2
0.0
−0.2
−0.4
−0.6
SNP effect on CML
0.01
0.02
0.03
0.04
0.05
SNP effect on Glycerol 3−phosphate (G3P)

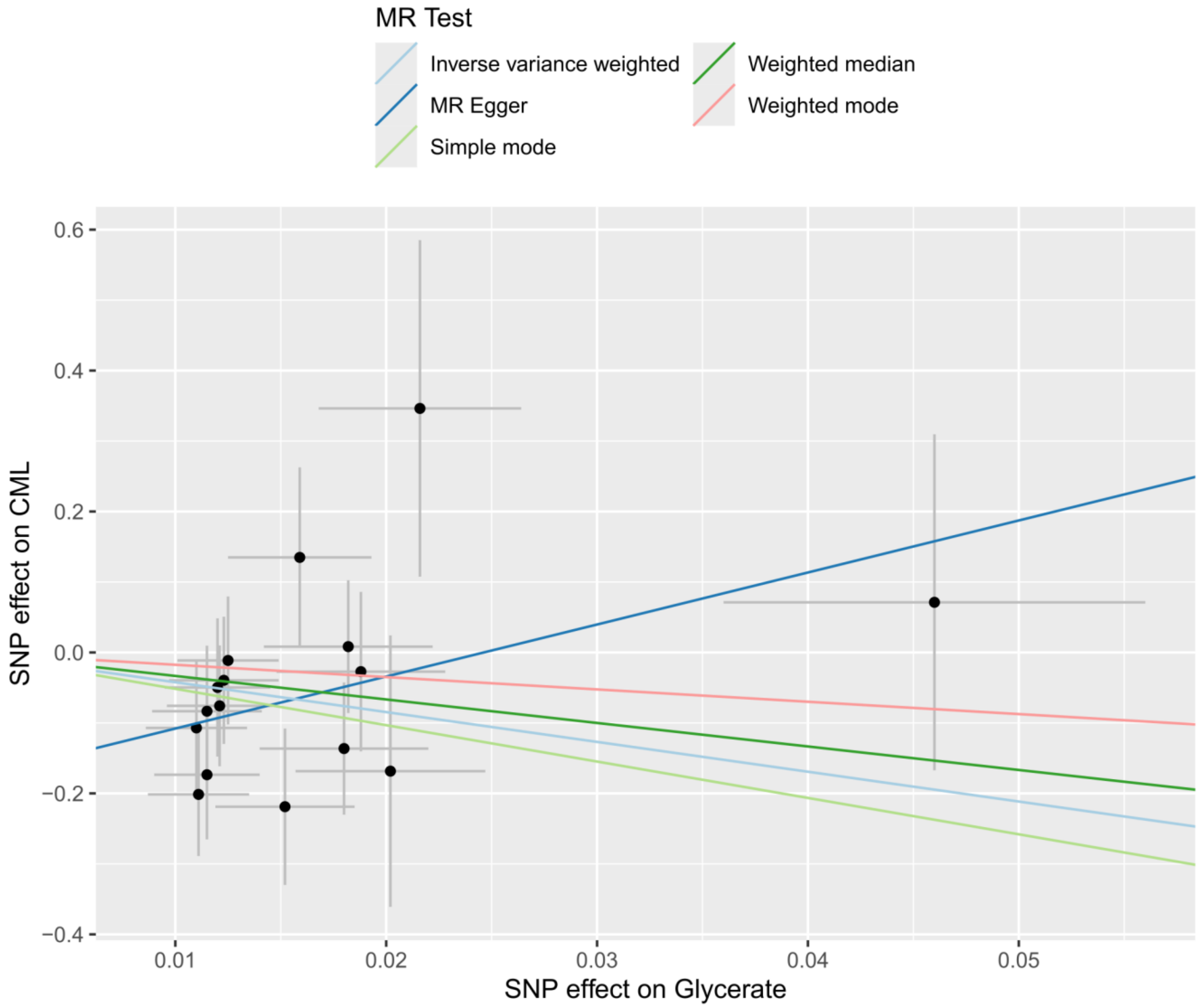

MR Test
Inverse variance weighted
MR Egger
Simple mode
Weighted median
Weighted mode
0.6
0.4
0.2
0.0
−0.2
−0.4
0.01
0.02
0.03
0.04
0.05
SNP effect on CML
SNP effect on Glycerate

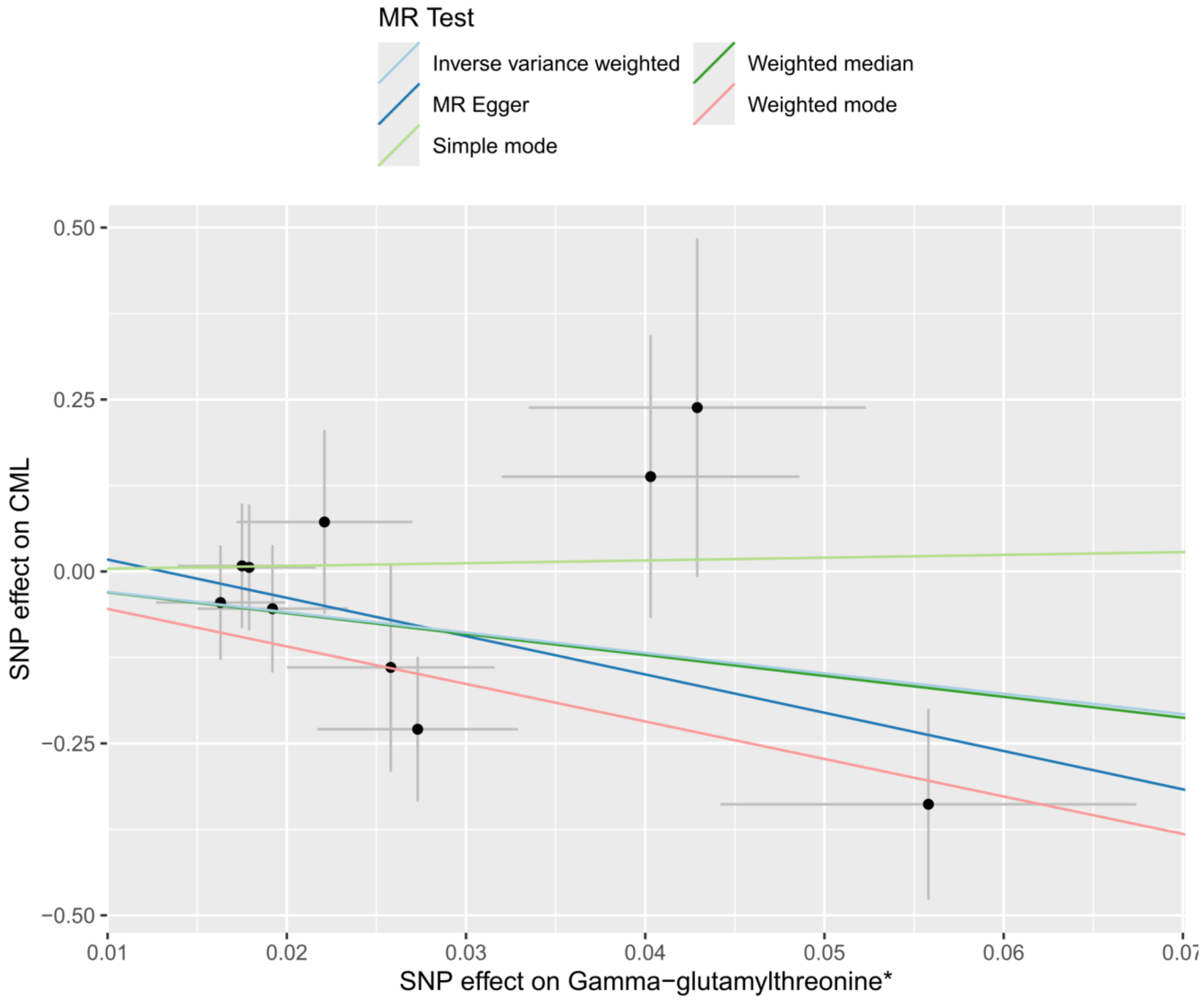

MR Test
Inverse variance weighted
MR Egger
Simple mode
Weighted median
Weighted mode
0.50
0.25
0.00
−0.25
−0.50
SNP effect on CML
0.01
0.02
0.03
0.04
0.05
0.06
SNP effect on Gamma−glutamylthreonine*

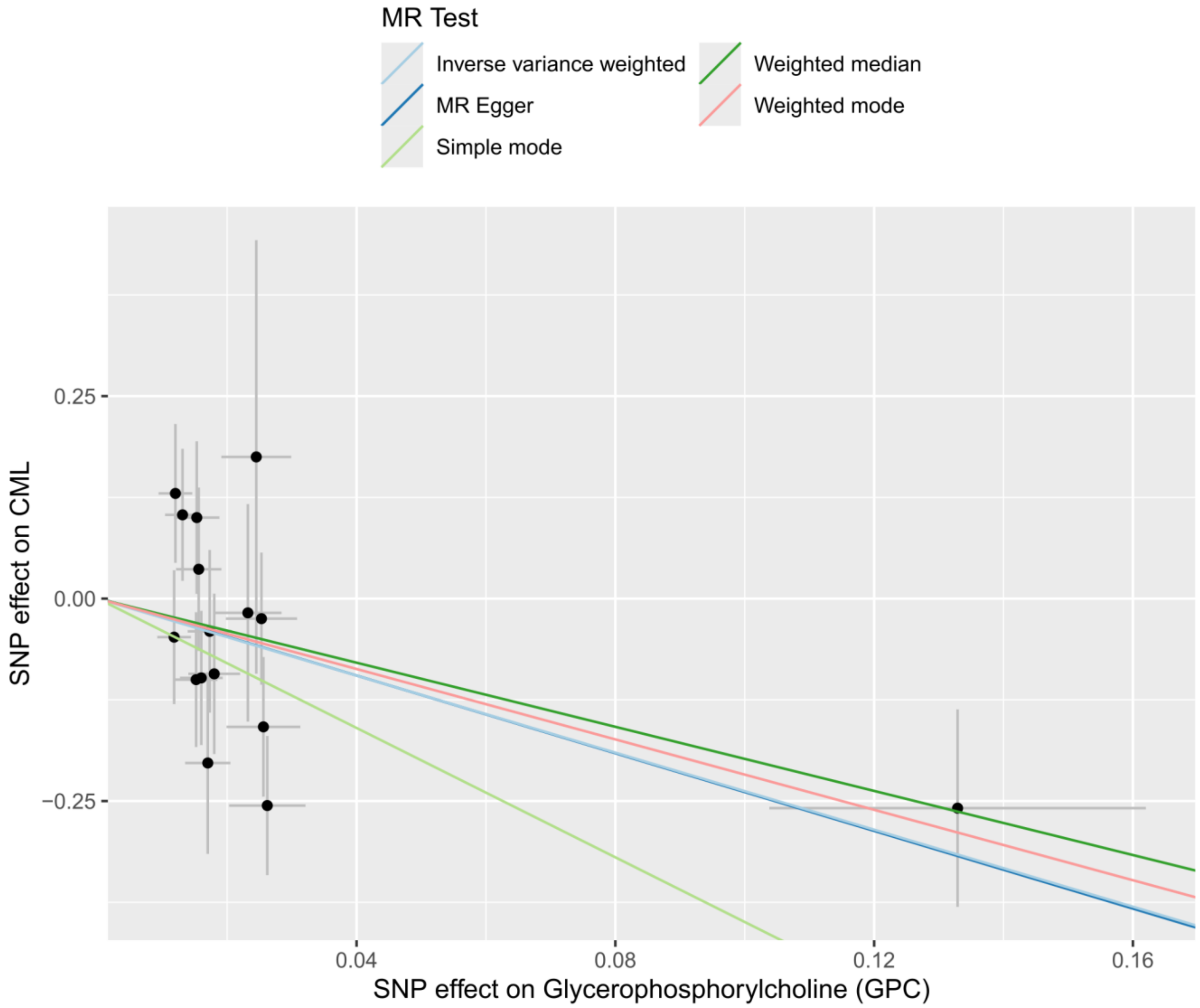
MR Test
Inverse variance weighted
MR Egger
Simple mode
Weighted median
Weighted mode
0.25
0.00
−0.25
0.04
0.08
0.12
0.16
SNP effect on CML
SNP effect on Glycerophosphorylcholine (GPC)

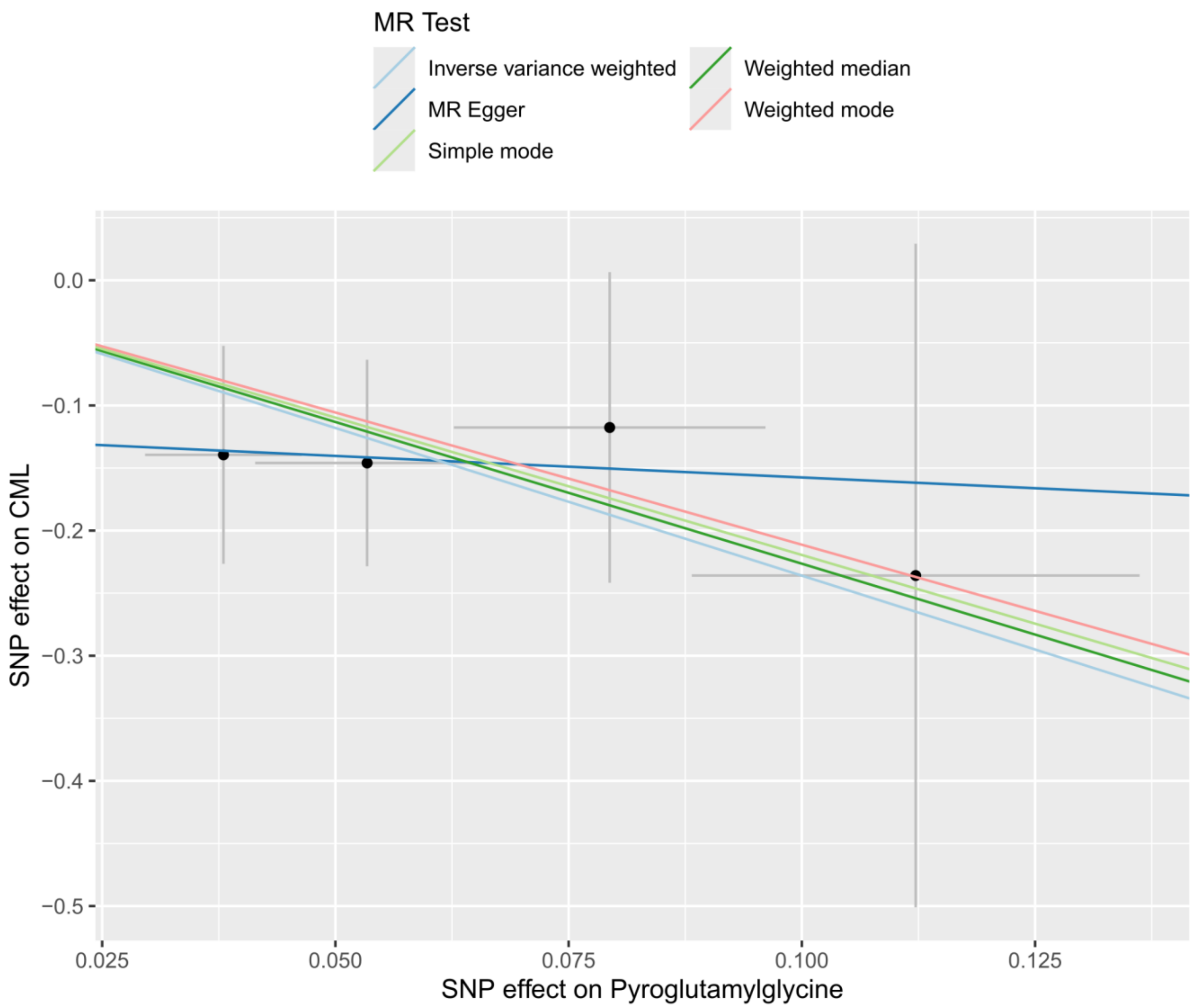
MR Test
Inverse variance weighted
MR Egger
Simple mode
Weighted median
Weighted mode
0.0
−0.1
−0.2
−0.3
−0.4
−0.5
SNP effect on CML
0.025
0.050
0.075
0.100
0.125
SNP effect on Pyroglutamylglycine

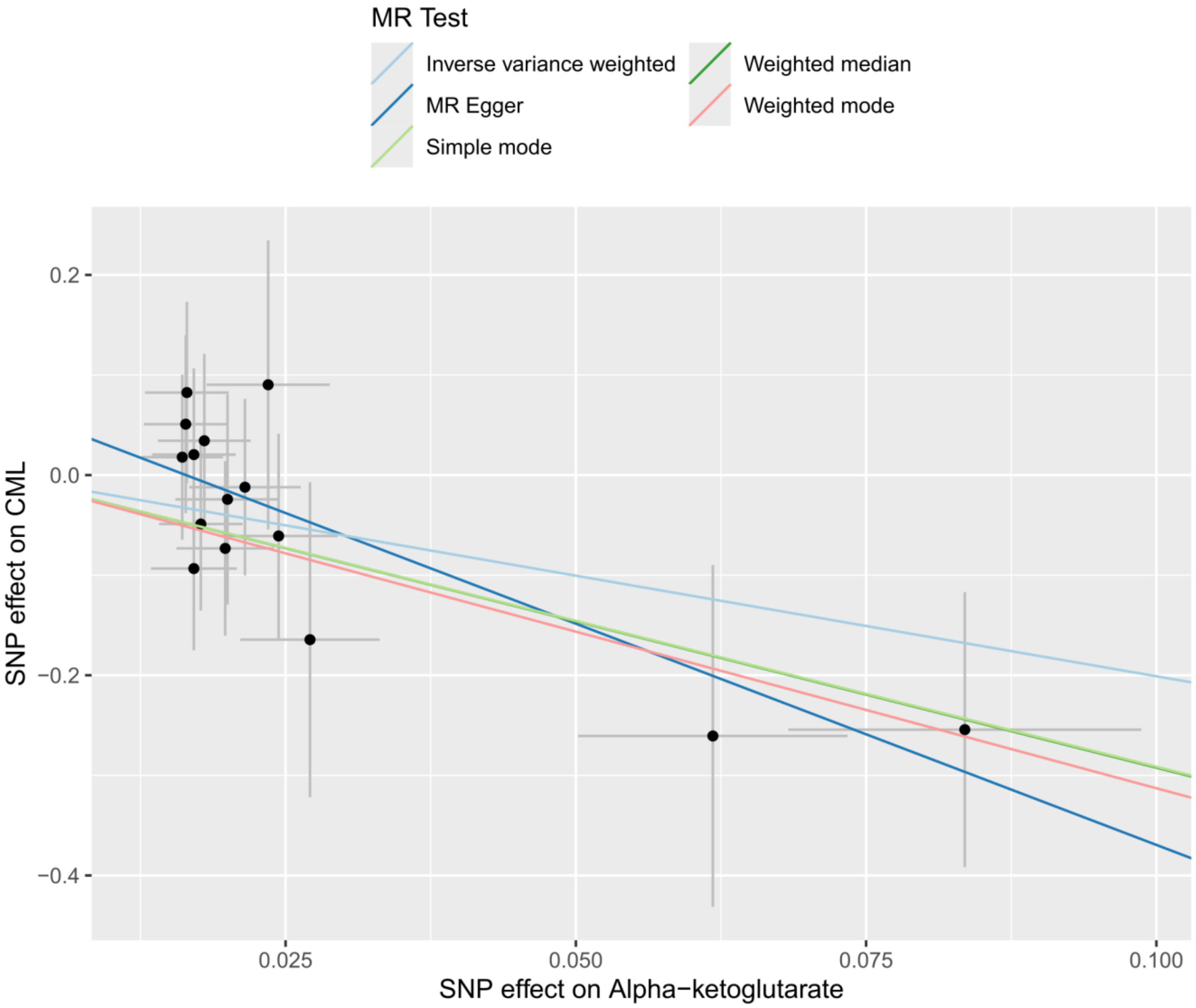
MR Test
Inverse variance weighted
MR Egger
Simple mode
Weighted median
Weighted mode
0.2
0.0
−0.2
−0.4
SNP effect on CML
0.025
0.050
0.075
0.100
SNP effect on Alpha−ketoglutarate

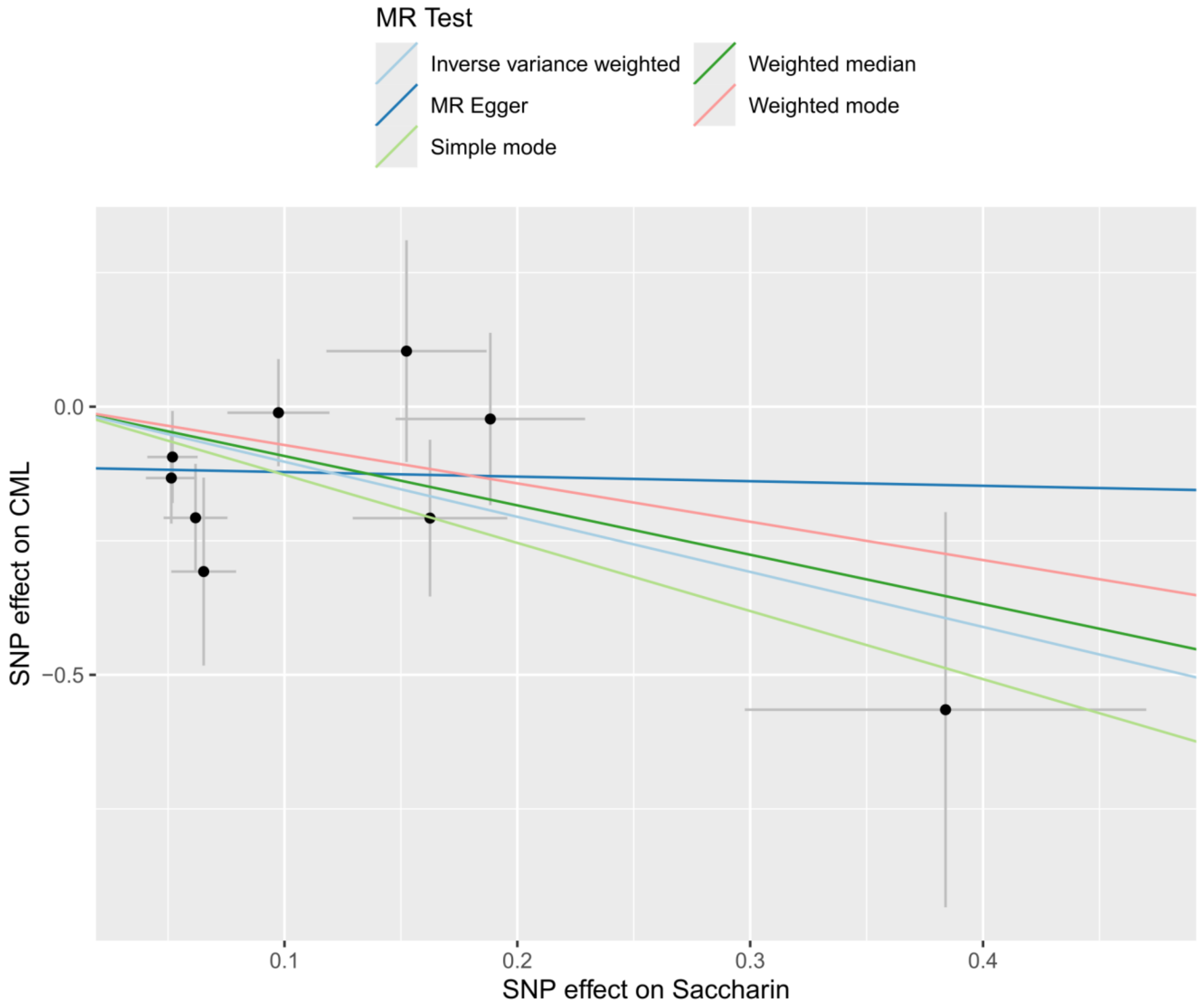
MR Test
Inverse variance weighted
MR Egger
Simple mode
Weighted median
Weighted mode
0.0
−0.5
SNP effect on CML
0.1
0.2
0.3
0.4
SNP effect on Saccharin

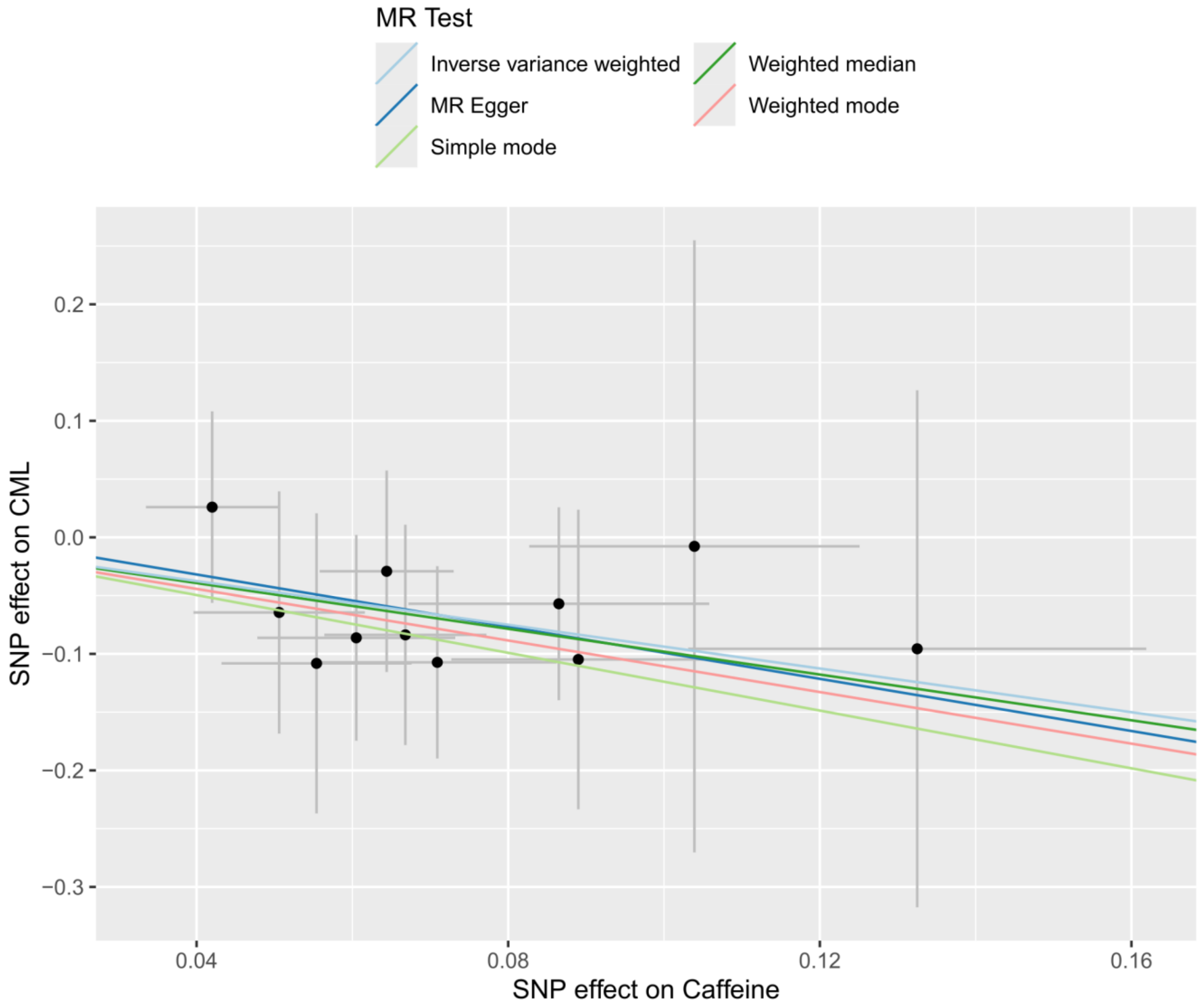
MR Test
Inverse variance weighted
MR Egger
Simple mode
Weighted median
Weighted mode
SNP effect on CML
0.2
0.1
0.0
−0.1
−0.2
−0.3
0.04
0.08
0.12
0.16
SNP effect on Caffeine

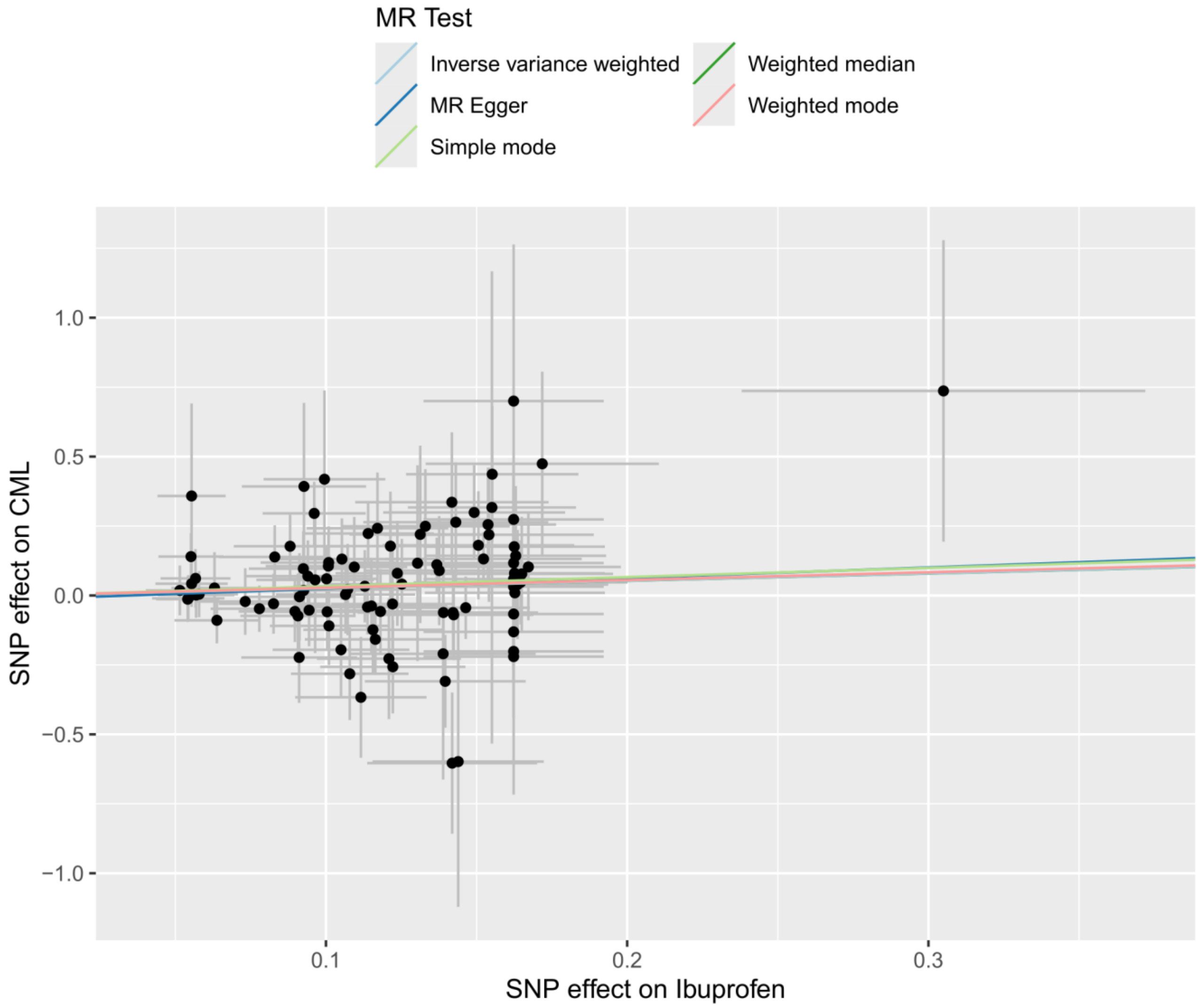
MR Test
Inverse variance weighted
MR Egger
Simple mode
Weighted median
Weighted mode
1.0
0.5
0.0
−0.5
−1.0
SNP effect on CML
0.1
0.2
0.3
SNP effect on Ibuprofen

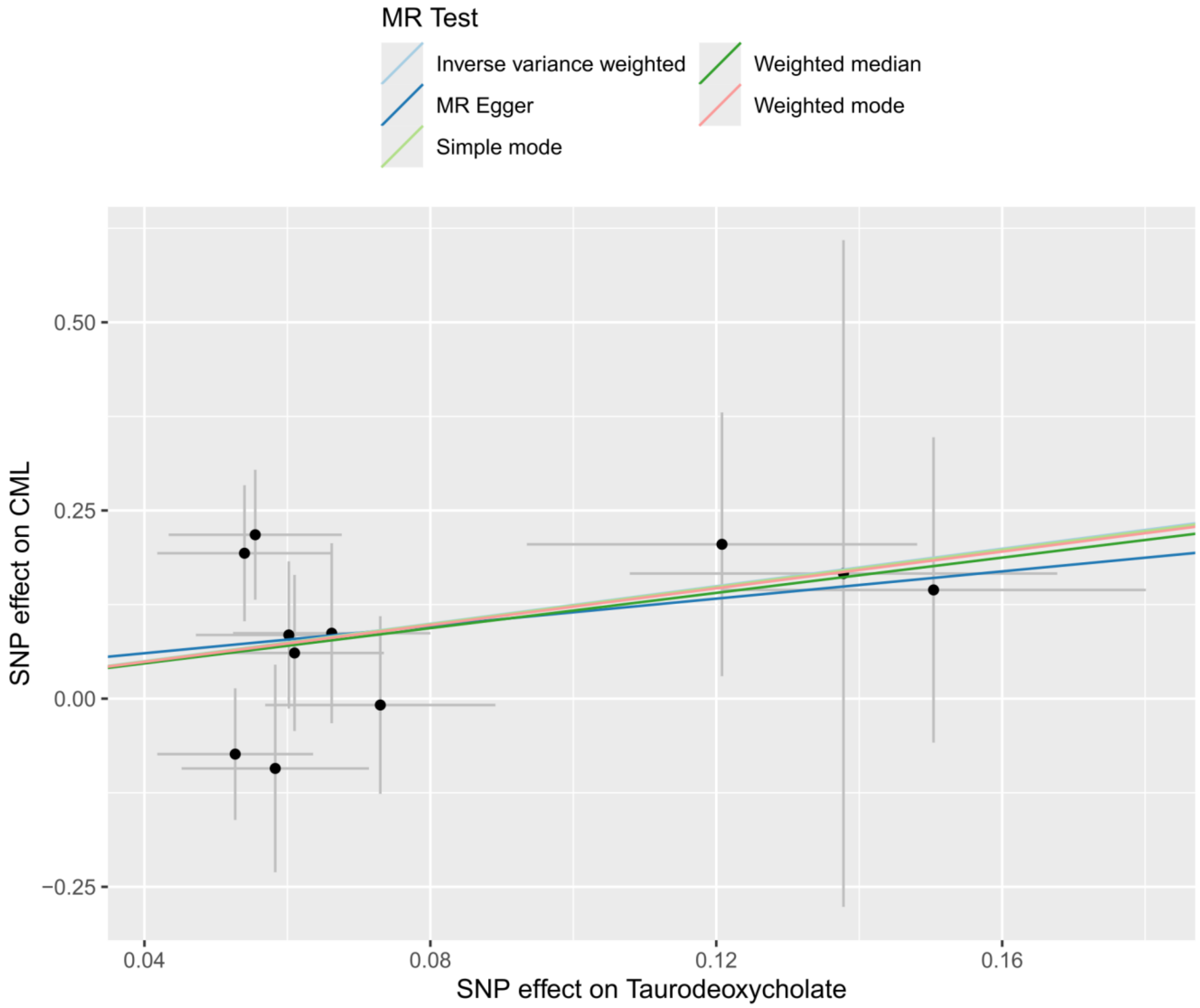
MR Test
Inverse variance weighted
MR Egger
Simple mode
Weighted median
Weighted mode
0.50
0.25
0.00
−0.25
SNP effect on CML
0.04
0.08
0.12
0.16
SNP effect on Taurodeoxycholate

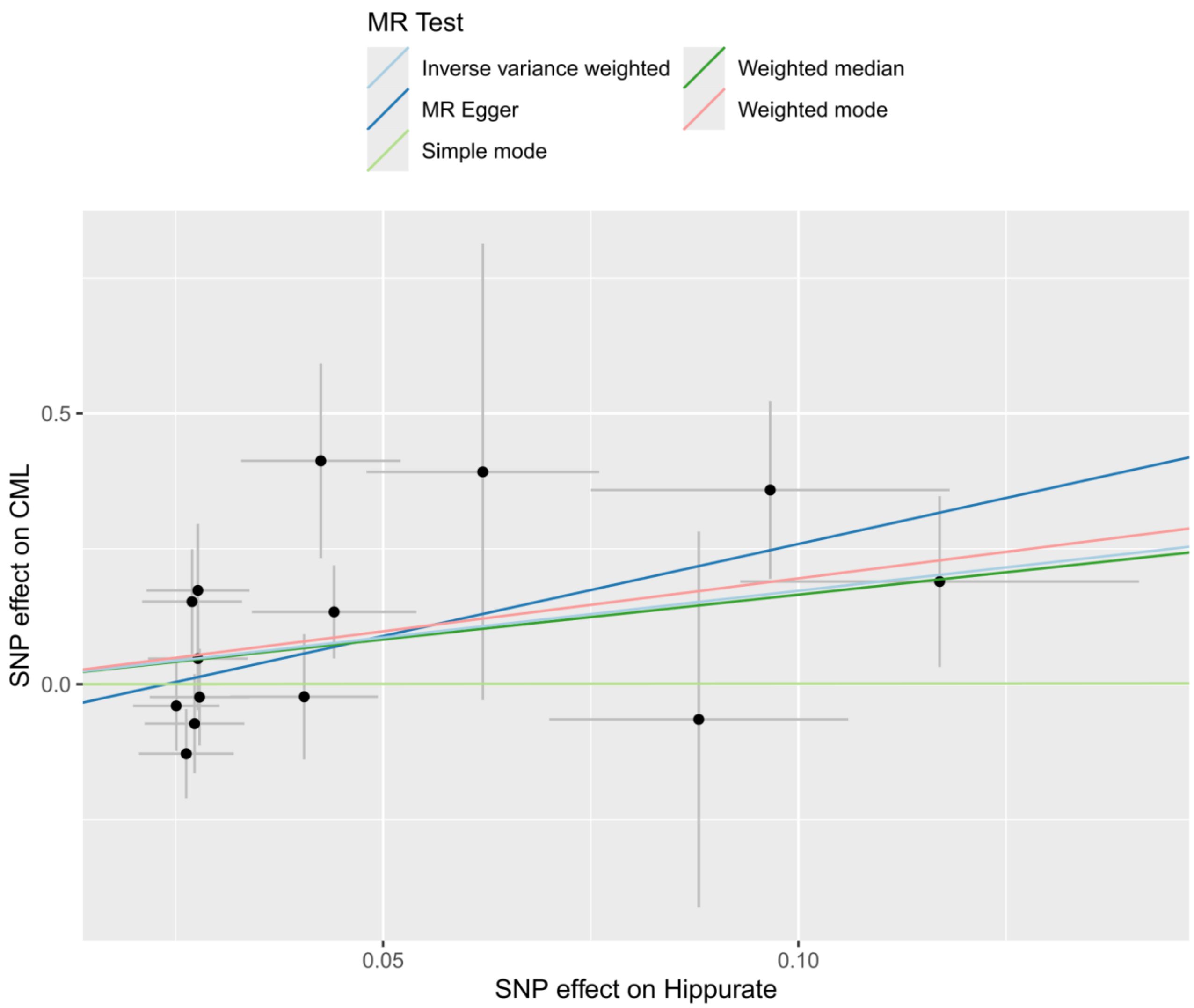
MR Test
Inverse variance weighted
MR Egger
Simple mode
Weighted median
Weighted mode
0.5
0.0
0.05
0.10
SNP effect on CML
SNP effect on Hippurate

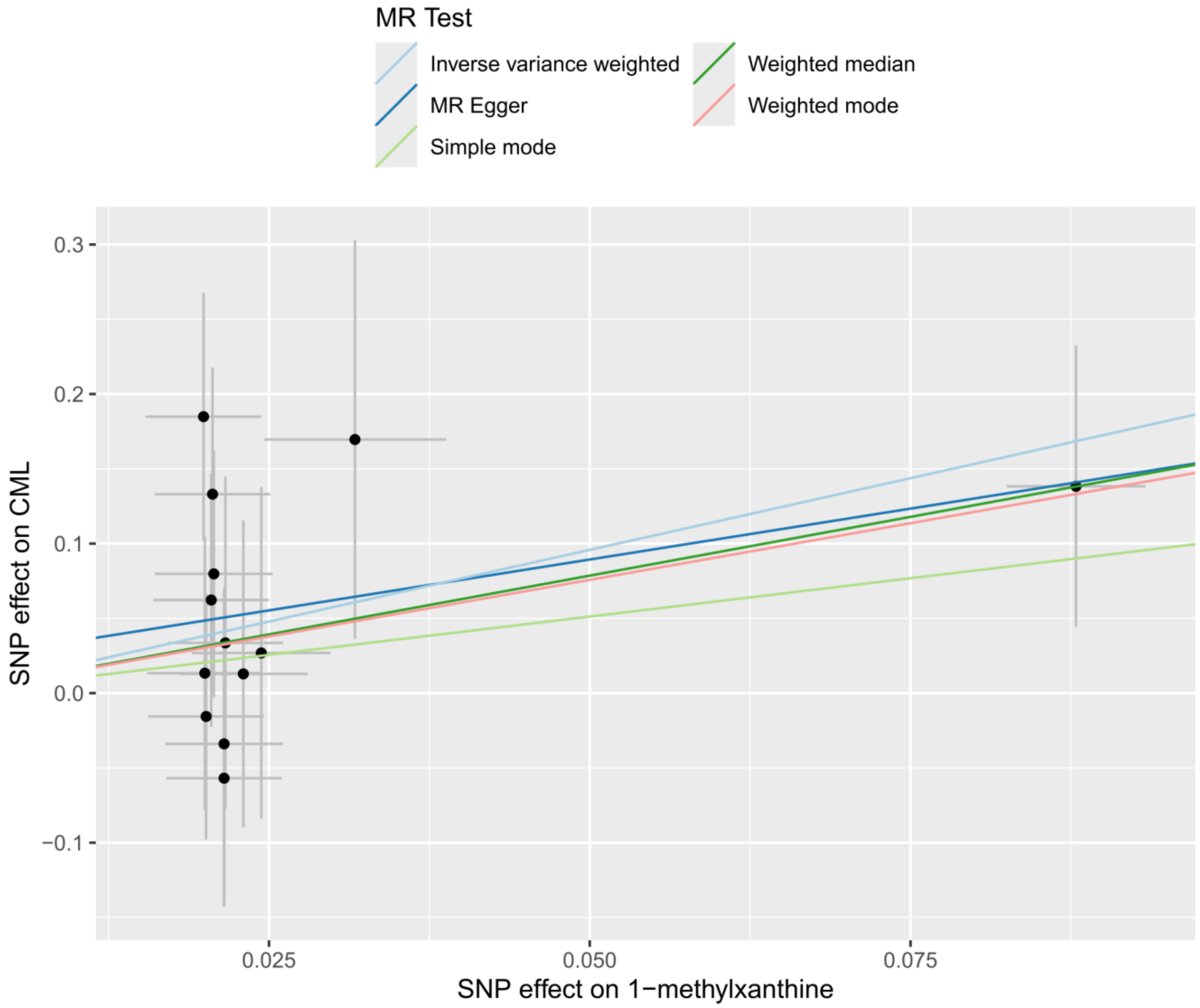

MR Test
Inverse variance weighted
MR Egger
Simple mode
Weighted median
Weighted mode
0.3
0.2
0.1
0.0
−0.1
SNP effect on CML
0.025
0.050
0.075
SNP effect on 1−methylxanthine

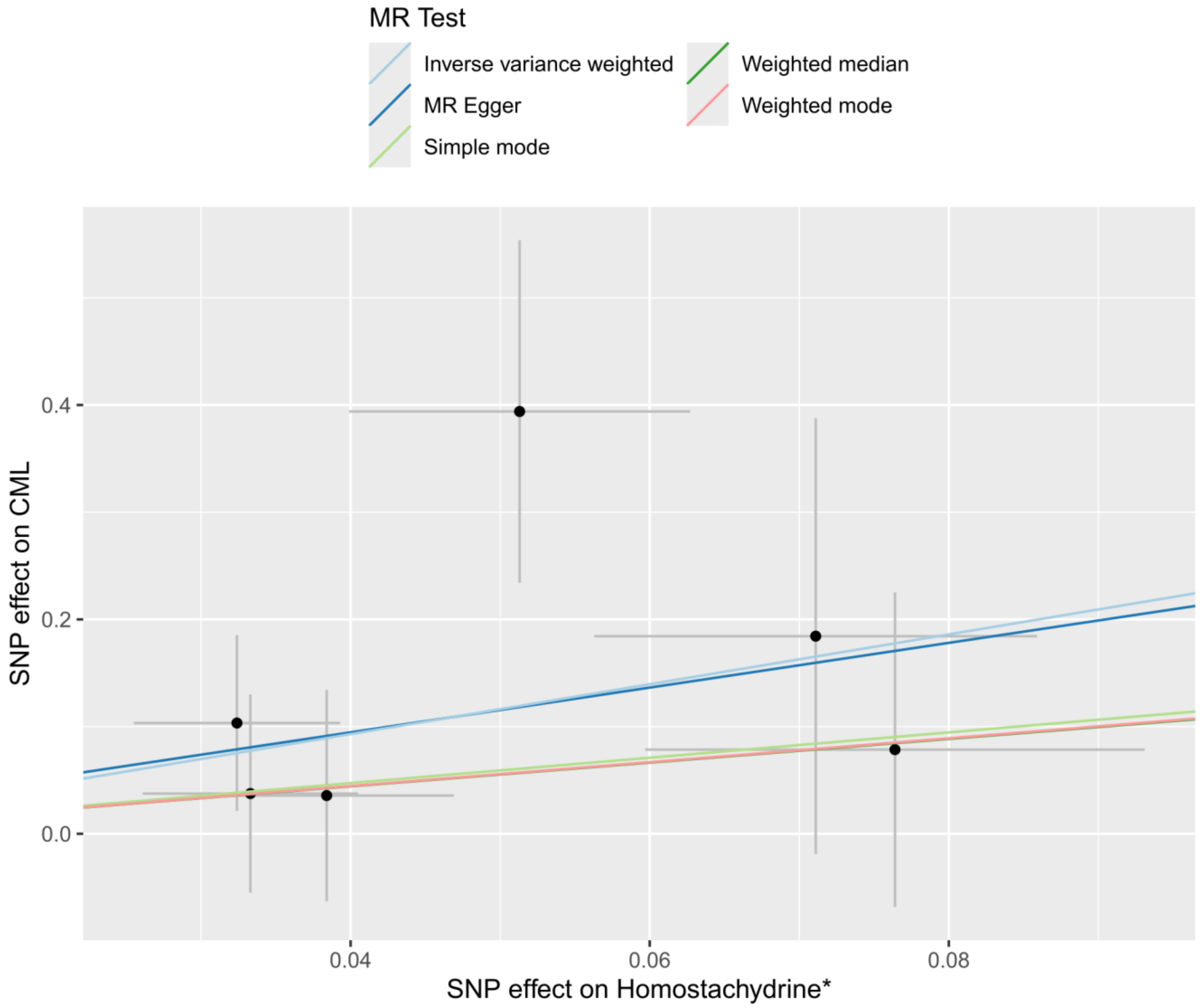
MR Test
Inverse variance weighted
MR Egger
Simple mode
Weighted median
Weighted mode
SNP effect on CML
0.4
0.2
0.0
0.04
0.06
0.08
SNP effect on Homostachydrine*

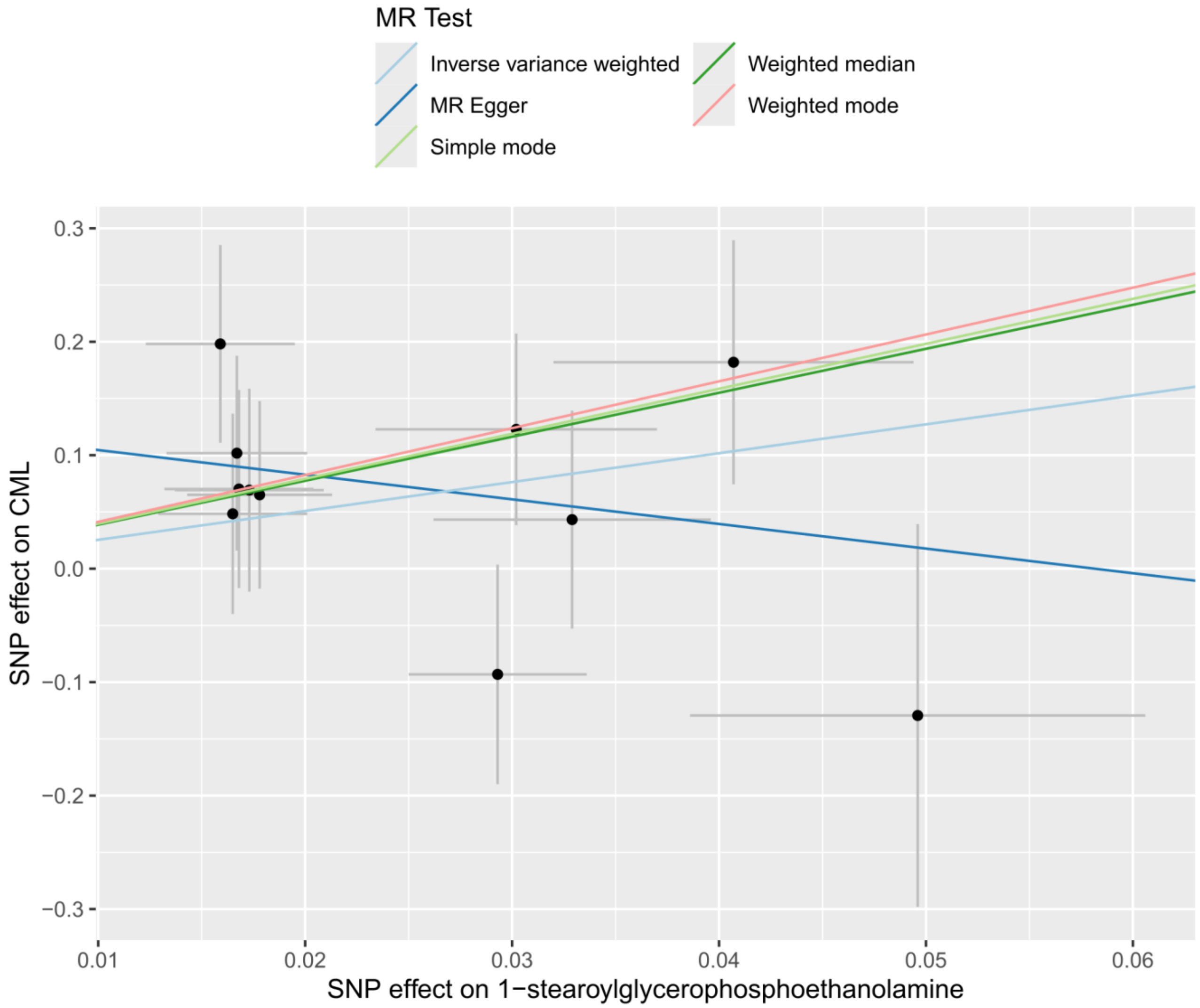
MR Test
Inverse variance weighted
MR Egger
Simple mode
Weighted median
Weighted mode
0.3
0.2
0.1
0.0
−0.1
−0.2
−0.3
SNP effect on CML
0.01
0.02
0.03
0.04
0.05
0.06
SNP effect on 1−stearoylglycerophosphoethanolamine

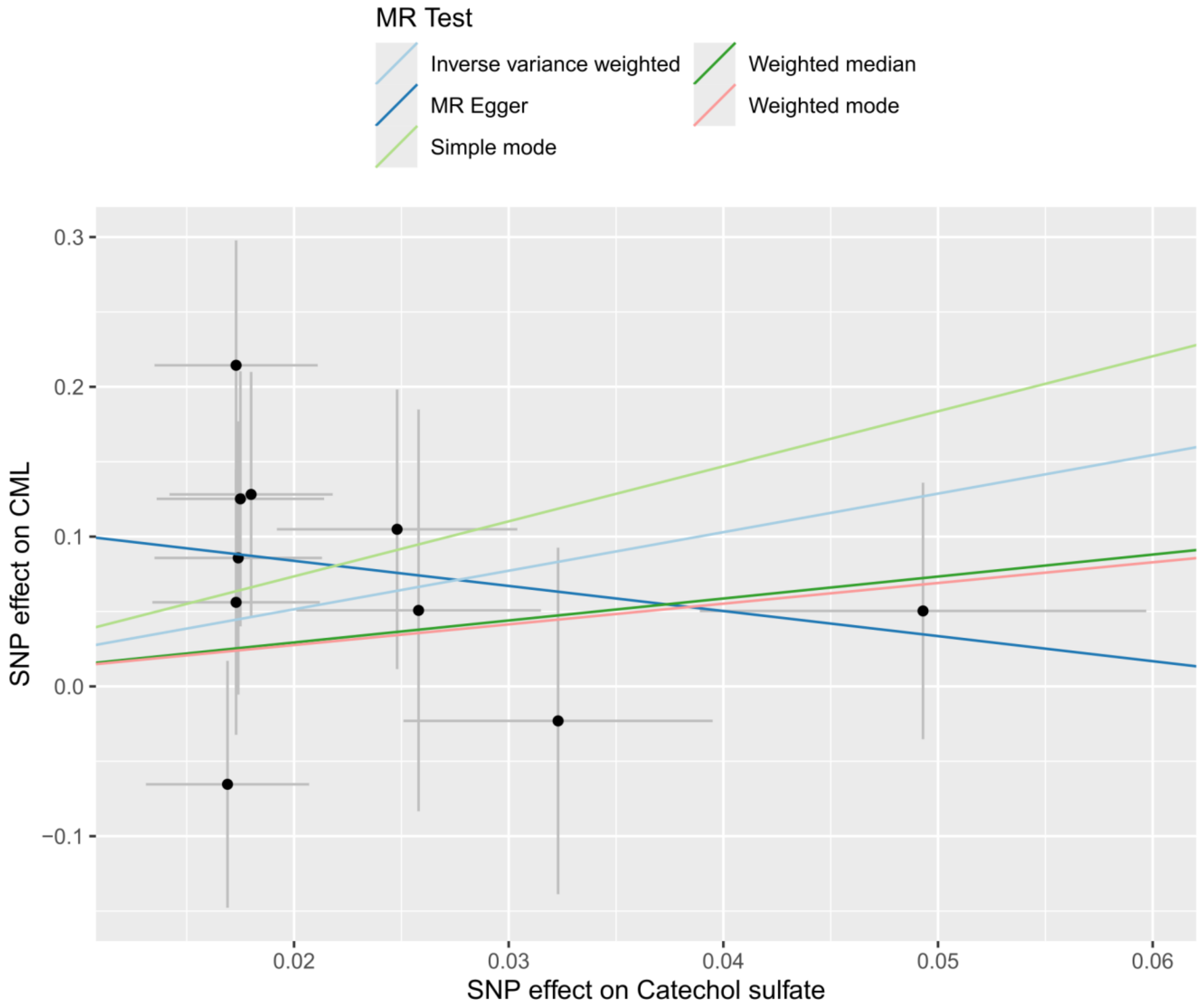

MR Test
Inverse variance weighted
MR Egger
Simple mode
Weighted median
Weighted mode
0.3
0.2
0.1
0.0
−0.1
SNP effect on CML
0.02
0.03
0.04
0.05
0.06
SNP effect on Catechol sulfate

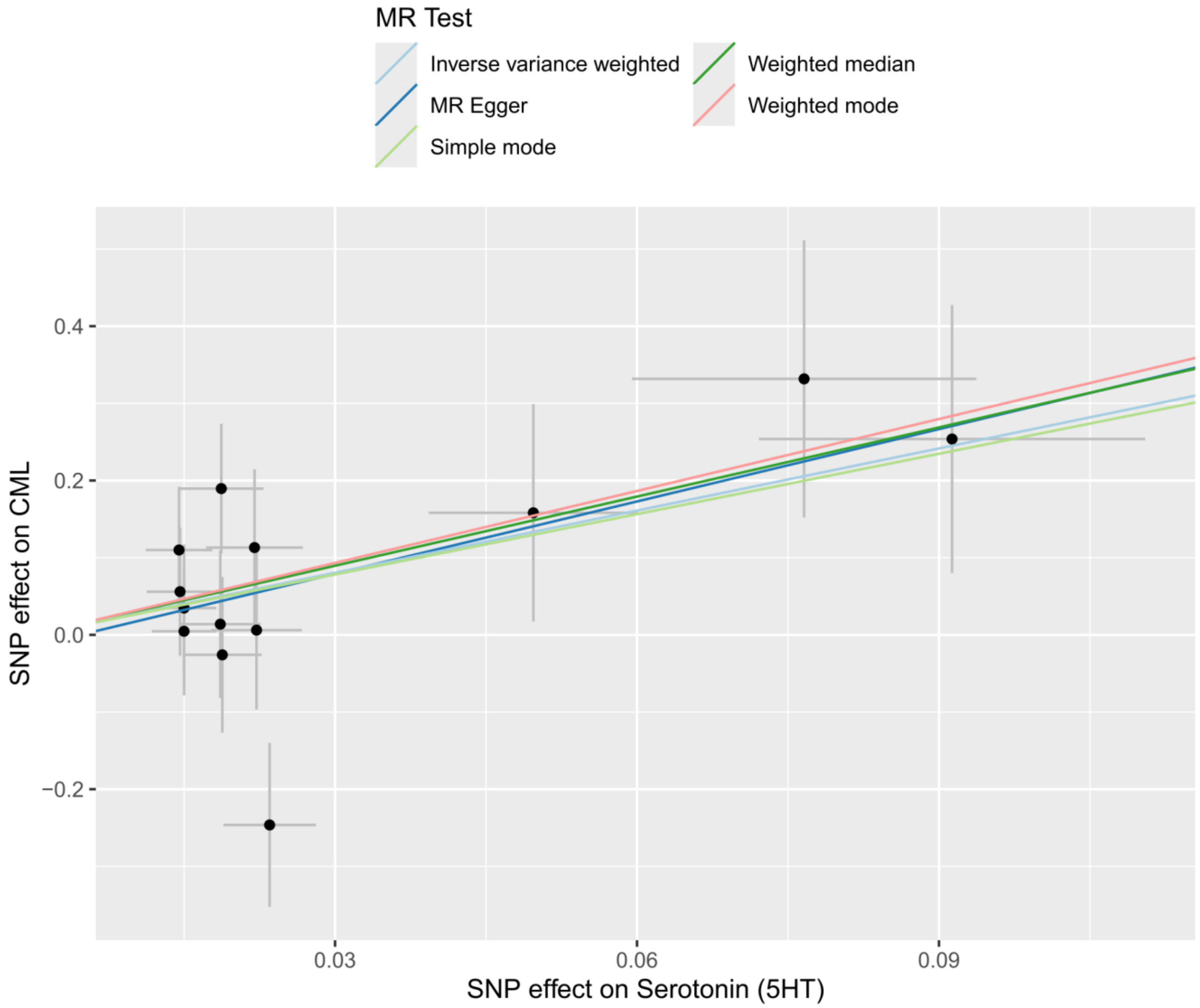
MR Test
Inverse variance weighted
MR Egger
Simple mode
Weighted median
Weighted mode
SNP effect on CML
0.4
0.2
0.0
−0.2
0.03
0.06
0.09
SNP effect on Serotonin (5HT)

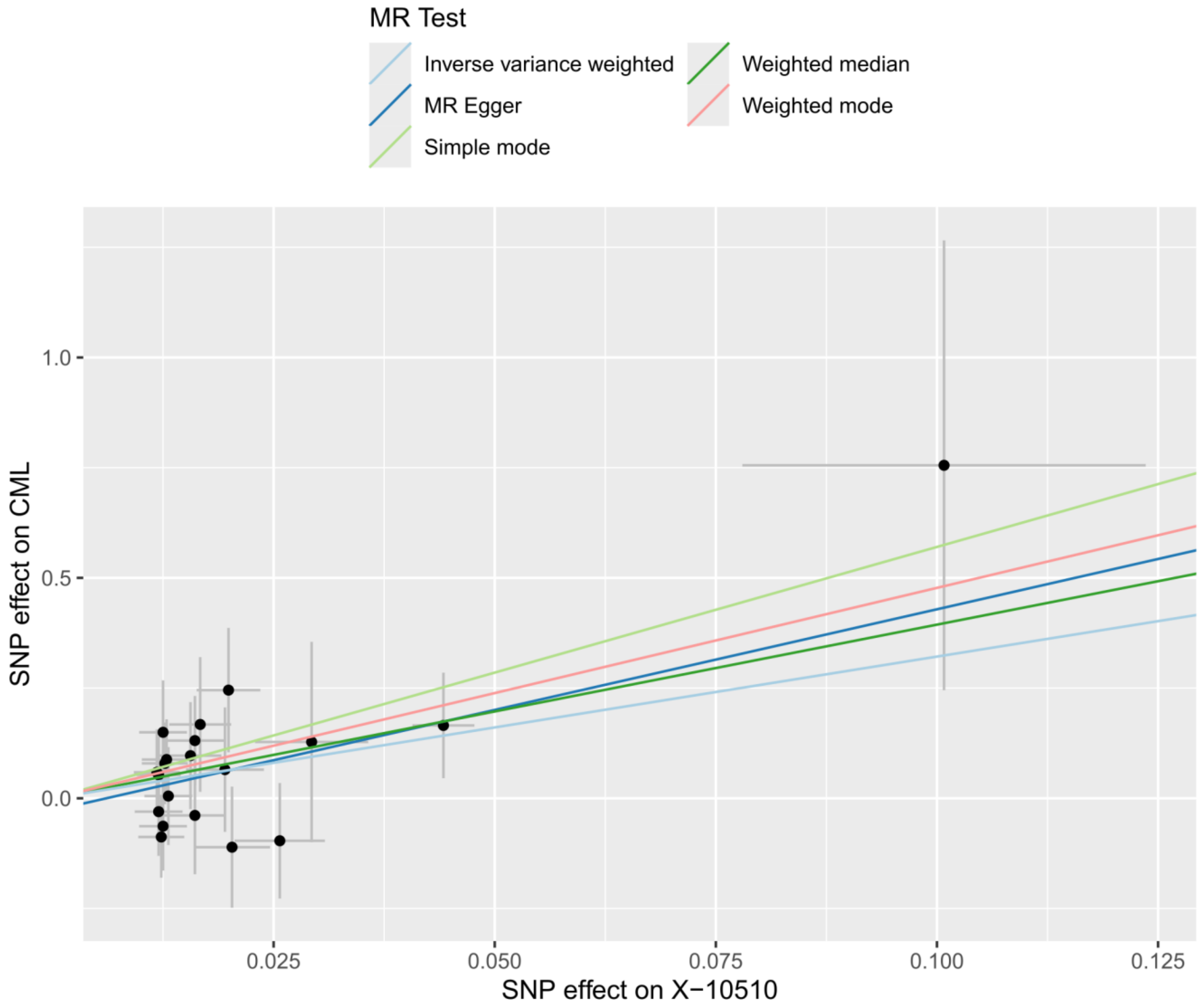
MR Test
Inverse variance weighted
MR Egger
Simple mode
Weighted median
Weighted mode
1.0
0.5
0.0
SNP effect on CML
0.025
0.050
0.075
0.100
0.125
SNP effect on X−10510

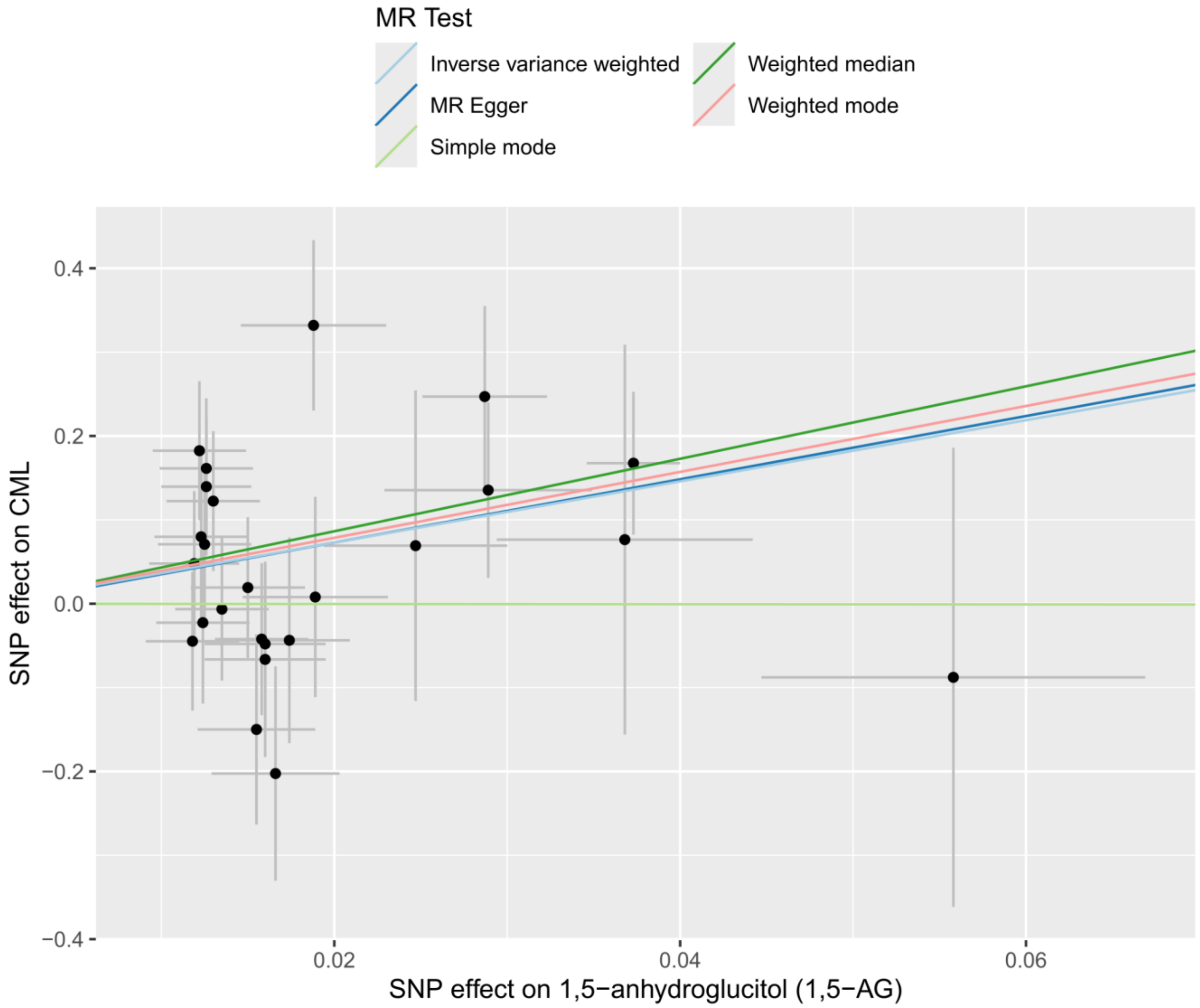

MR Test
Inverse variance weighted
MR Egger
Simple mode
Weighted median
Weighted mode
0.4
0.2
0.0
−0.2
−0.4
SNP effect on CML
0.02
0.04
0.06
SNP effect on 1,5−anhydroglucitol (1,5−AG)

Supplementary Figure S2. Funnel plots for 20 potential metabolites on CML.

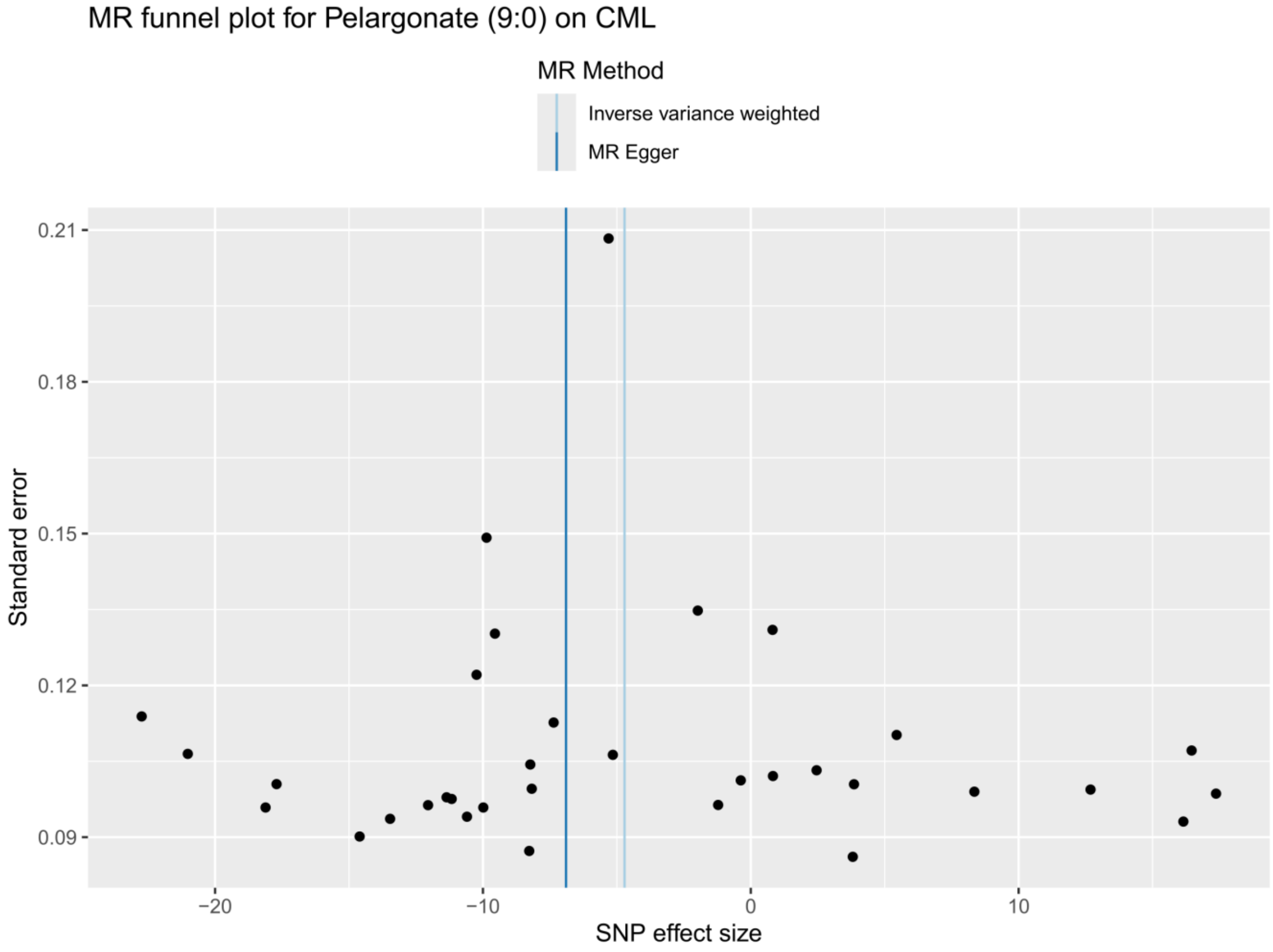

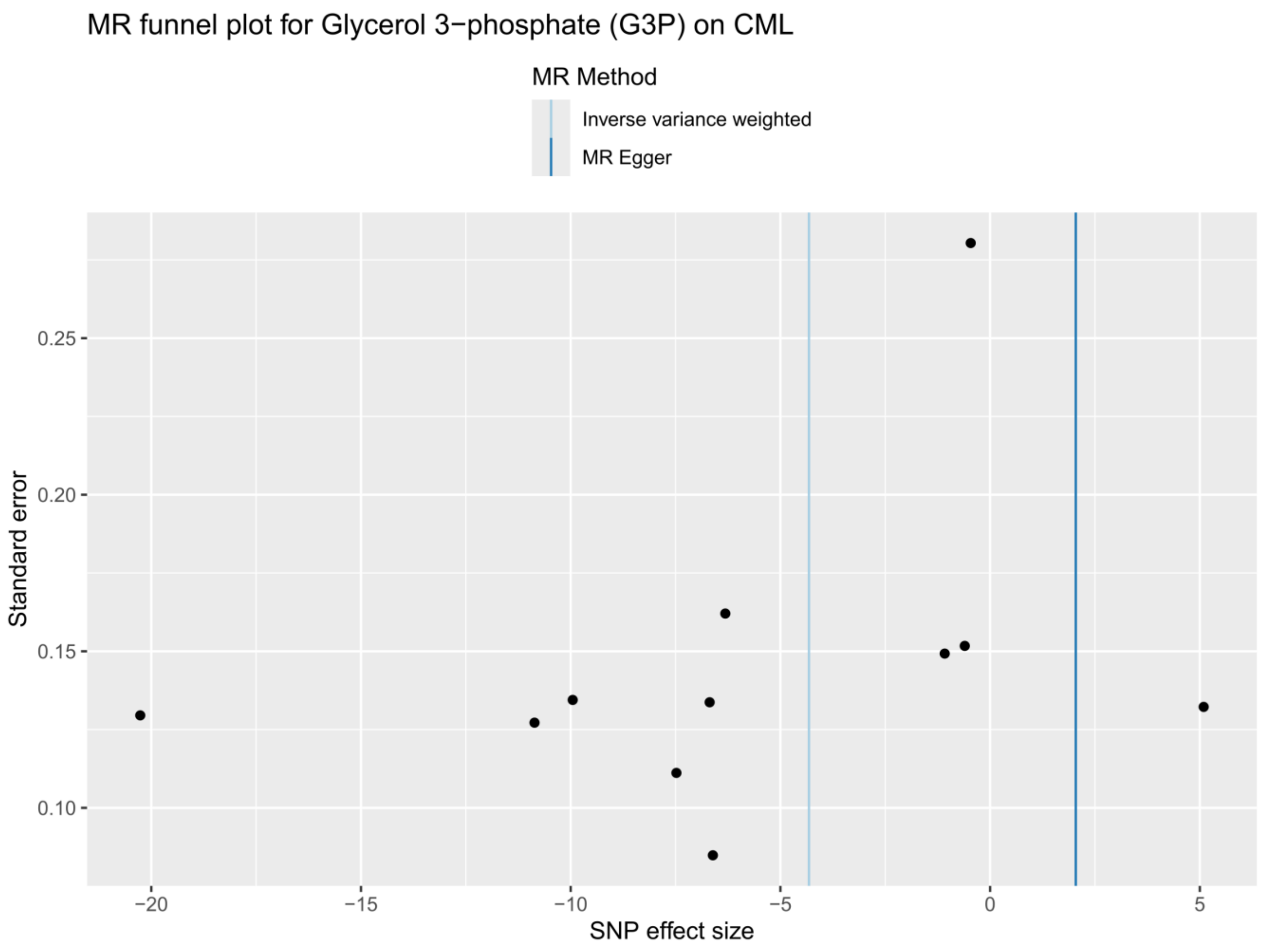
MR funnel plot for Glycerol 3−phosphate (G3P) on CML
MR Method
Inverse variance weighted
MR Egger
Standard error
0.25
0.20
0.15
0.10
−20
−15
−10
−5
0
5
SNP effect size

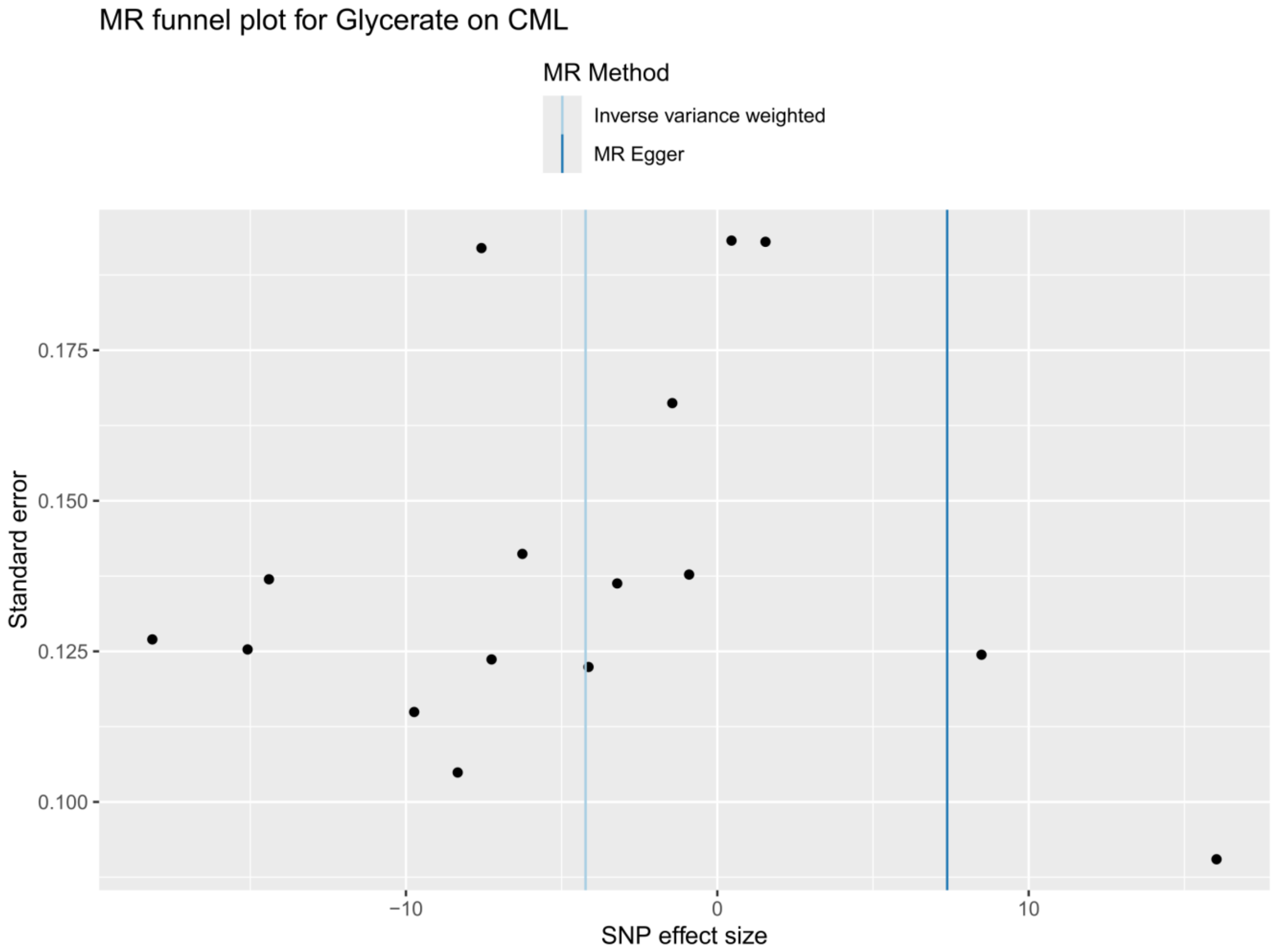

MR funnel plot for Glycerate on CML
MR Method
Inverse variance weighted
MR Egger
Standard error
0.175
0.150
0.125
0.100
−10
0
10
SNP effect size

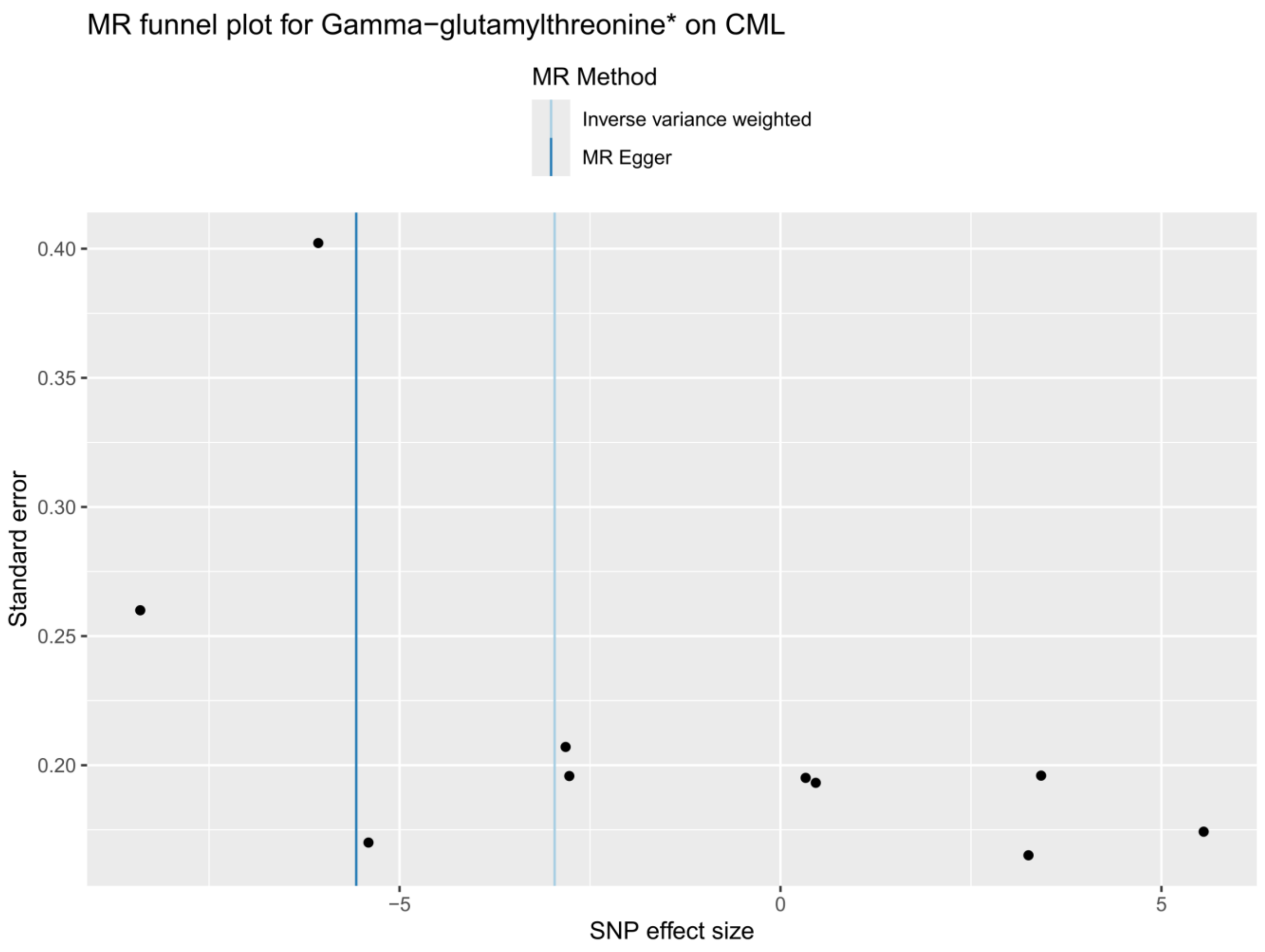

MR funnel plot for Gamma−glutamylthreonine* on CML
MR Method
Inverse variance weighted
MR Egger
Standard error
0.40
0.35
0.30
0.25
0.20
−5
0
5
SNP effect size

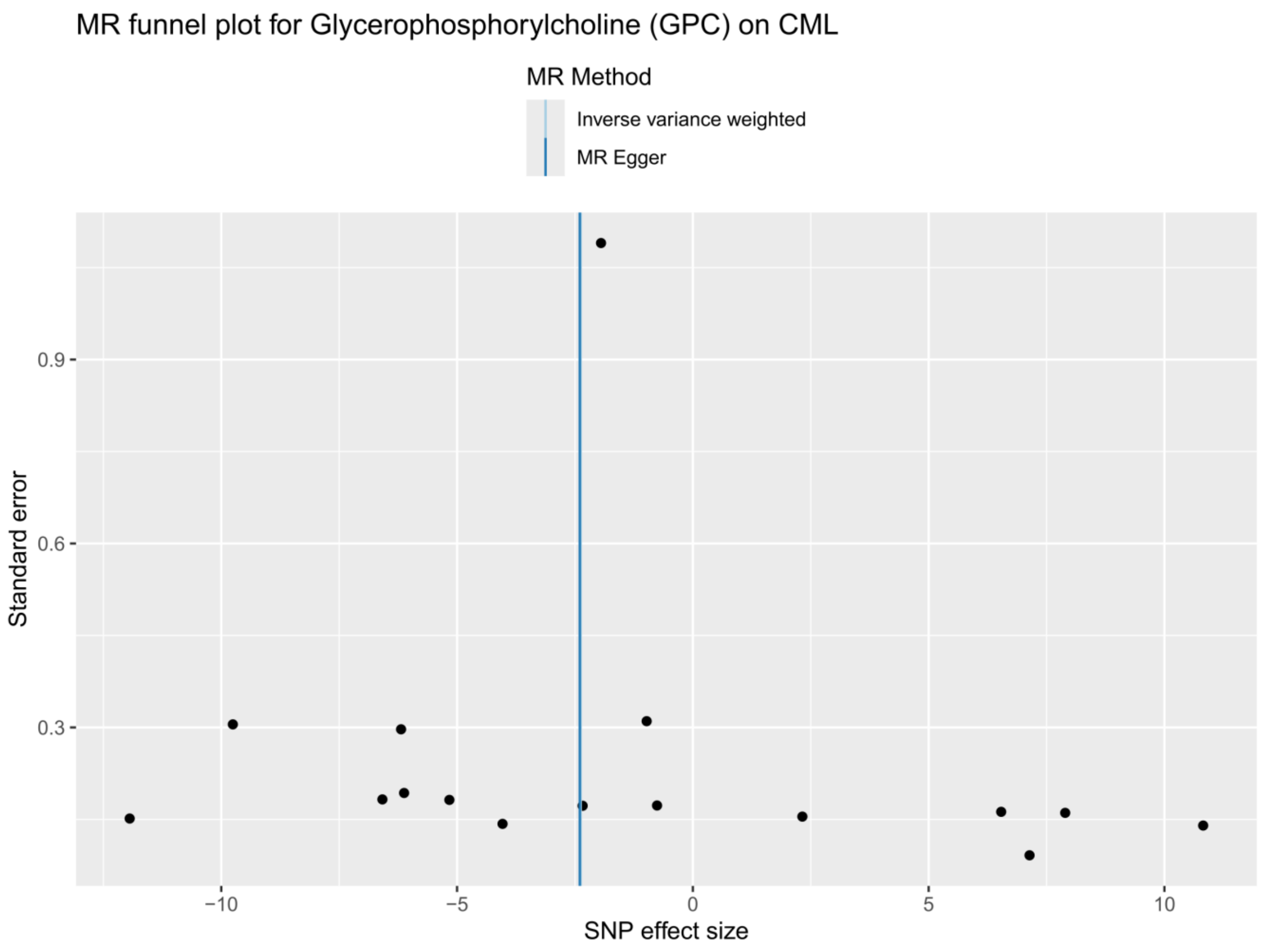
MR funnel plot for Glycerophosphorylcholine (GPC) on CML
MR Method
Inverse variance weighted
MR Egger
Standard error
0.9
0.6
0.3
−10
−5
0
5
10
SNP effect size

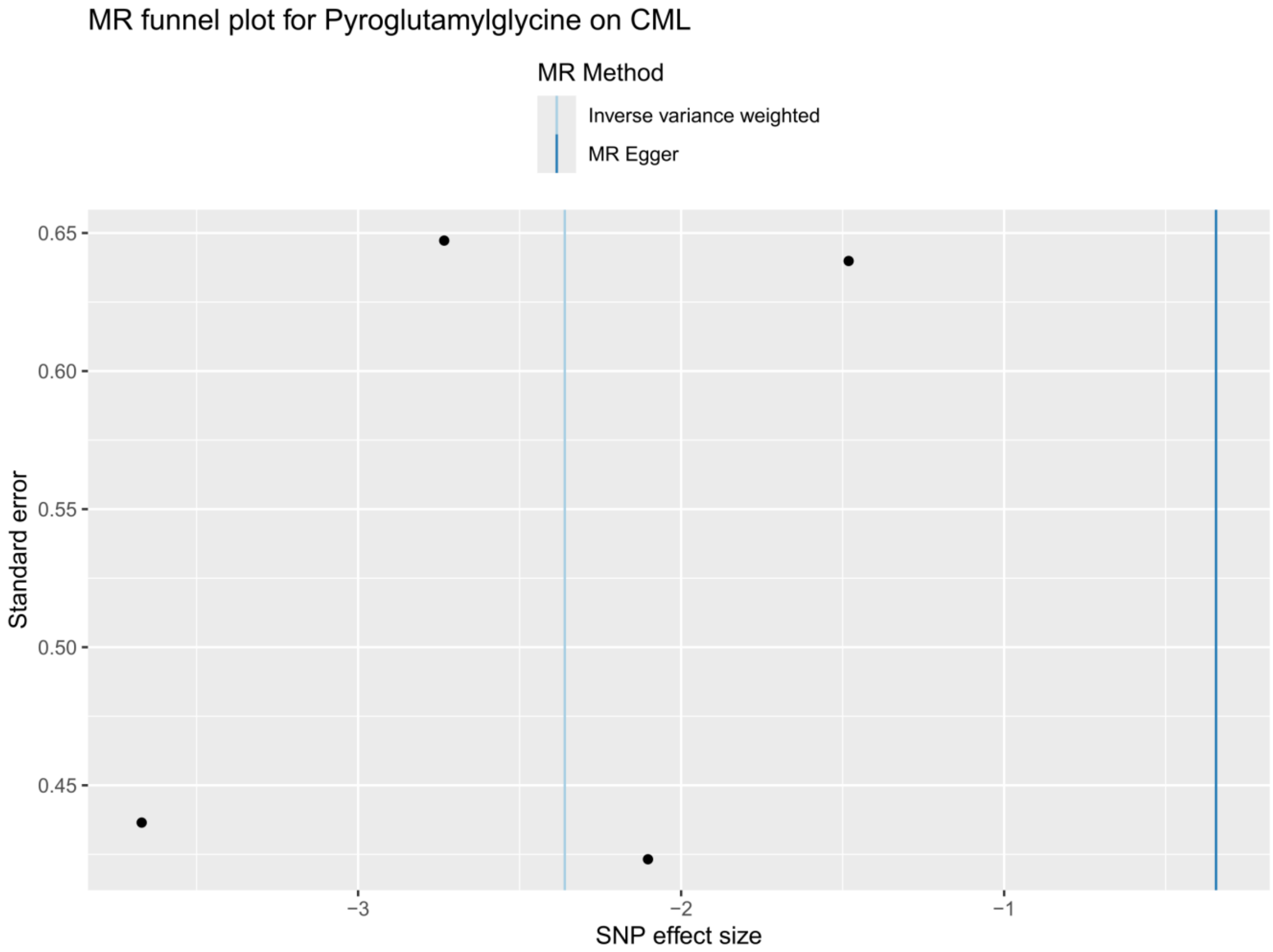

MR funnel plot for Pyroglutamylglycine on CML
MR Method
Inverse variance weighted
MR Egger
0.65
0.60
0.55
0.50
0.45
Standard error
−3
−2
−1
SNP effect size

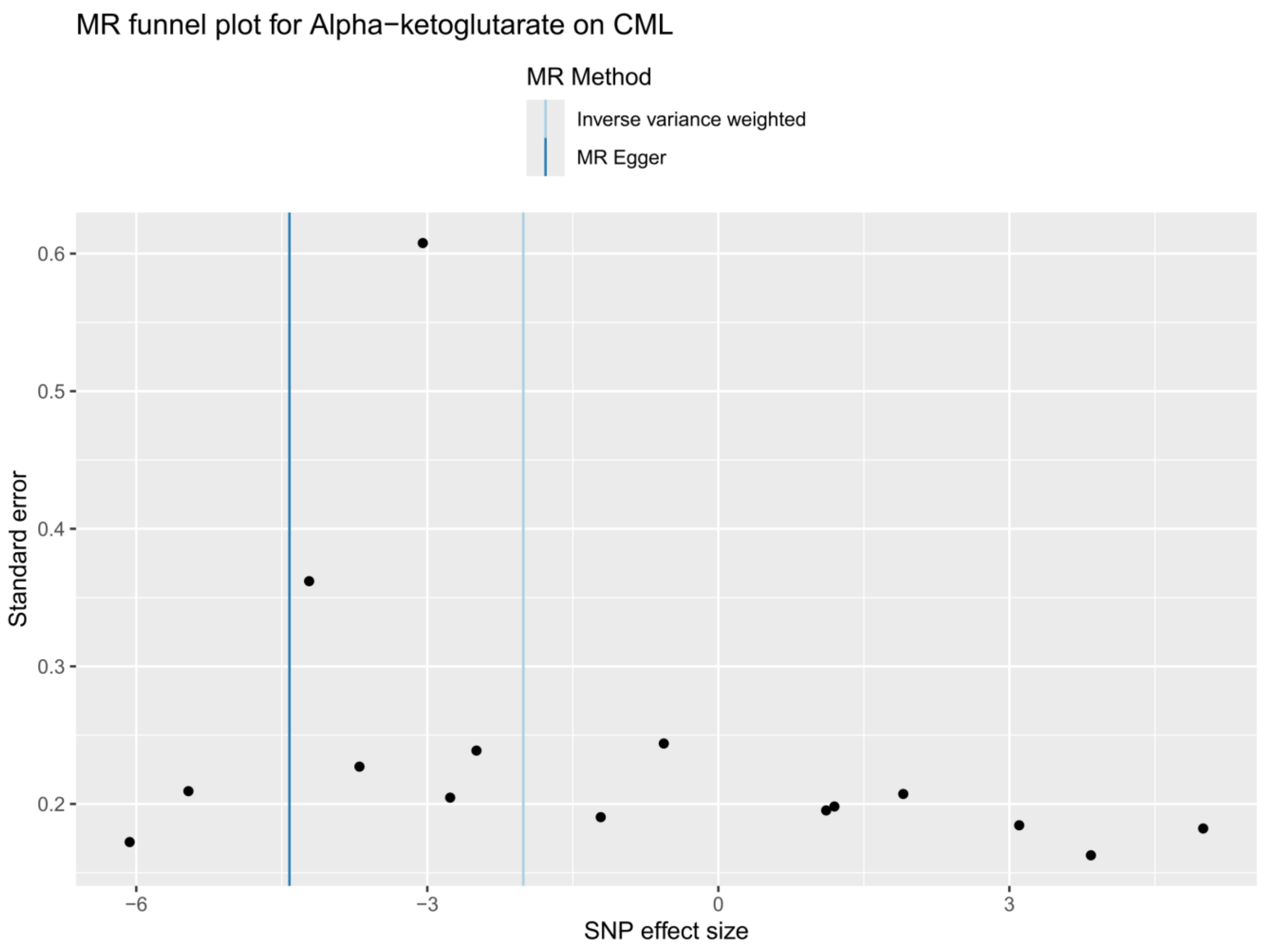
MR funnel plot for Alpha−ketoglutarate on CML
MR Method
Inverse variance weighted
MR Egger
Standard error
0.6
0.5
0.4
0.3
0.2
−6
−3
0
3
SNP effect size

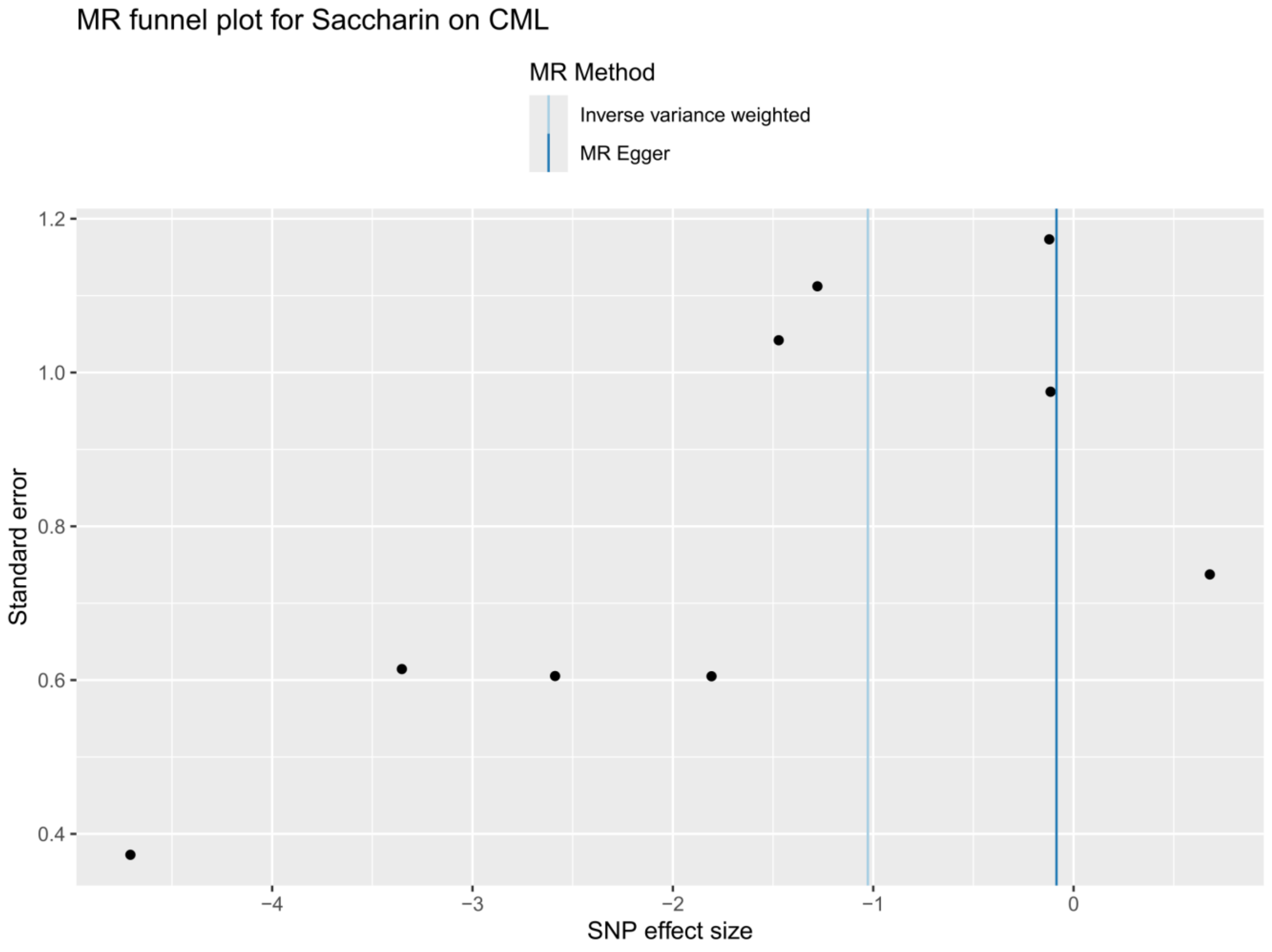
MR funnel plot for Saccharin on CML
MR Method
Inverse variance weighted
MR Egger
Standard error
1.2
1.0
0.8
0.6
0.4
−4
−3
−2
−1
0
SNP effect size

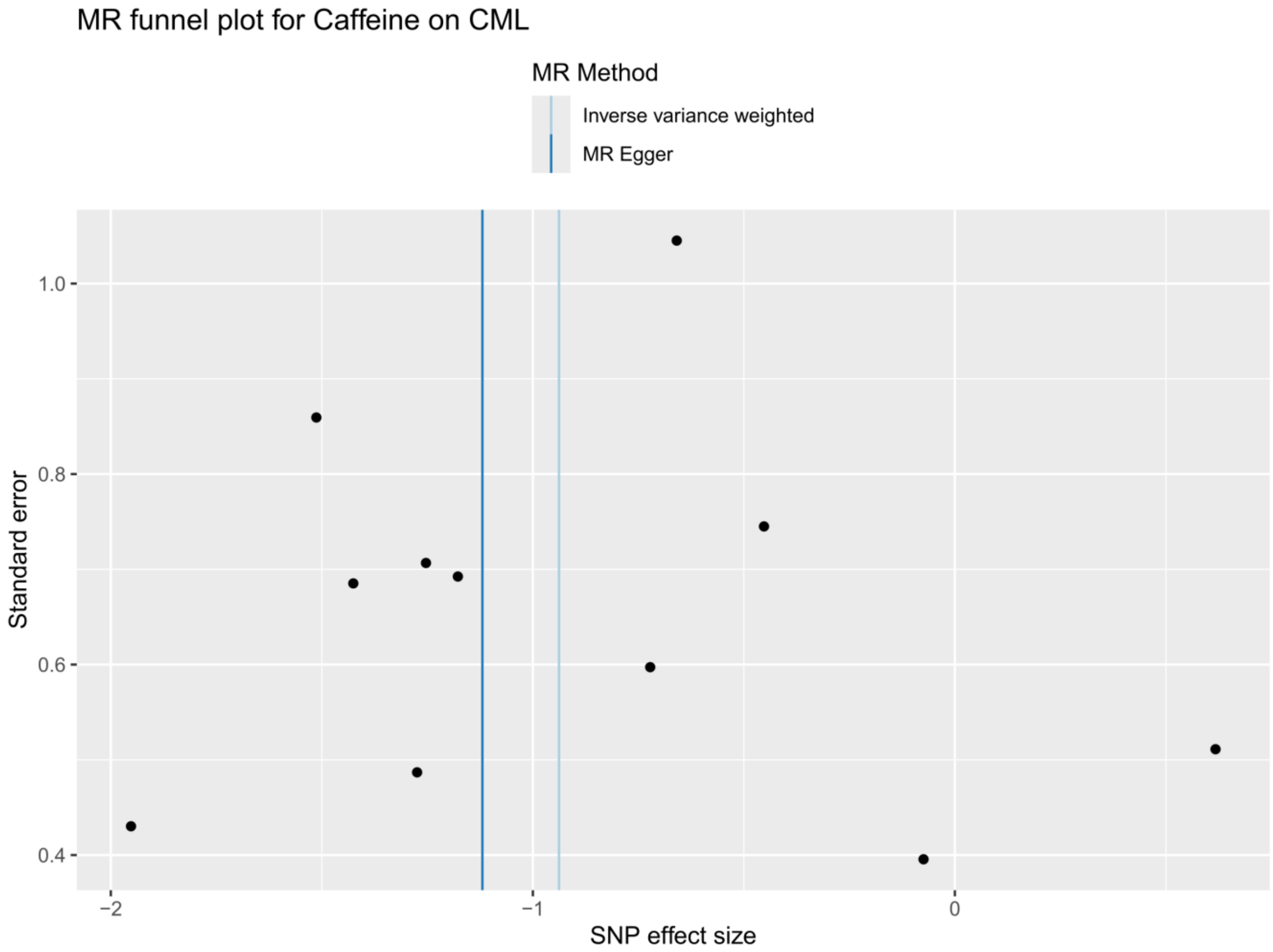
MR funnel plot for Caffeine on CML
MR Method
Inverse variance weighted
MR Egger
Standard error
1.0
0.8
0.6
0.4
−2
−1
0
SNP effect size

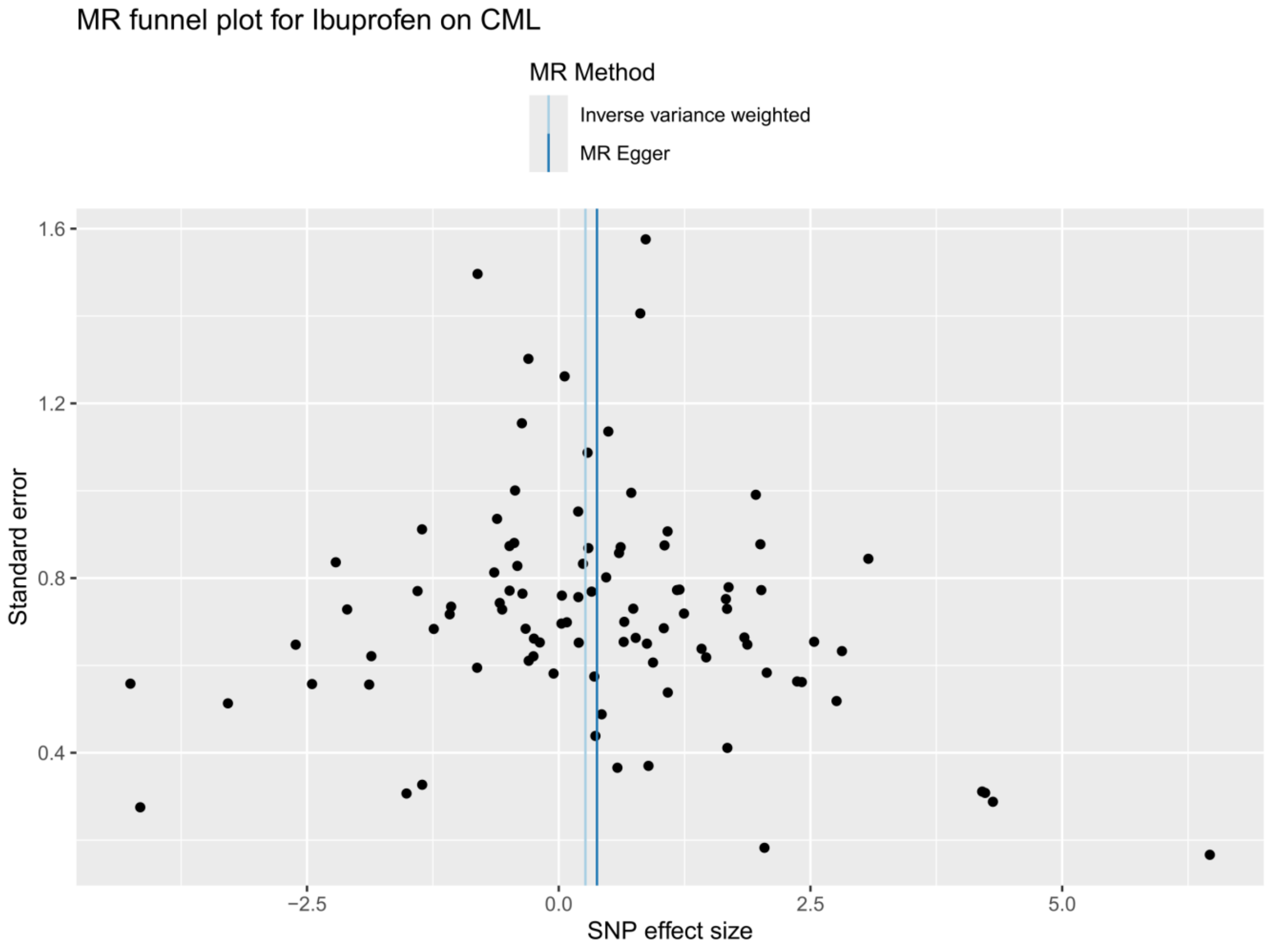
MR funnel plot for Ibuprofen on CML
MR Method
Inverse variance weighted
MR Egger
Standard error
1.6
1.2
0.8
0.4
−2.5
0.0
2.5
5.0
SNP effect size

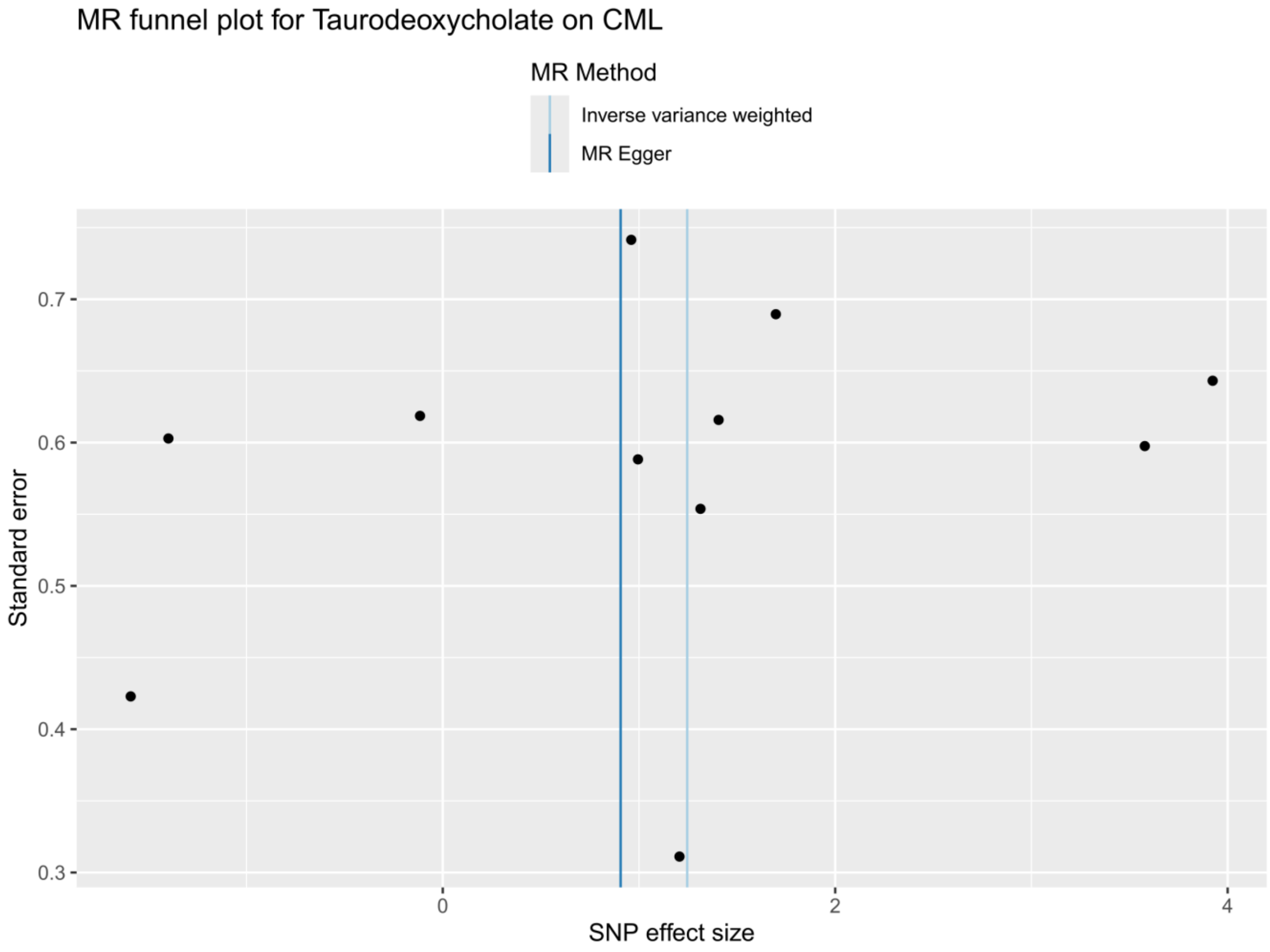
MR funnel plot for Taurodeoxycholate on CML
MR Method
Inverse variance weighted
MR Egger
Standard error
0.7
0.6
0.5
0.4
0.3
0
2
4
SNP effect size

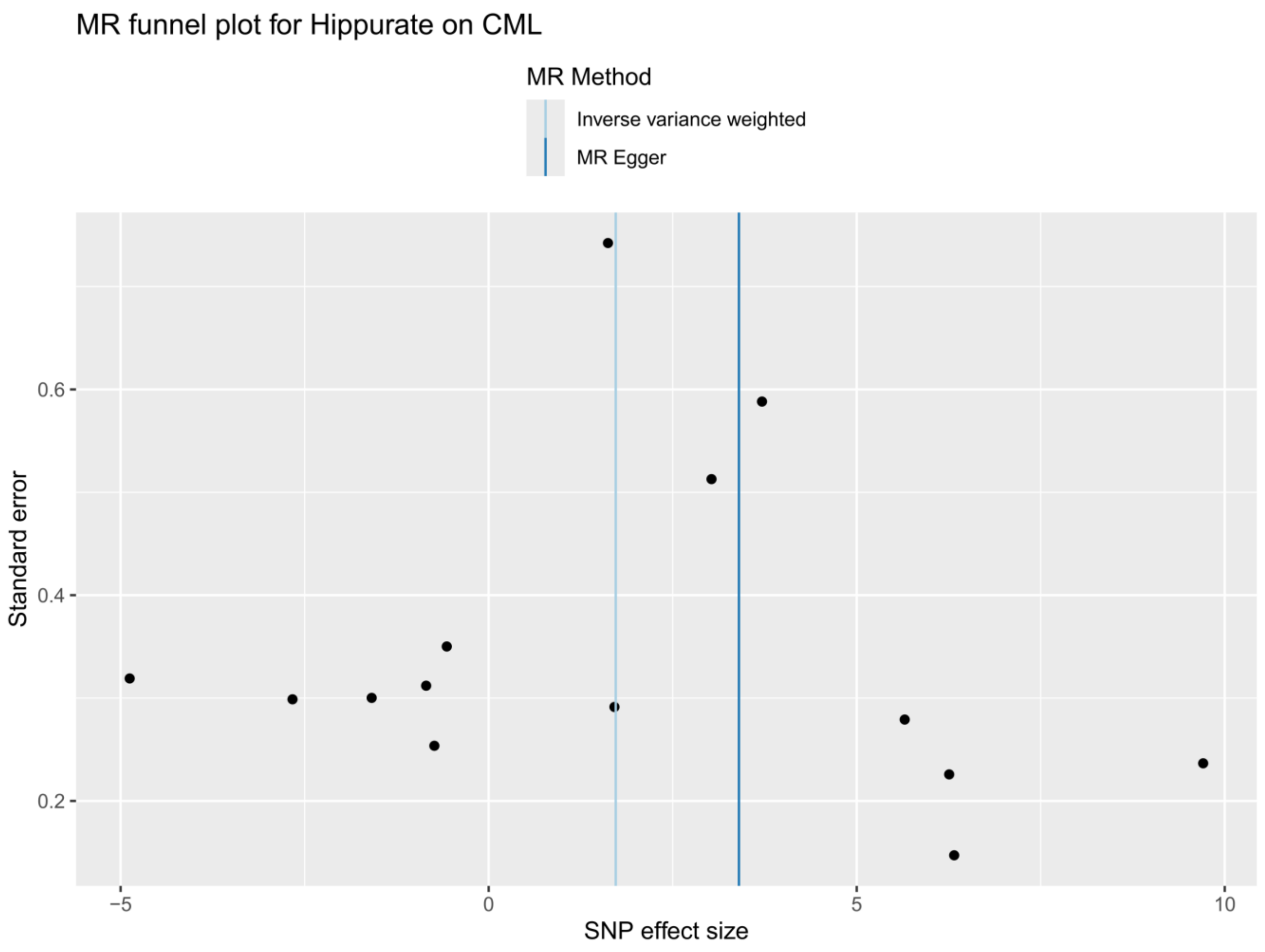
MR funnel plot for Hippurate on CML
MR Method
Inverse variance weighted
MR Egger
0.6
0.4
0.2
Standard error
−5
0
5
10
SNP effect size

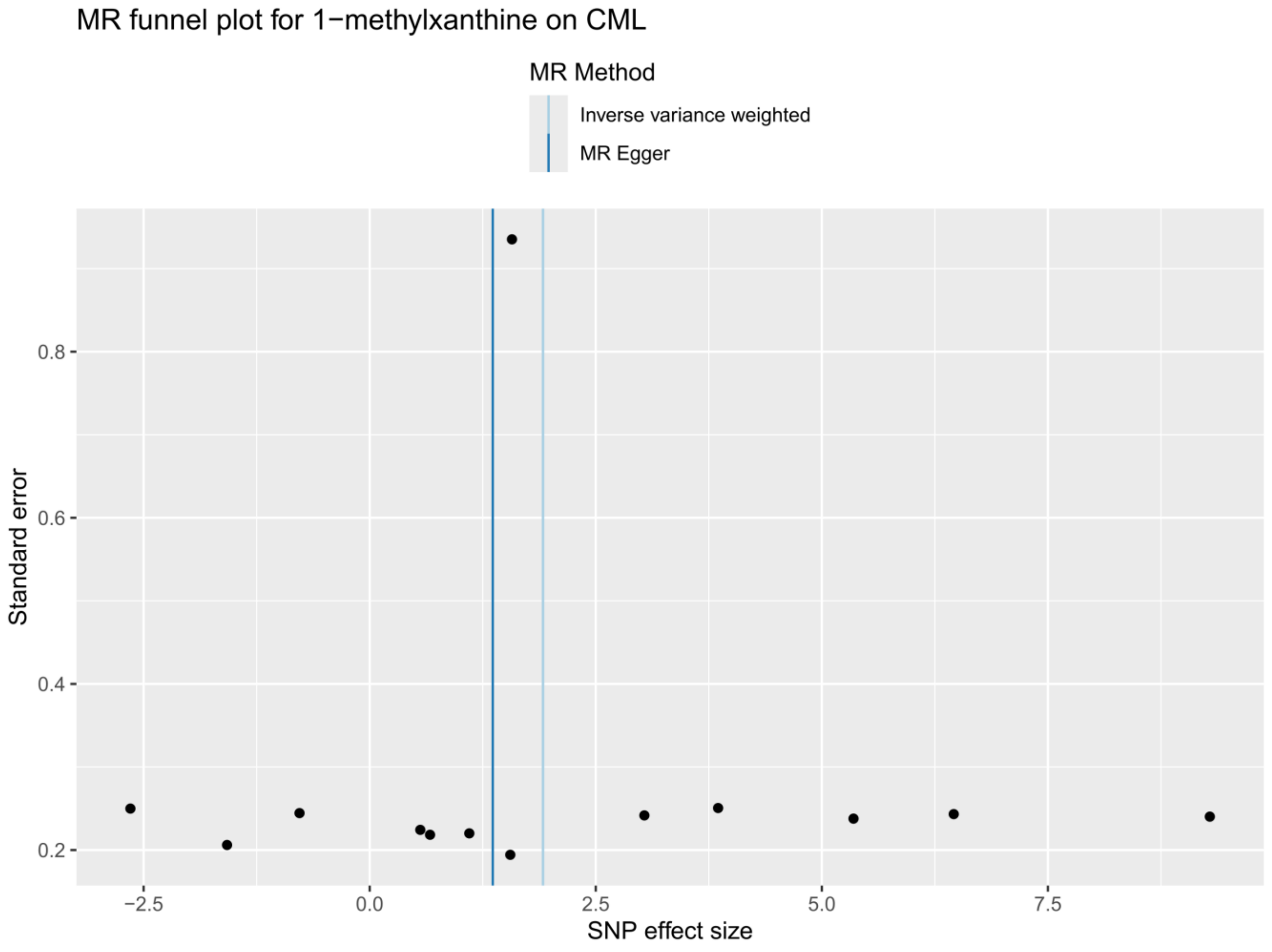
MR funnel plot for 1−methylxanthine on CML
MR Method
Inverse variance weighted
MR Egger
Standard error
0.8
0.6
0.4
0.2
−2.5
0.0
2.5
5.0
7.5
SNP effect size

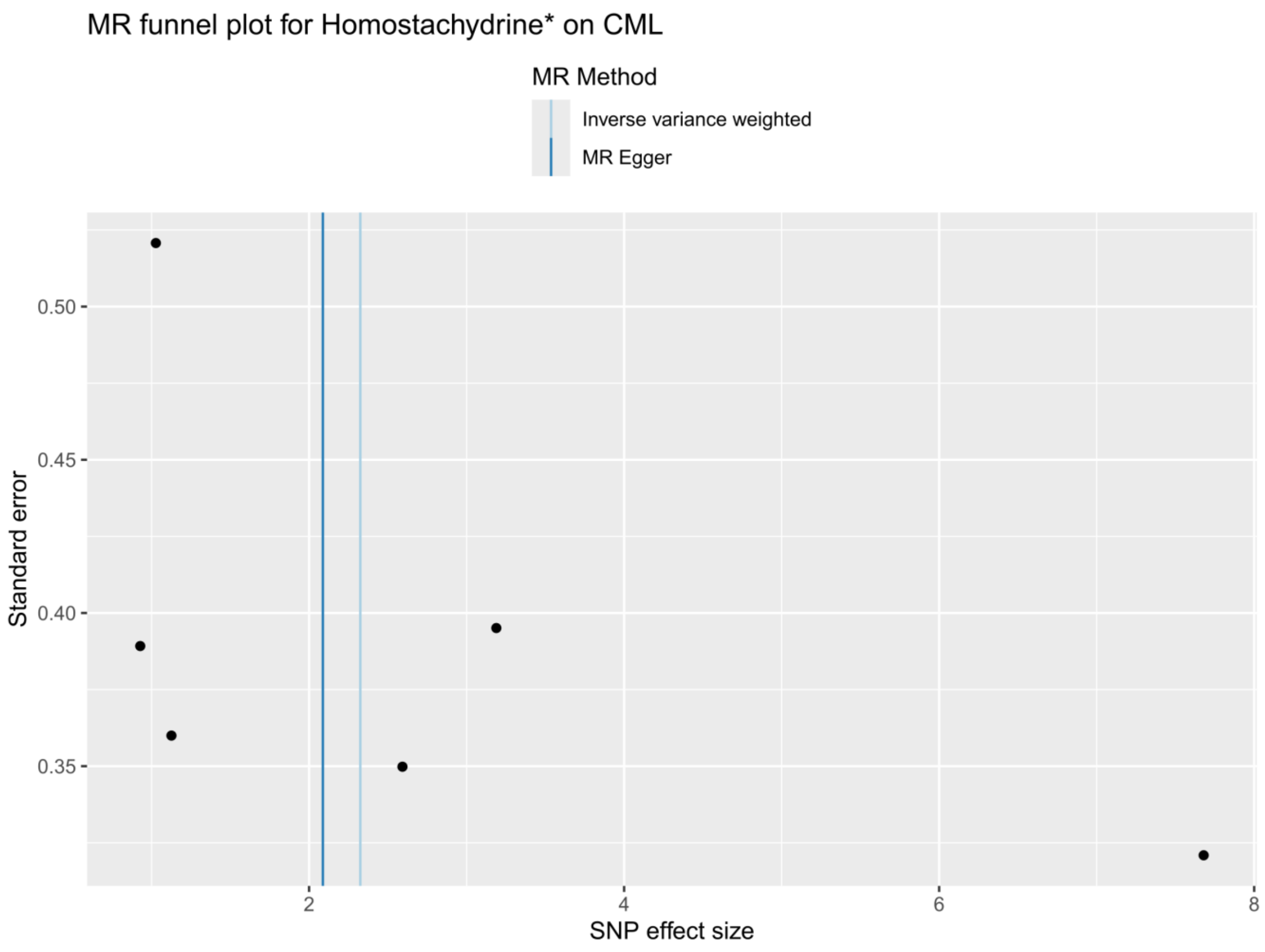
MR funnel plot for Homostachydrine* on CML
MR Method
Inverse variance weighted
MR Egger
Standard error
0.50
0.45
0.40
0.35
2
4
6
8
SNP effect size

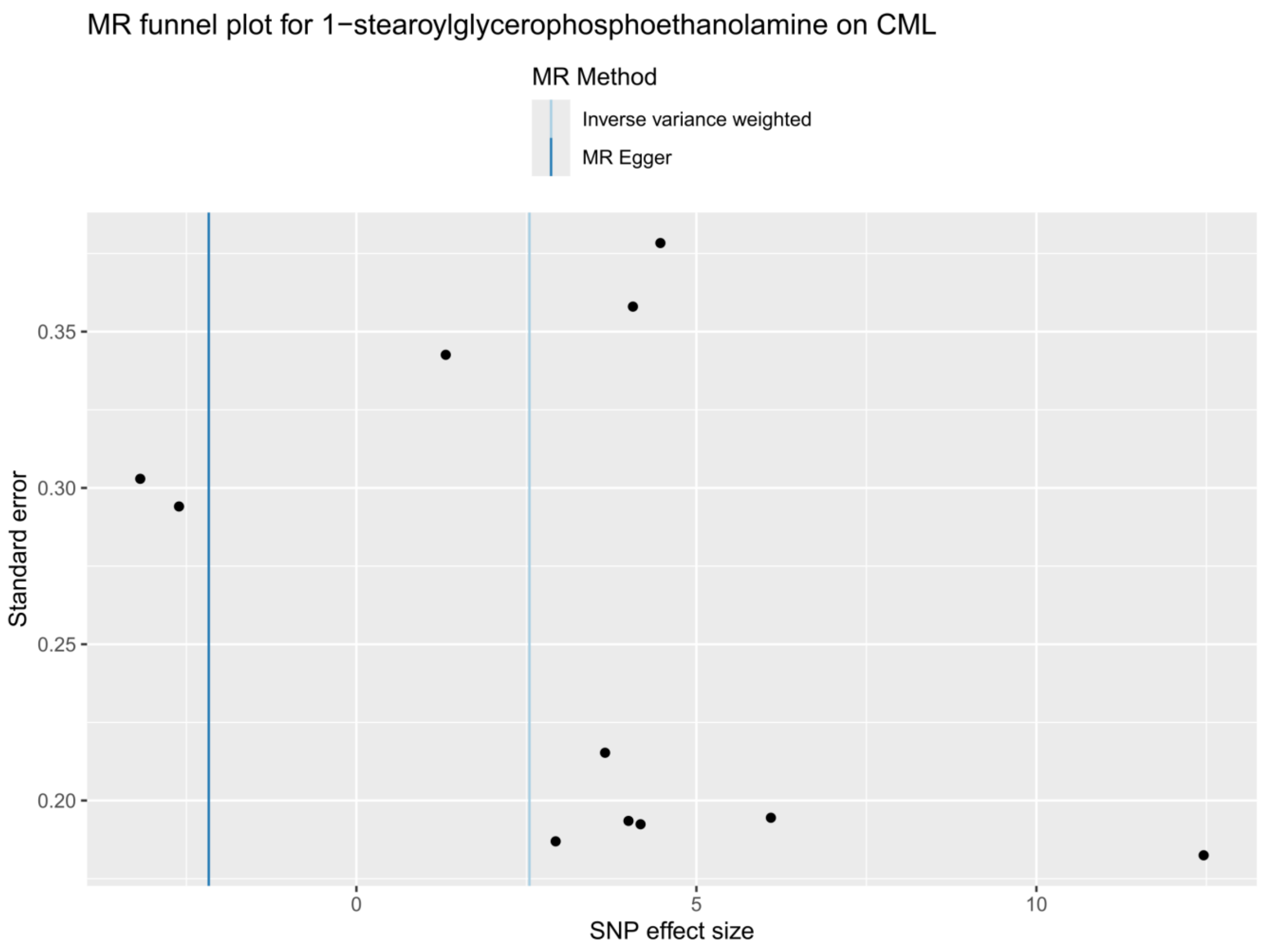
MR funnel plot for 1−stearoylglycerophosphoethanolamine on CML
MR Method
Inverse variance weighted
MR Egger
Standard error
0.35
0.30
0.25
0.20
0
5
10
SNP effect size

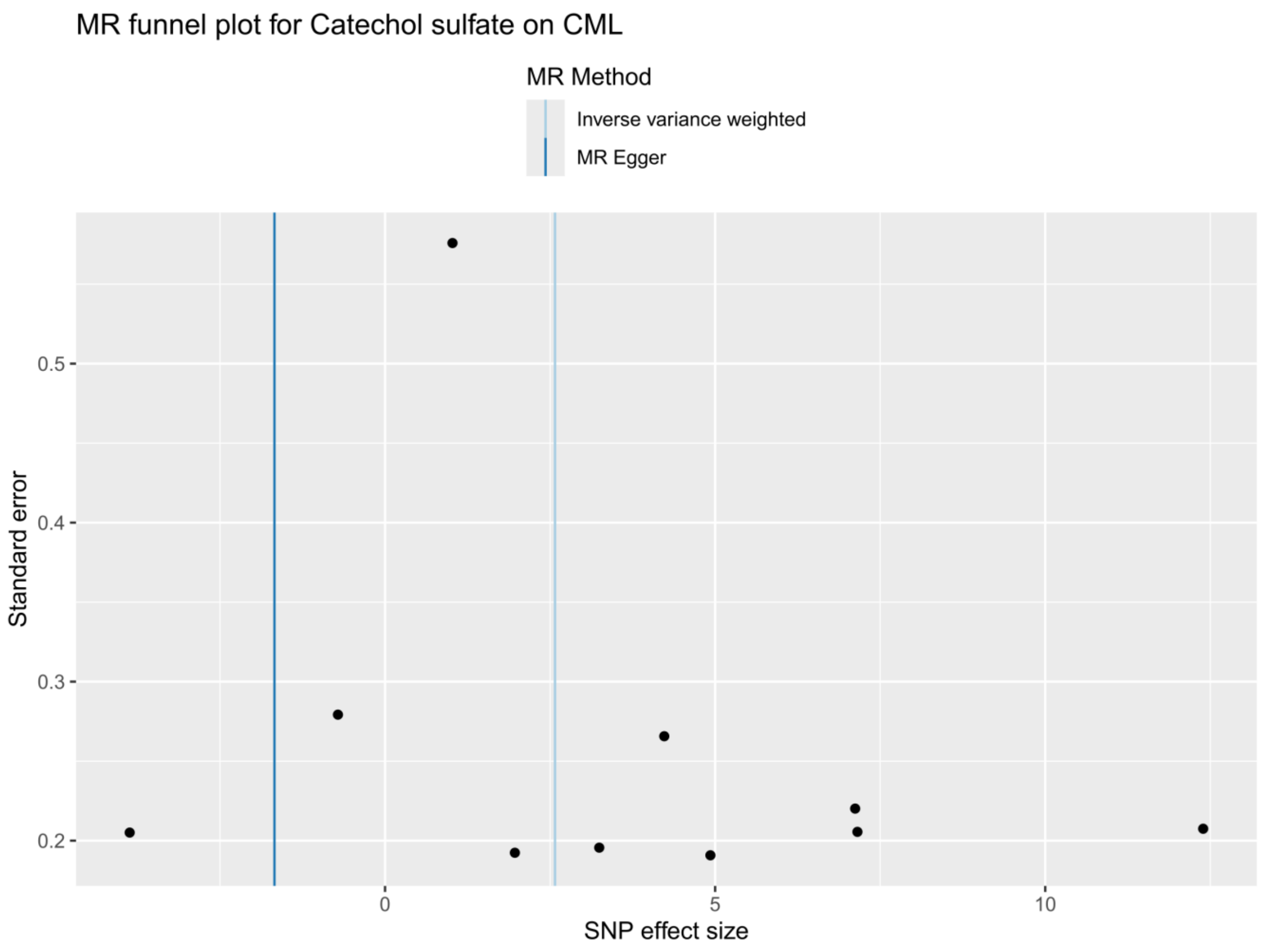
MR funnel plot for Catechol sulfate on CML
MR Method
Inverse variance weighted
MR Egger
Standard error
0.5
0.4
0.3
0.2
0
5
10
SNP effect size

MR funnel plot for Serotonin (5HT) on CML

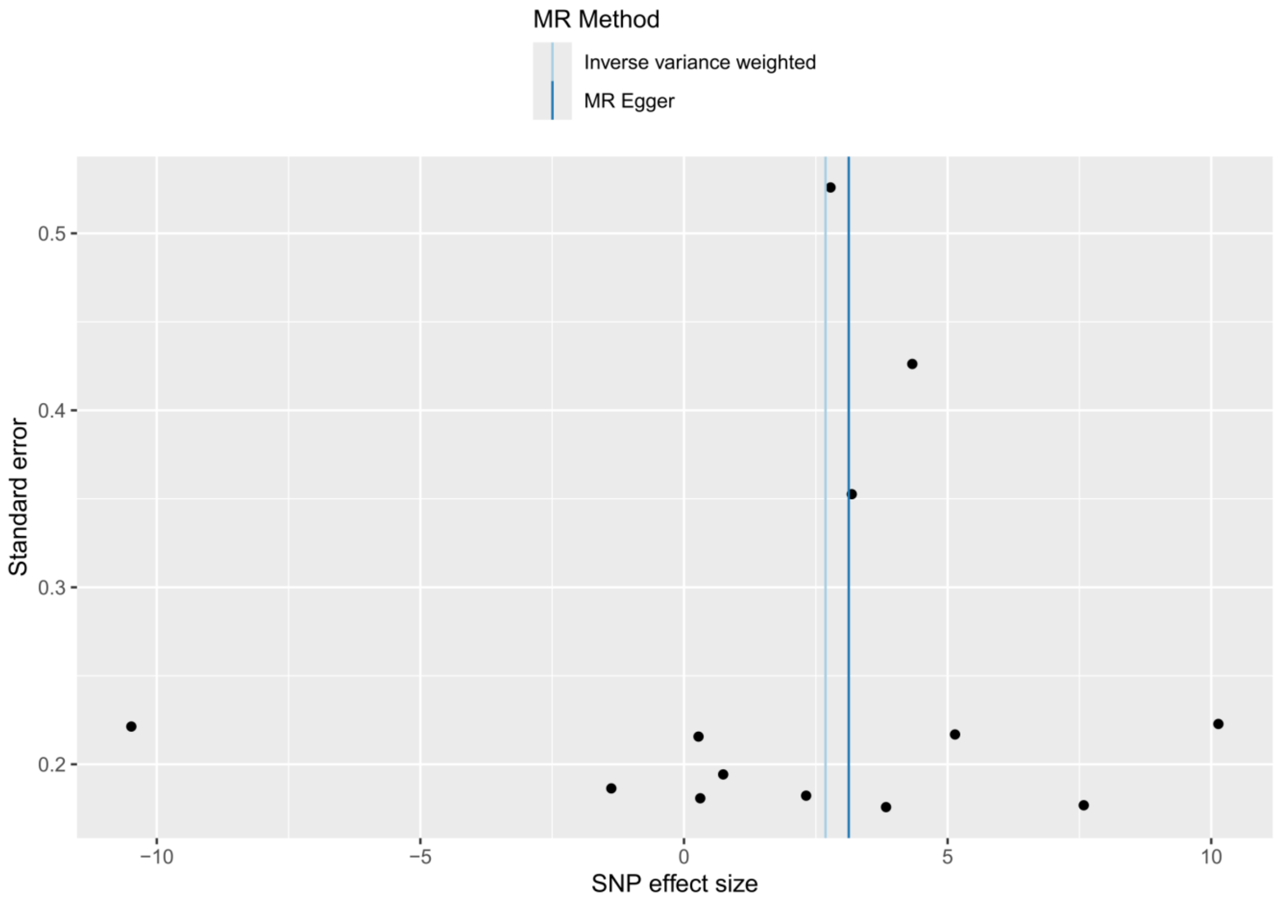

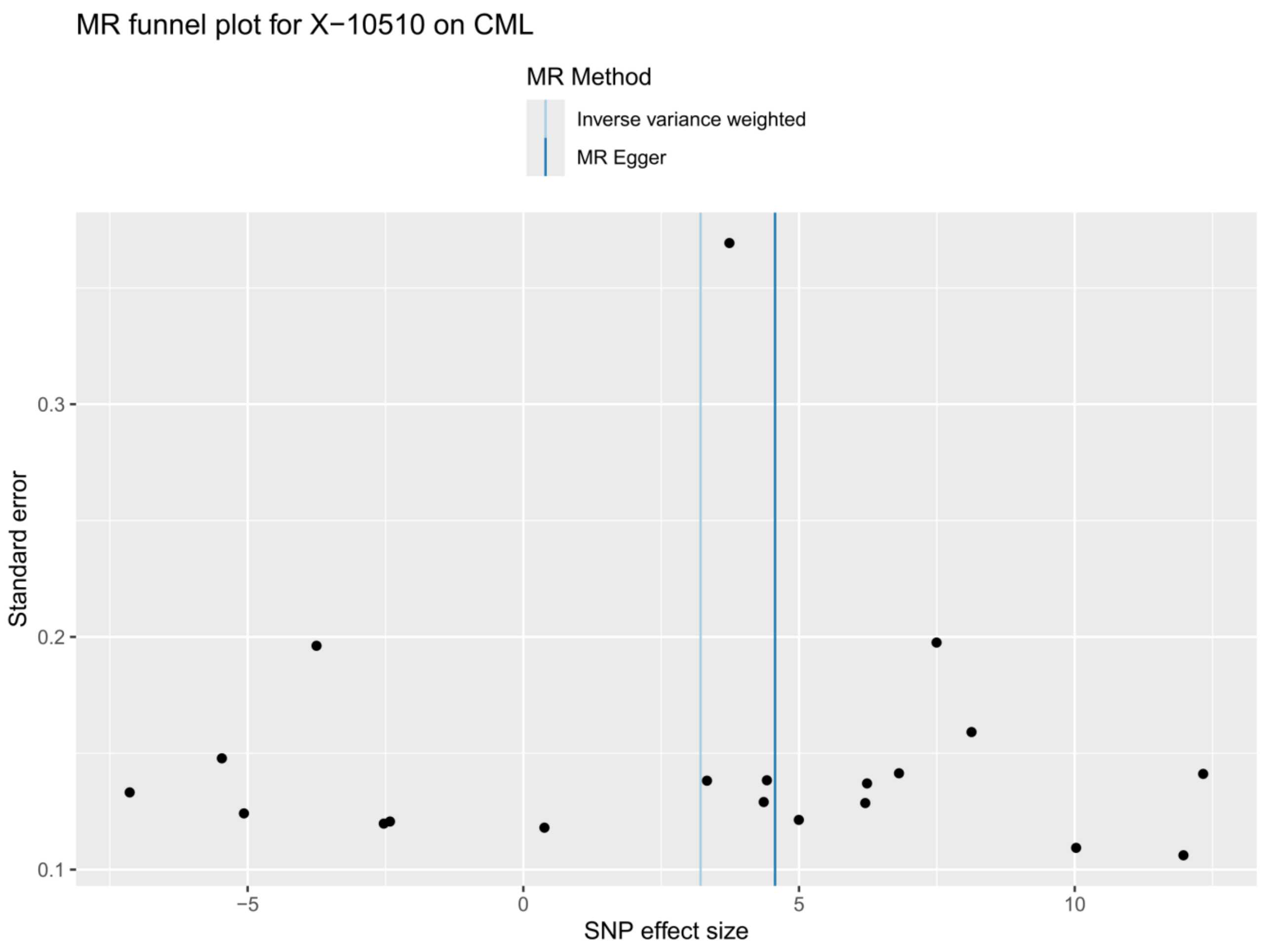

MR funnel plot for X−10510 on CML
MR Method
Inverse variance weighted
MR Egger
Standard error
0.3
0.2
0.1
−5
0
5
10
SNP effect size

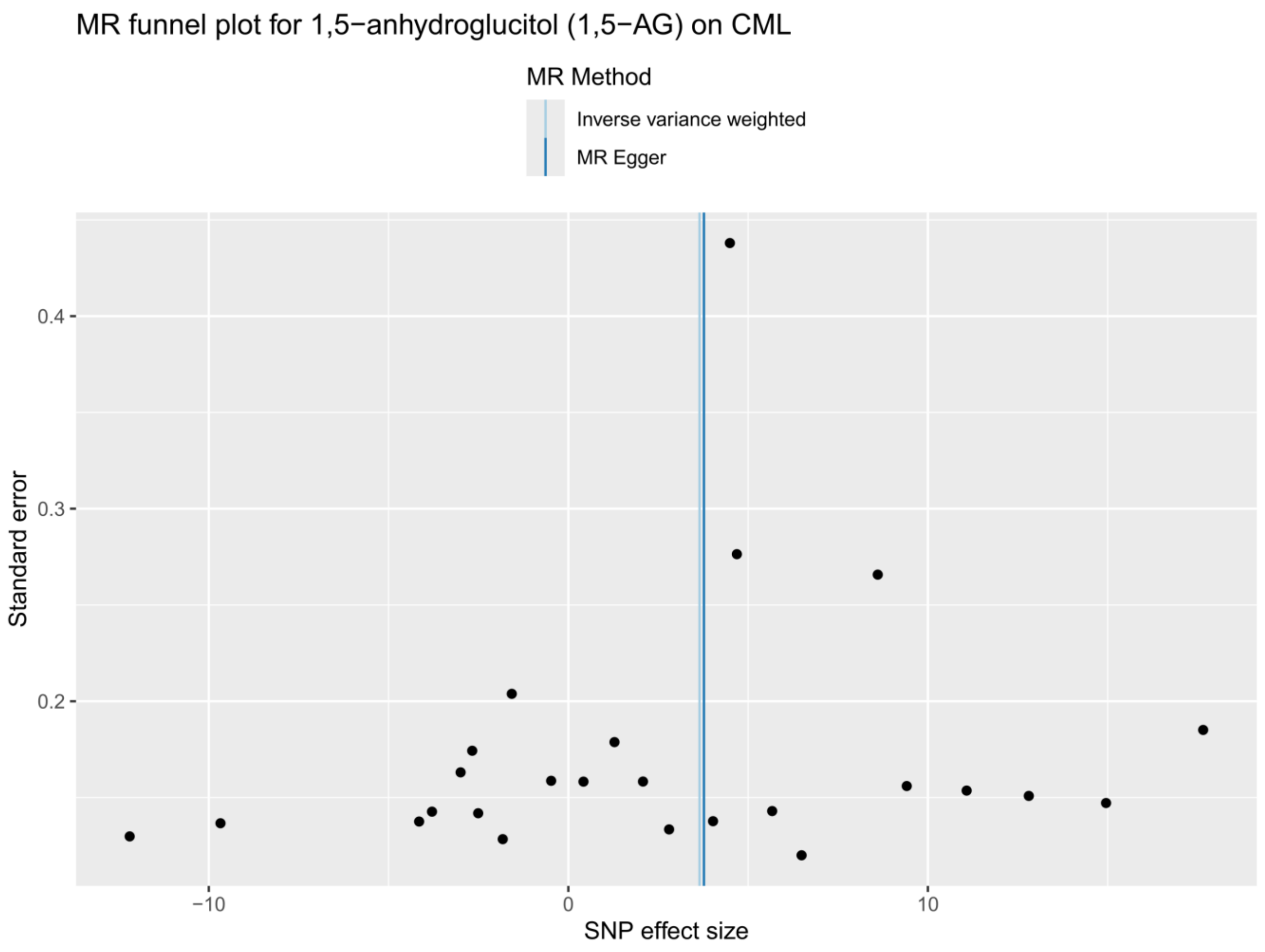
MR funnel plot for 1,5−anhydroglucitol (1,5−AG) on CML
MR Method
Inverse variance weighted
MR Egger
Standard error
0.4
0.3
0.2
−10
0
10
SNP effect size

MR funnel plot for 4−methyl−2−oxopentanoate on CML

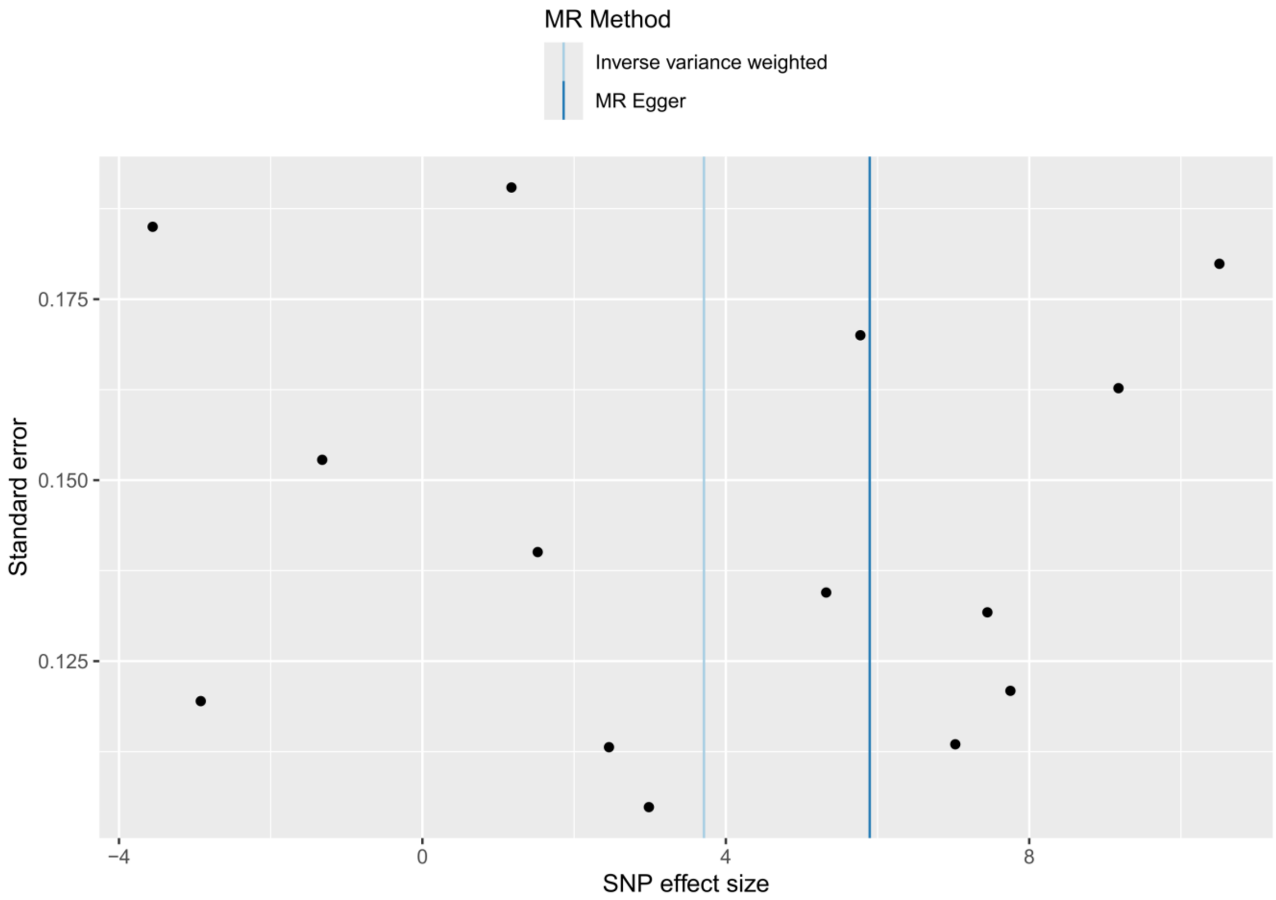

Supplementary Figure S3. Leave-one-out plots for the 20 potential metabolites on CML.

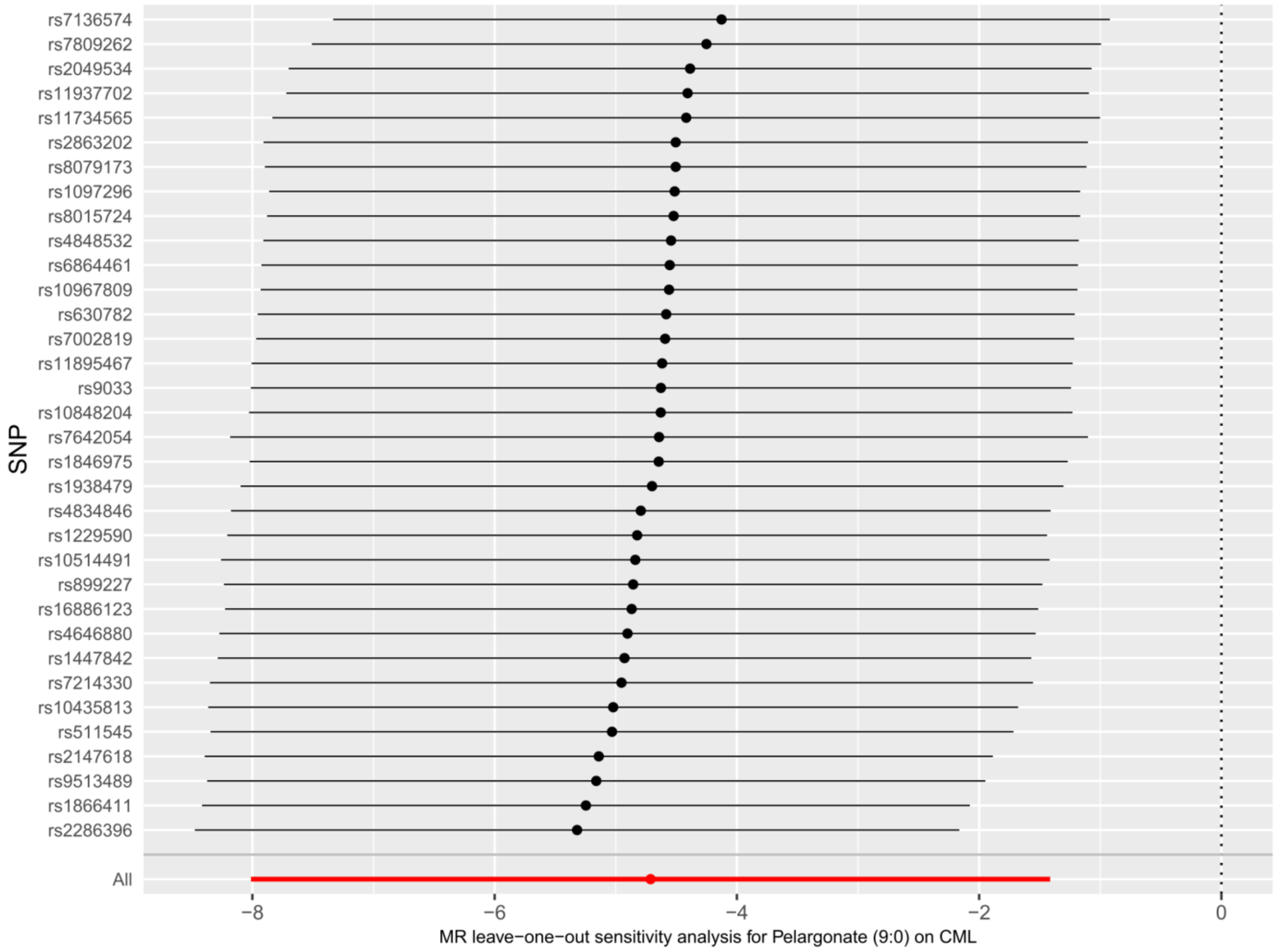

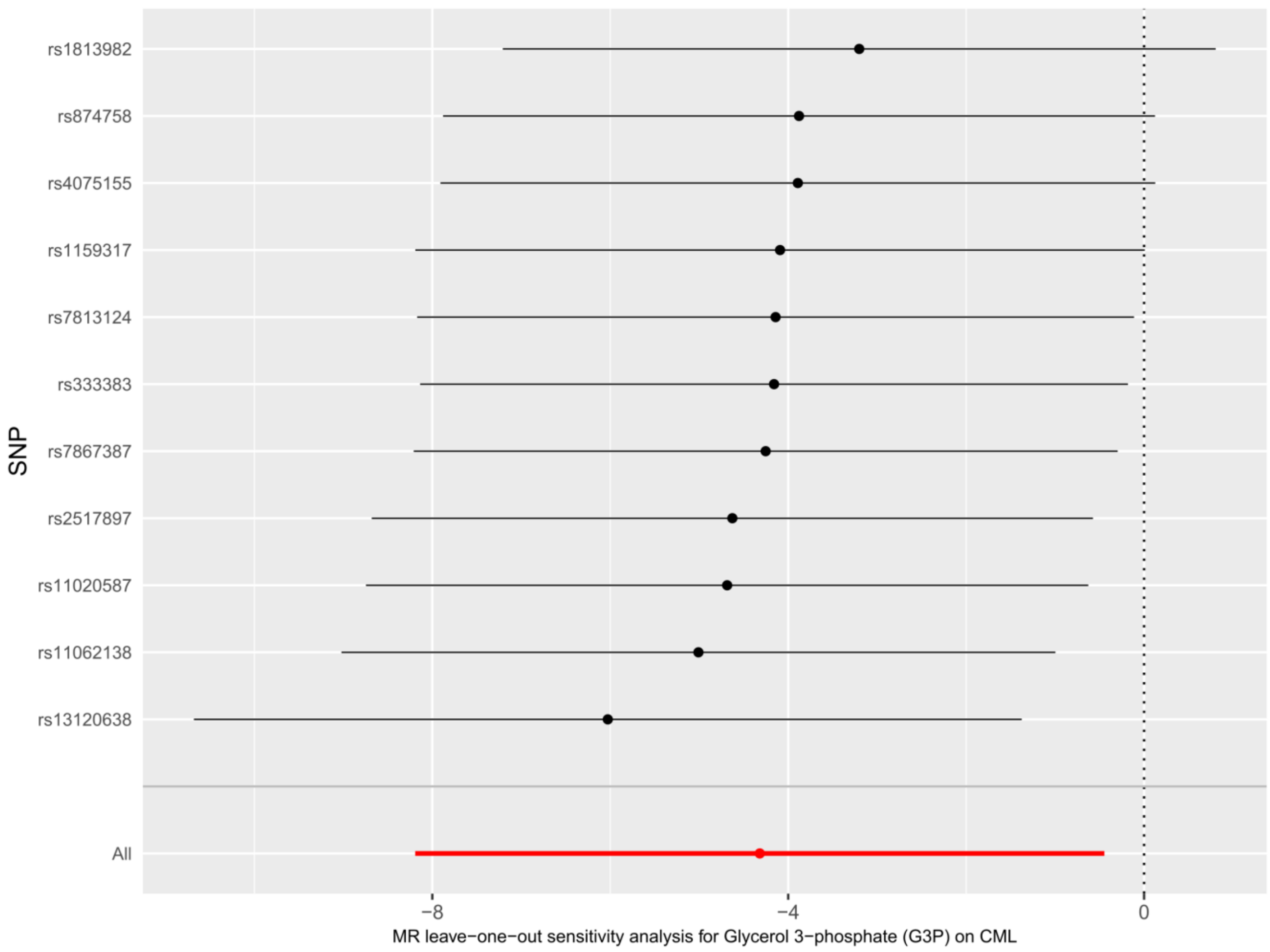

rs1813982
rs874758
rs4075155
rs1159317
rs7813124
rs333383
rs7867387
rs2517897
rs11020587
rs11062138
rs13120638
All
SNP
−8
−4
0
MR leave−one−out sensitivity analysis for Glycerol 3−phosphate (G3P) on CML

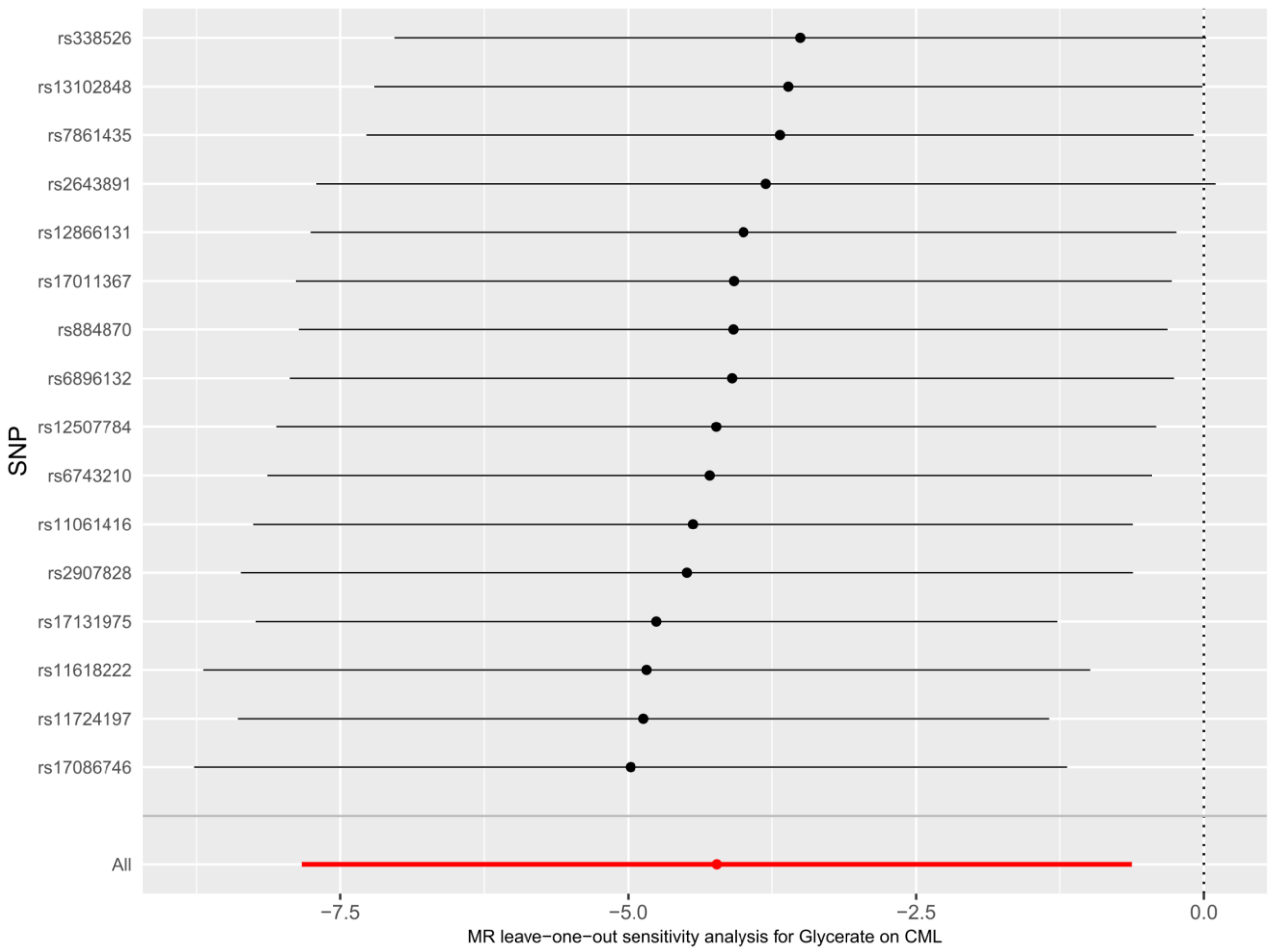
rs338526
rs13102848
rs7861435
rs2643891
rs12866131
rs17011367
rs884870
rs6896132
rs12507784
rs6743210
rs11061416
rs2907828
rs17131975
rs11618222
rs11724197
rs17086746
All
SNP
−7.5
−5.0
−2.5
0.0
MR leave−one−out sensitivity analysis for Glycerate on CML

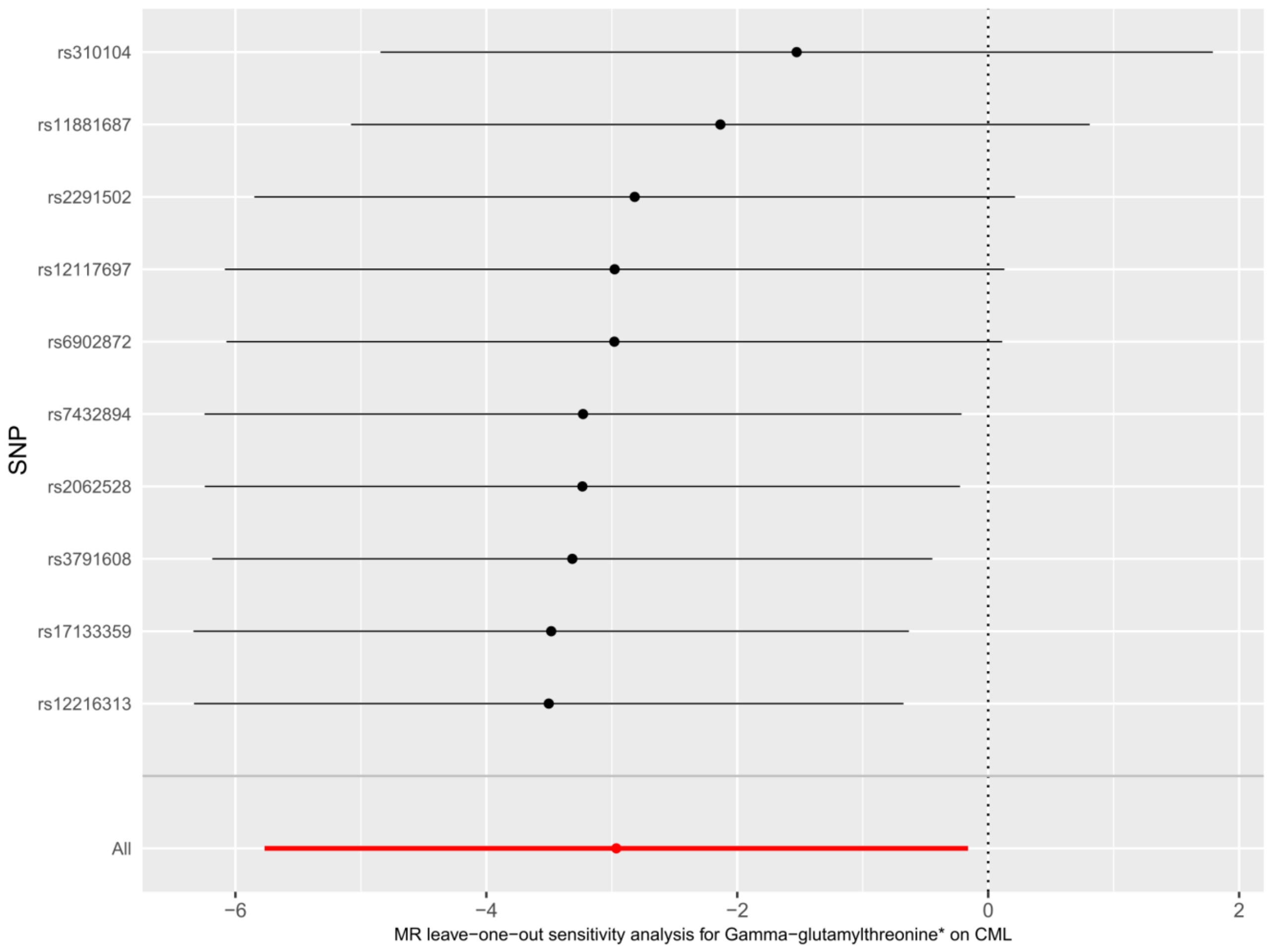

rs310104
rs11881687
rs2291502
rs12117697
rs6902872
rs7432894
rs2062528
rs3791608
rs17133359
rs12216313
All
SNP
−6
−4
−2
0
2
MR leave−one−out sensitivity analysis for Gamma−glutamylthreonine* on CML

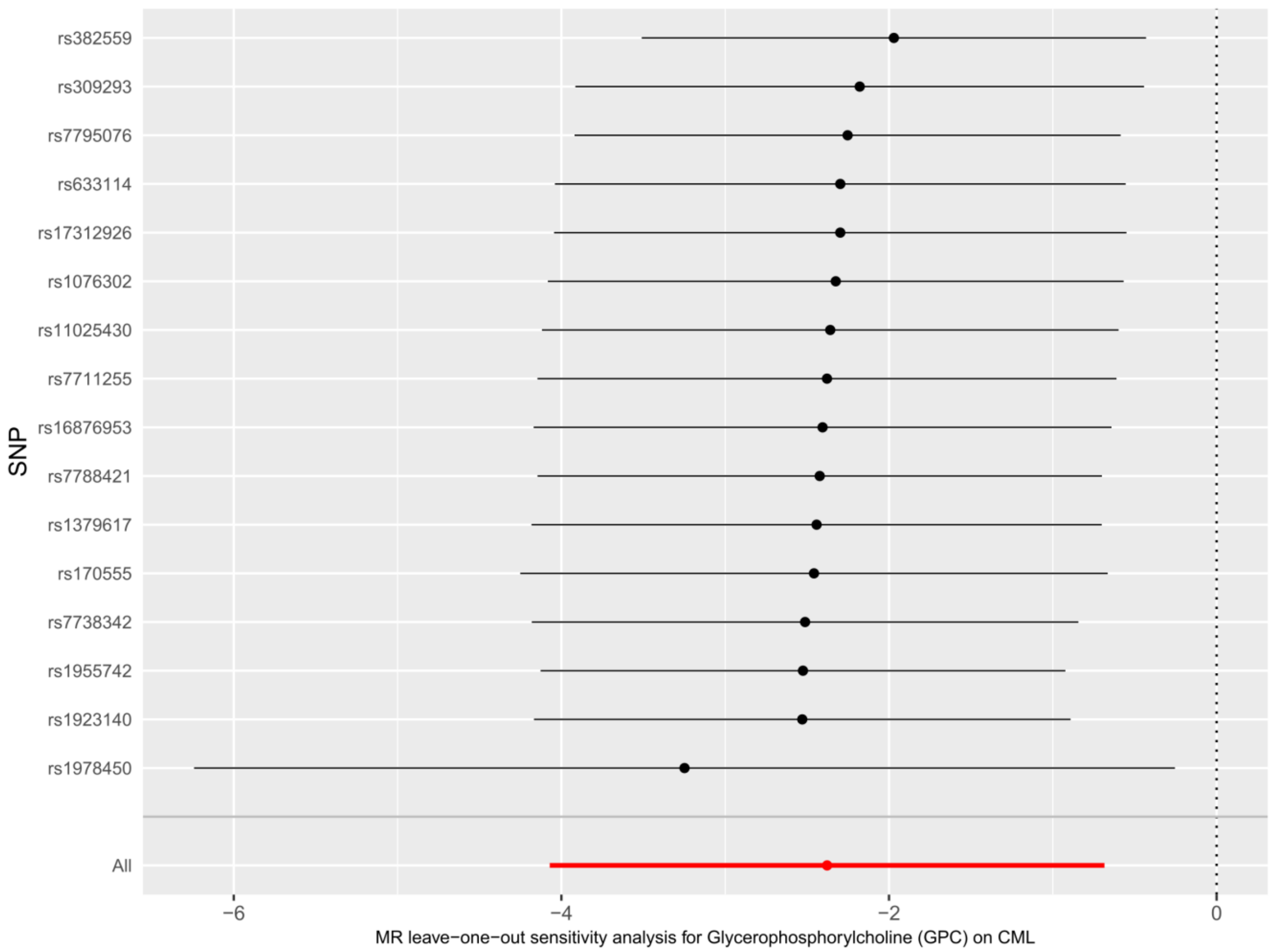
rs382559
rs309293
rs7795076
rs633114
rs17312926
rs1076302
rs11025430
rs7711255
rs16876953
rs7788421
rs1379617
rs170555
rs7738342
rs1955742
rs1923140
rs1978450
All
SNP
−6
−4
−2
0
MR leave−one−out sensitivity analysis for Glycerophosphorylcholine (GPC) on CML

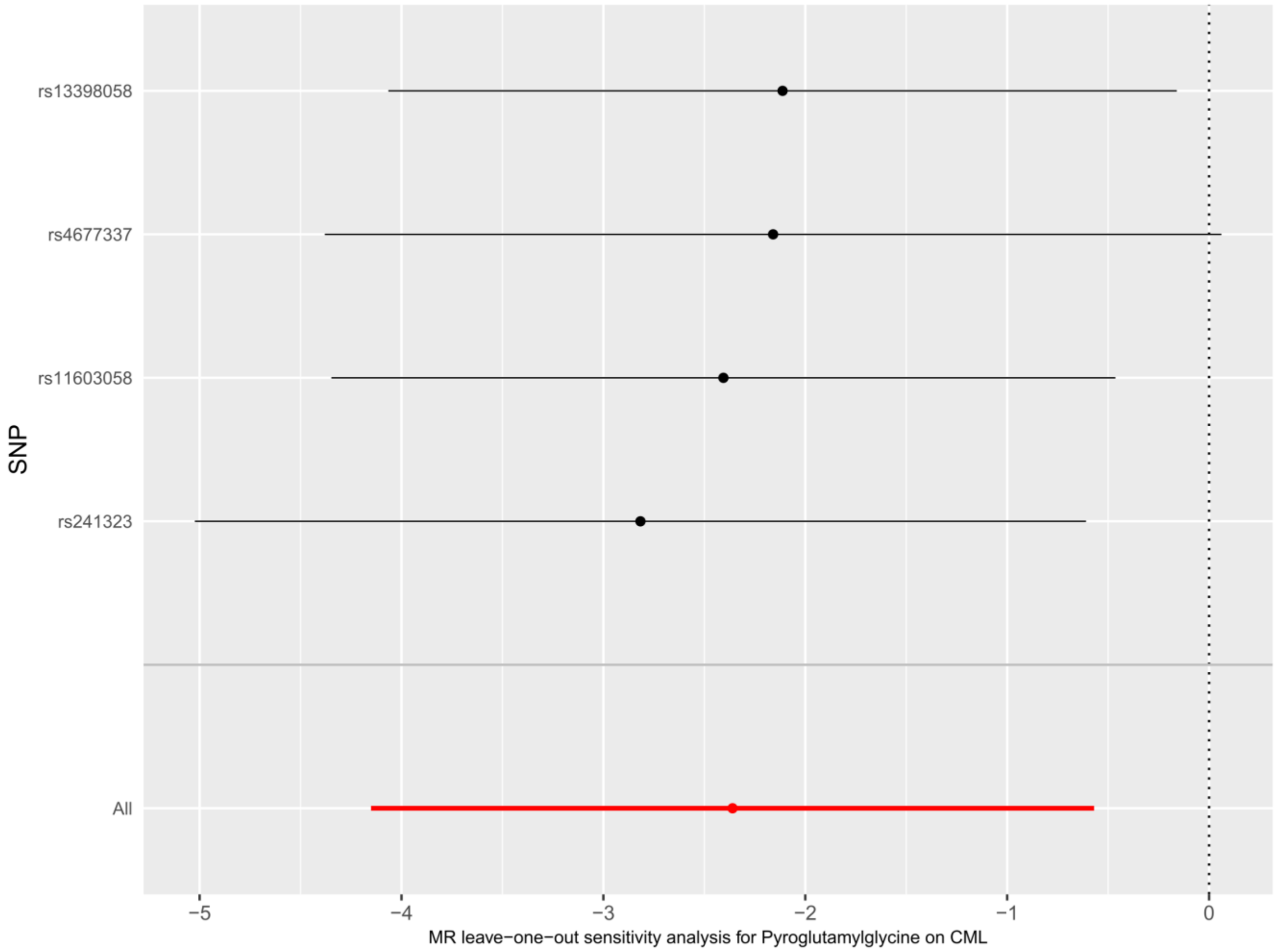

SNP
rs13398058
rs4677337
rs11603058
rs241323
All
−5
−4
−3
−2
−1
0
MR leave−one−out sensitivity analysis for Pyroglutamylglycine on CML

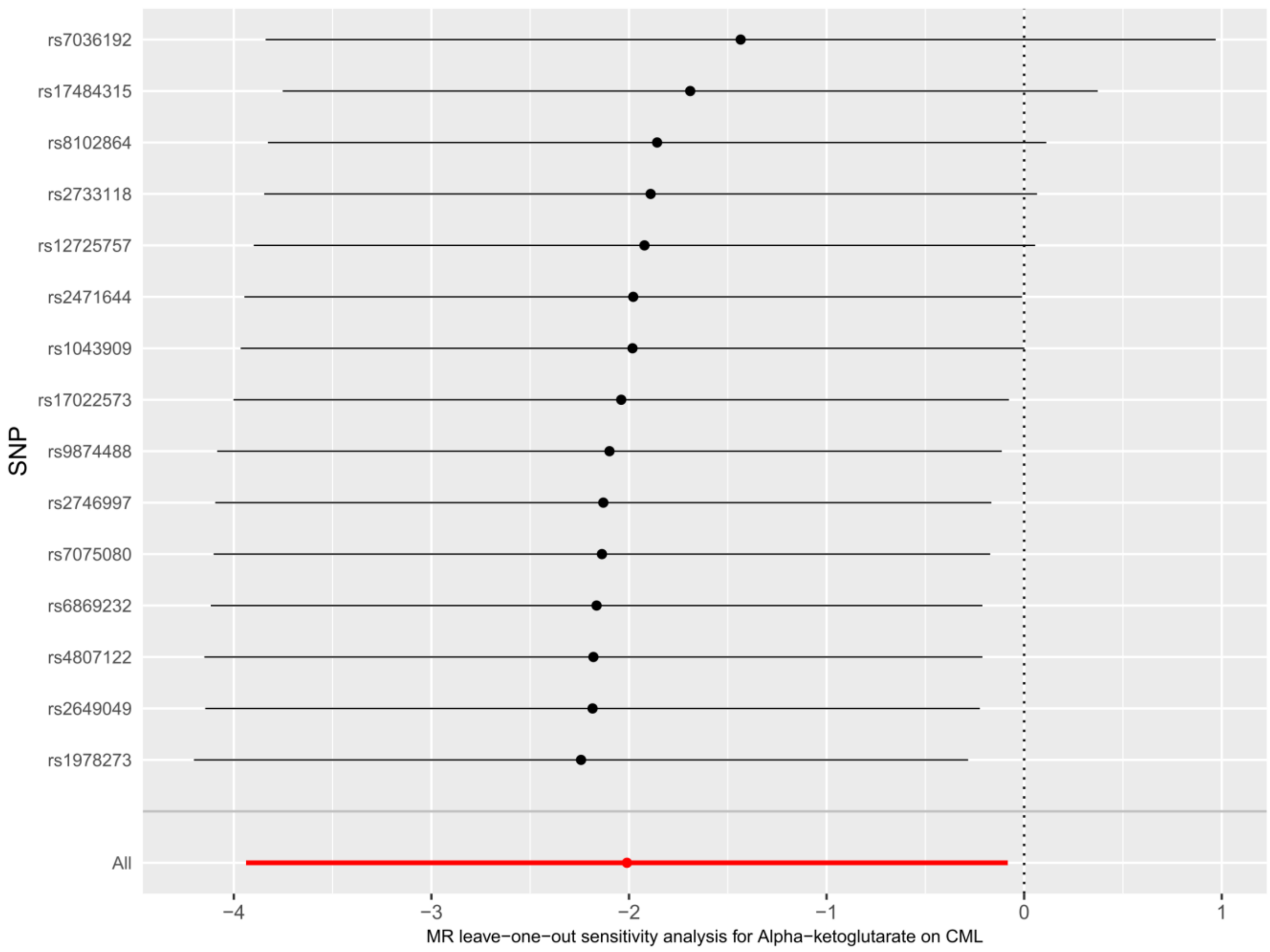

rs7036192
rs17484315
rs8102864
rs2733118
rs12725757
rs2471644
rs1043909
rs17022573
rs9874488
rs2746997
rs7075080
rs6869232
rs4807122
rs2649049
rs1978273
All
SNP
−4
−3
−2
−1
0
1
MR leave−one−out sensitivity analysis for Alpha−ketoglutarate on CML

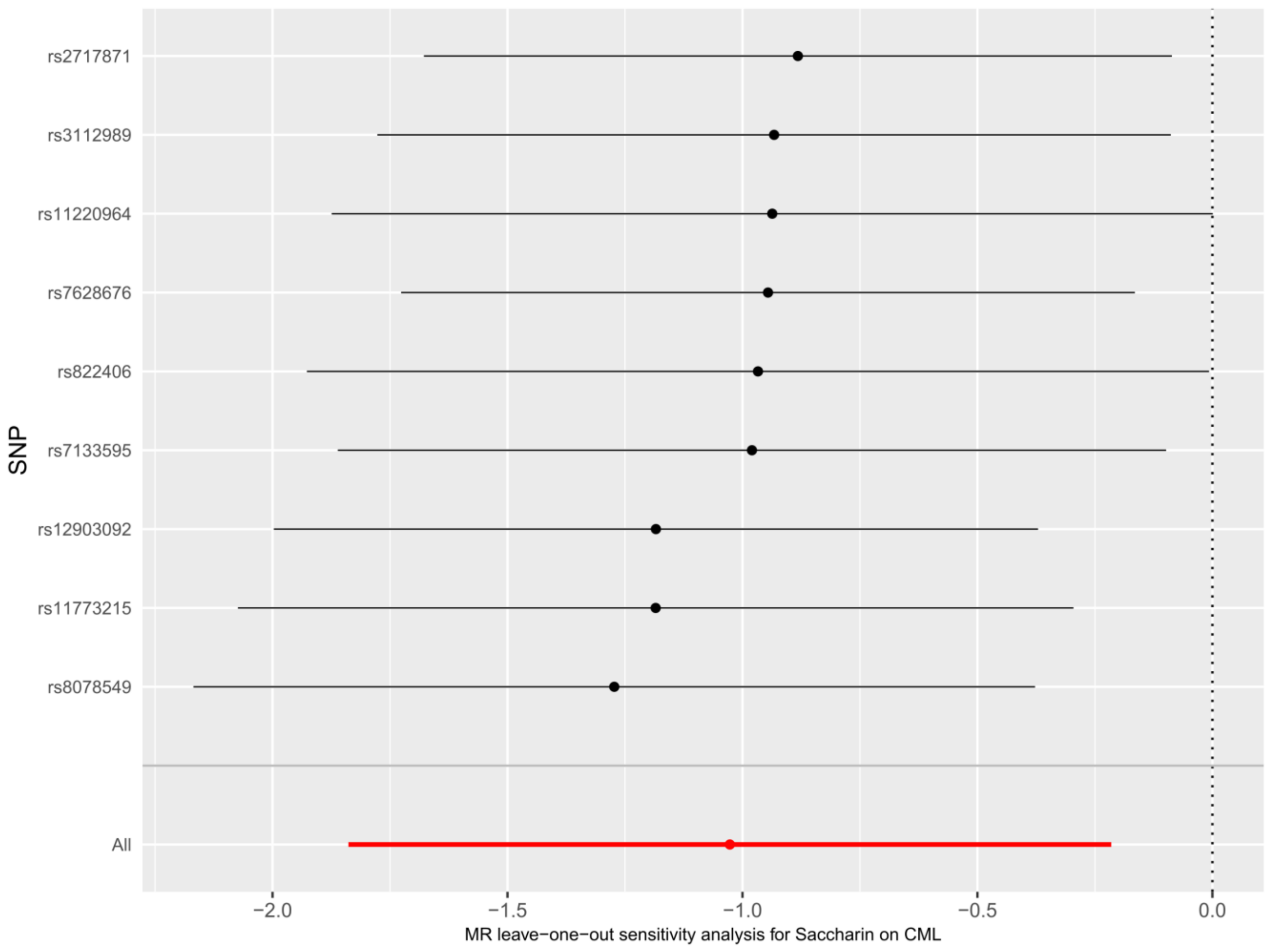

SNP
rs2717871
rs3112989
rs11220964
rs7628676
rs822406
rs7133595
rs12903092
rs11773215
rs8078549
All
−2.0
−1.5
−1.0
−0.5
0.0
MR leave−one−out sensitivity analysis for Saccharin on CML

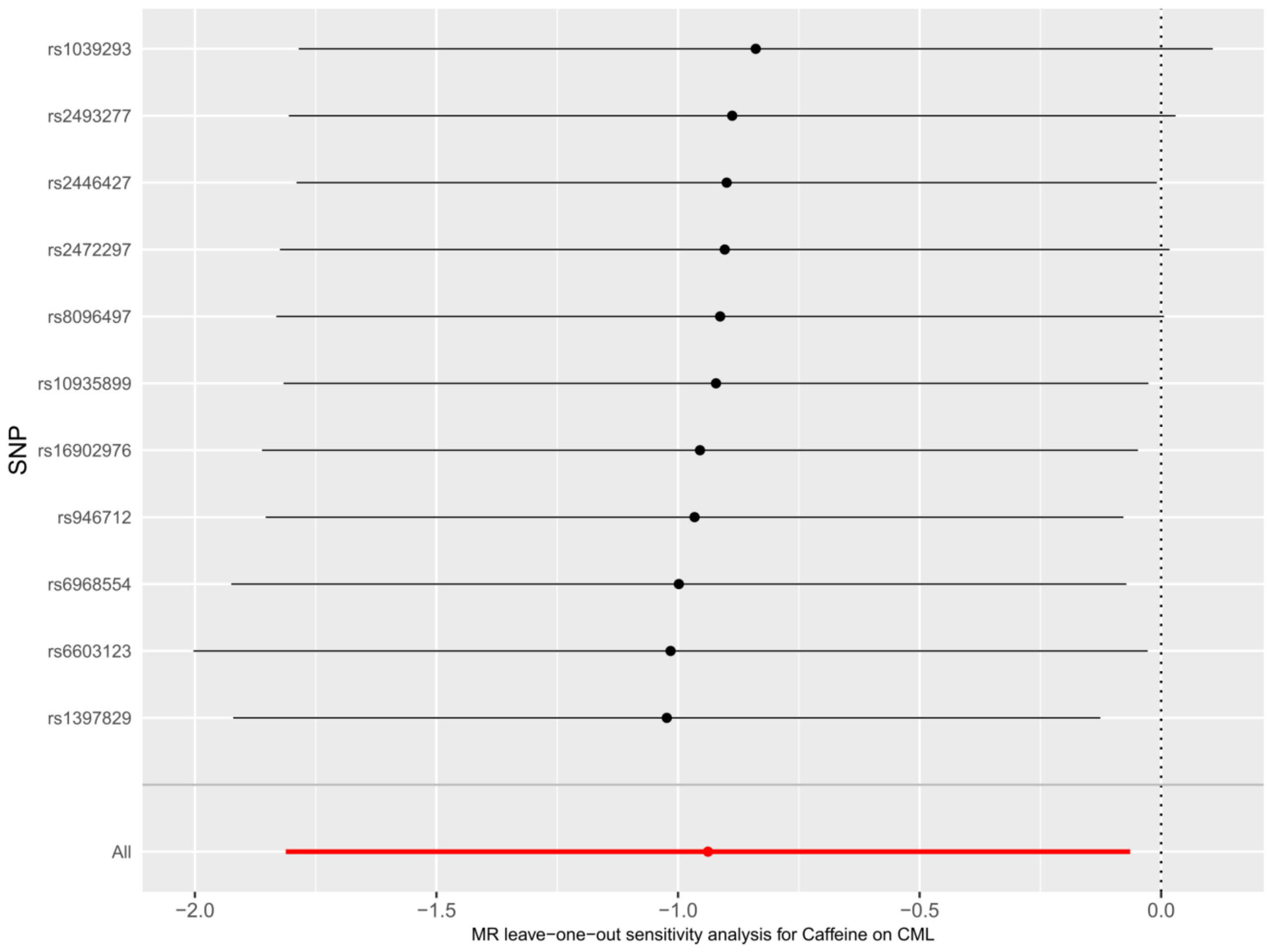

SNP
rs1039293
rs2493277
rs2446427
rs2472297
rs8096497
rs10935899
rs16902976
rs946712
rs6968554
rs6603123
rs1397829
All
−2.0
−1.5
−1.0
−0.5
0.0
MR leave−one−out sensitivity analysis for Caffeine on CML

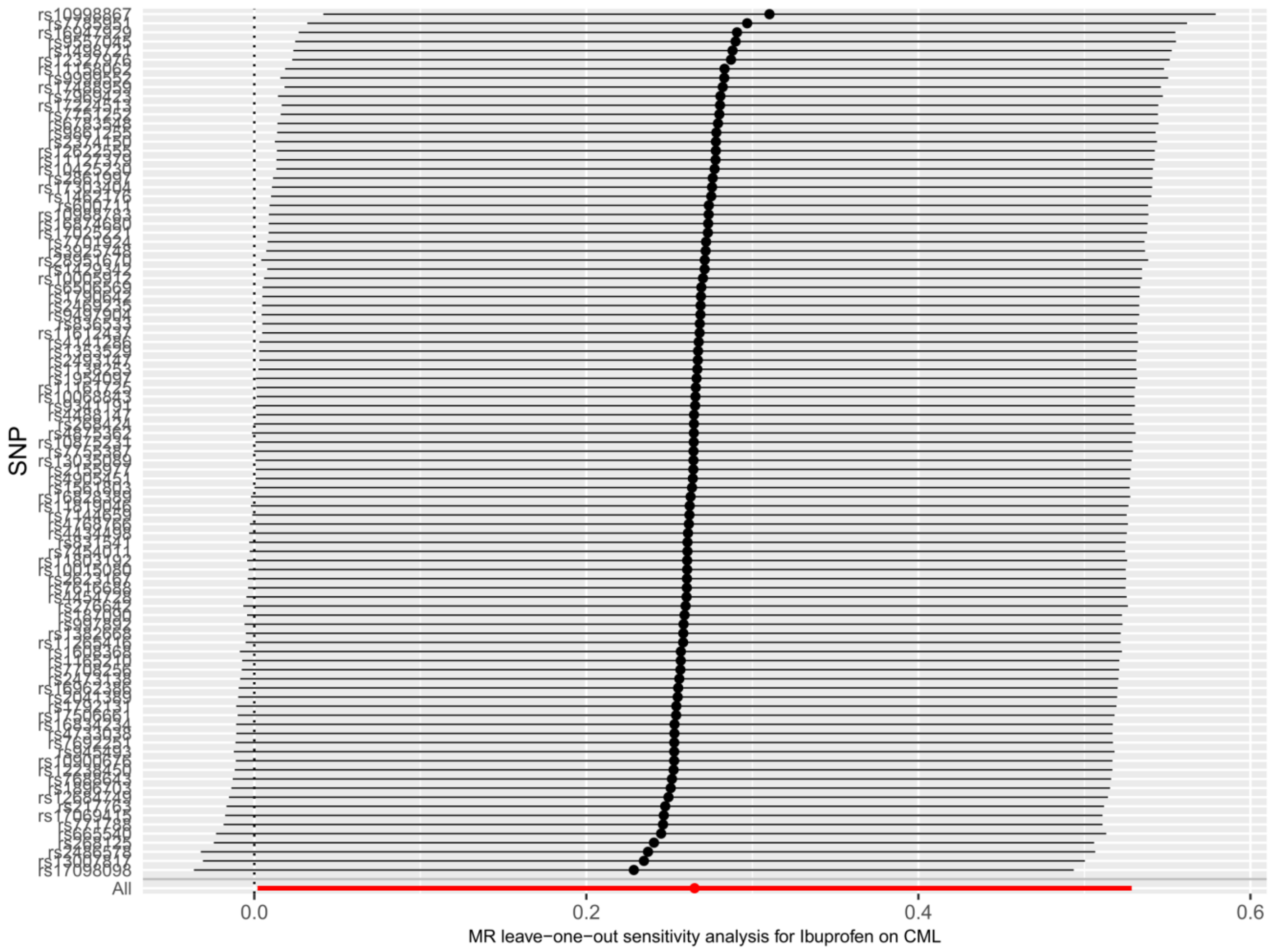

SNP
rs10998867
rs17098098
All
0.0
0.2
0.4
0.6
MR leave−one−out sensitivity analysis for Ibuprofen on CML

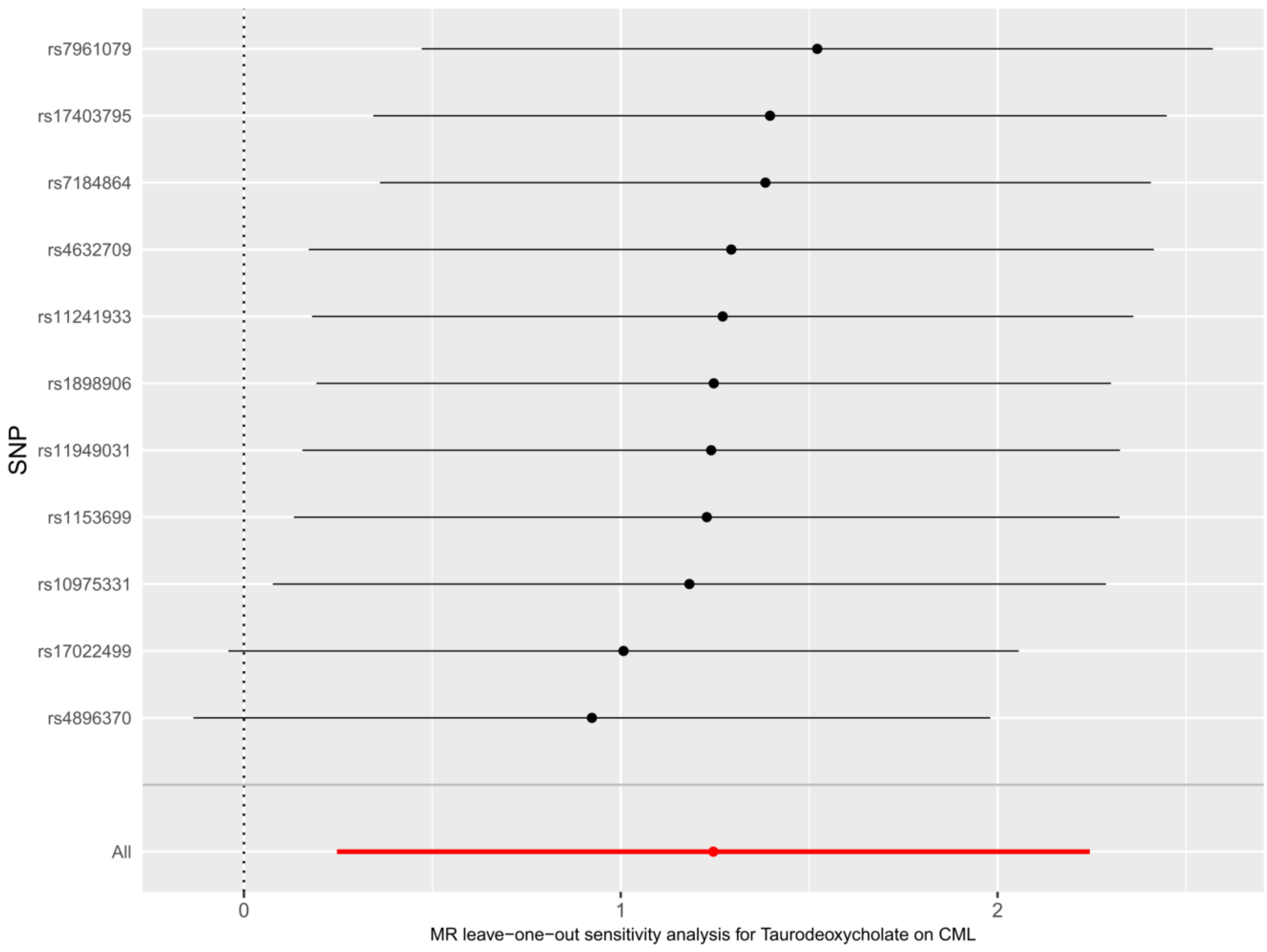
SNP
rs7961079
rs17403795
rs7184864
rs4632709
rs11241933
rs1898906
rs11949031
rs1153699
rs10975331
rs17022499
rs4896370
All
0
1
2
MR leave−one−out sensitivity analysis for Taurodeoxycholate on CML

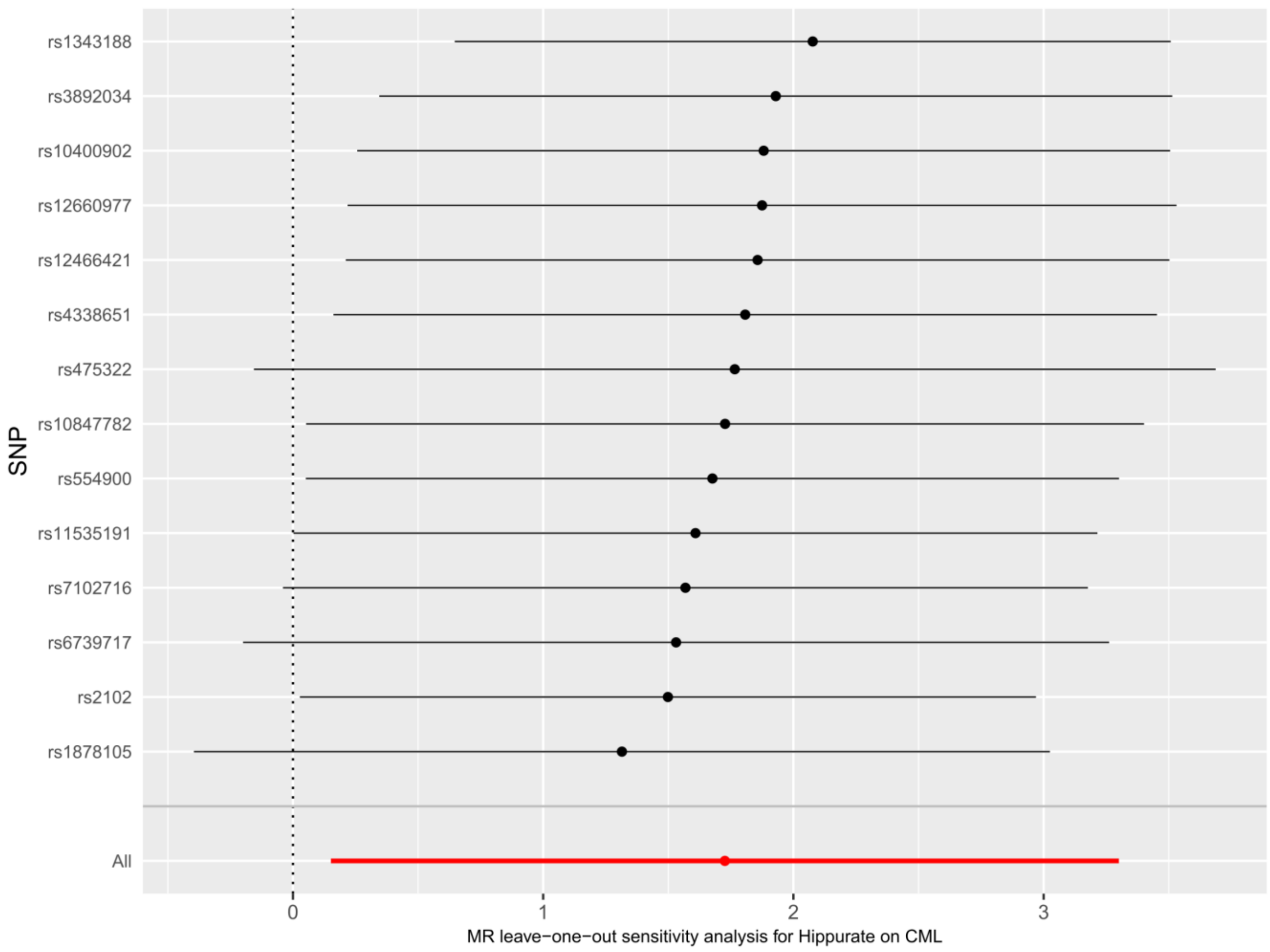
rs1343188
rs3892034
rs10400902
rs12660977
rs12466421
rs4338651
rs475322
rs10847782
rs554900
rs11535191
rs7102716
rs6739717
rs2102
rs1878105
All
SNP
0
1
2
3
MR leave−one−out sensitivity analysis for Hippurate on CML

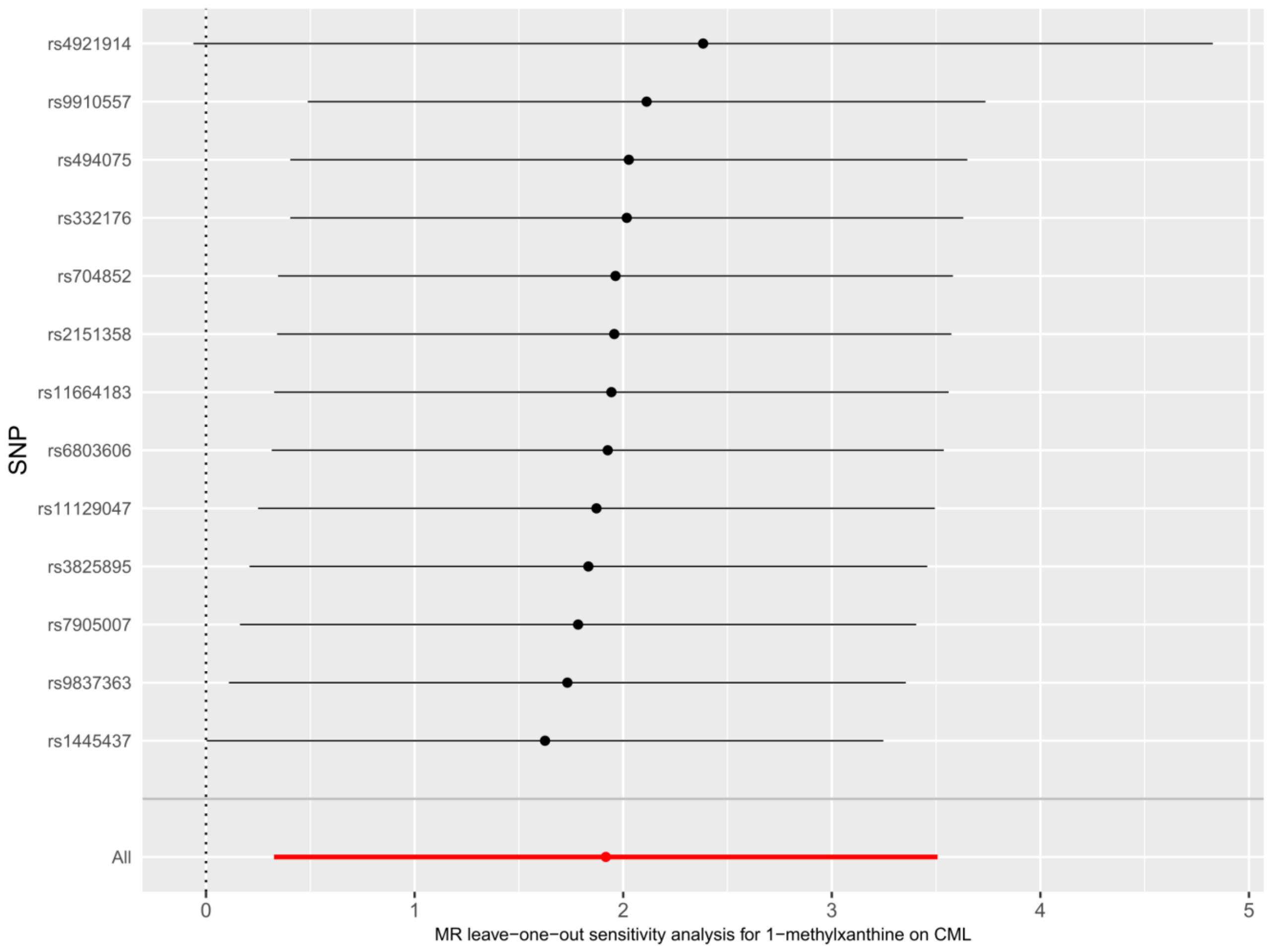

SNP
rs4921914
rs9910557
rs494075
rs332176
rs704852
rs2151358
rs11664183
rs6803606
rs11129047
rs3825895
rs7905007
rs9837363
rs1445437
All
0
1
2
3
4
5
MR leave−one−out sensitivity analysis for 1−methylxanthine on CML

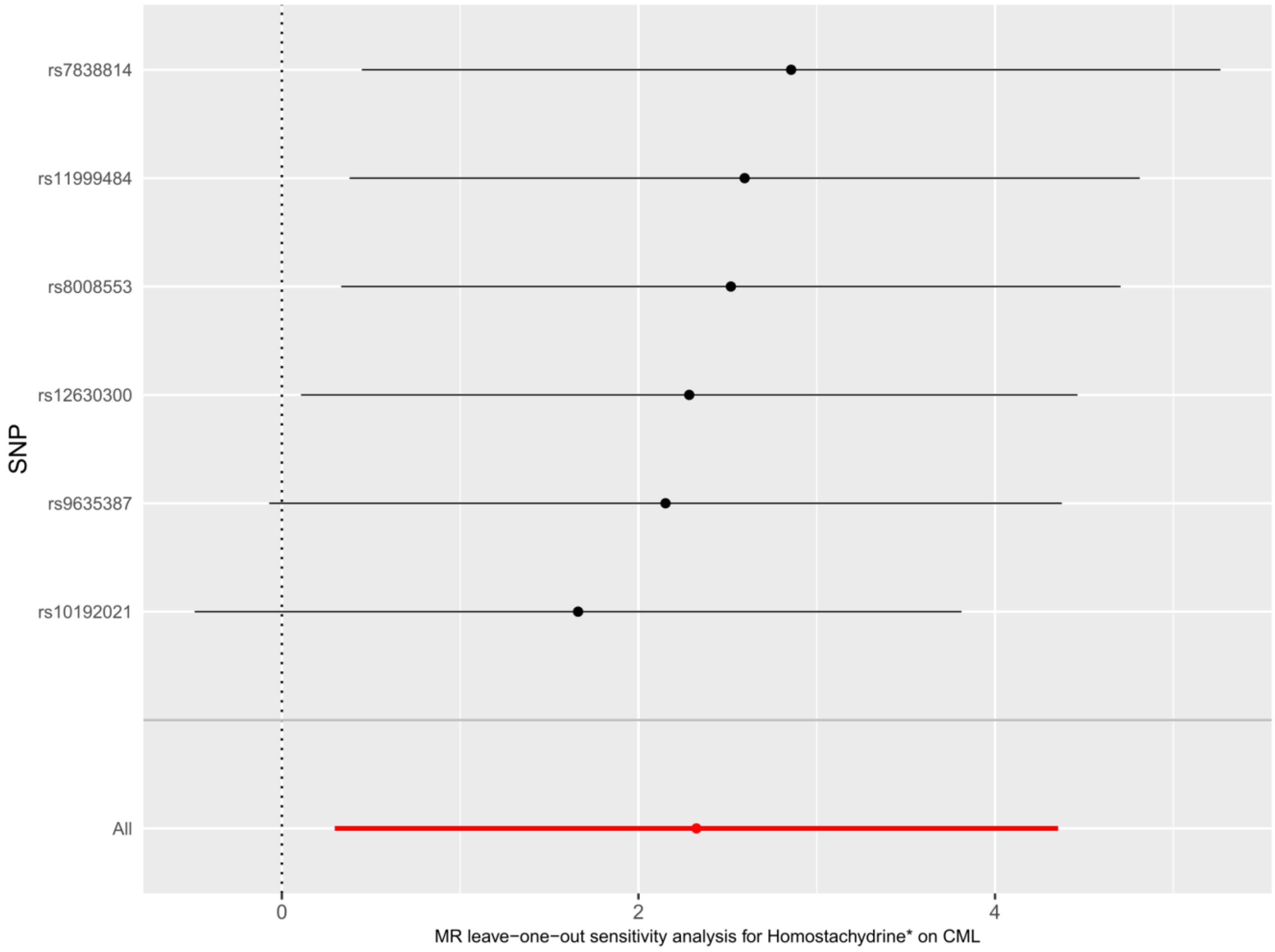

rs7838814
rs11999484
rs8008553
rs12630300
rs9635387
rs10192021
All
SNP
0
2
4
MR leave−one−out sensitivity analysis for Homostachydrine* on CML

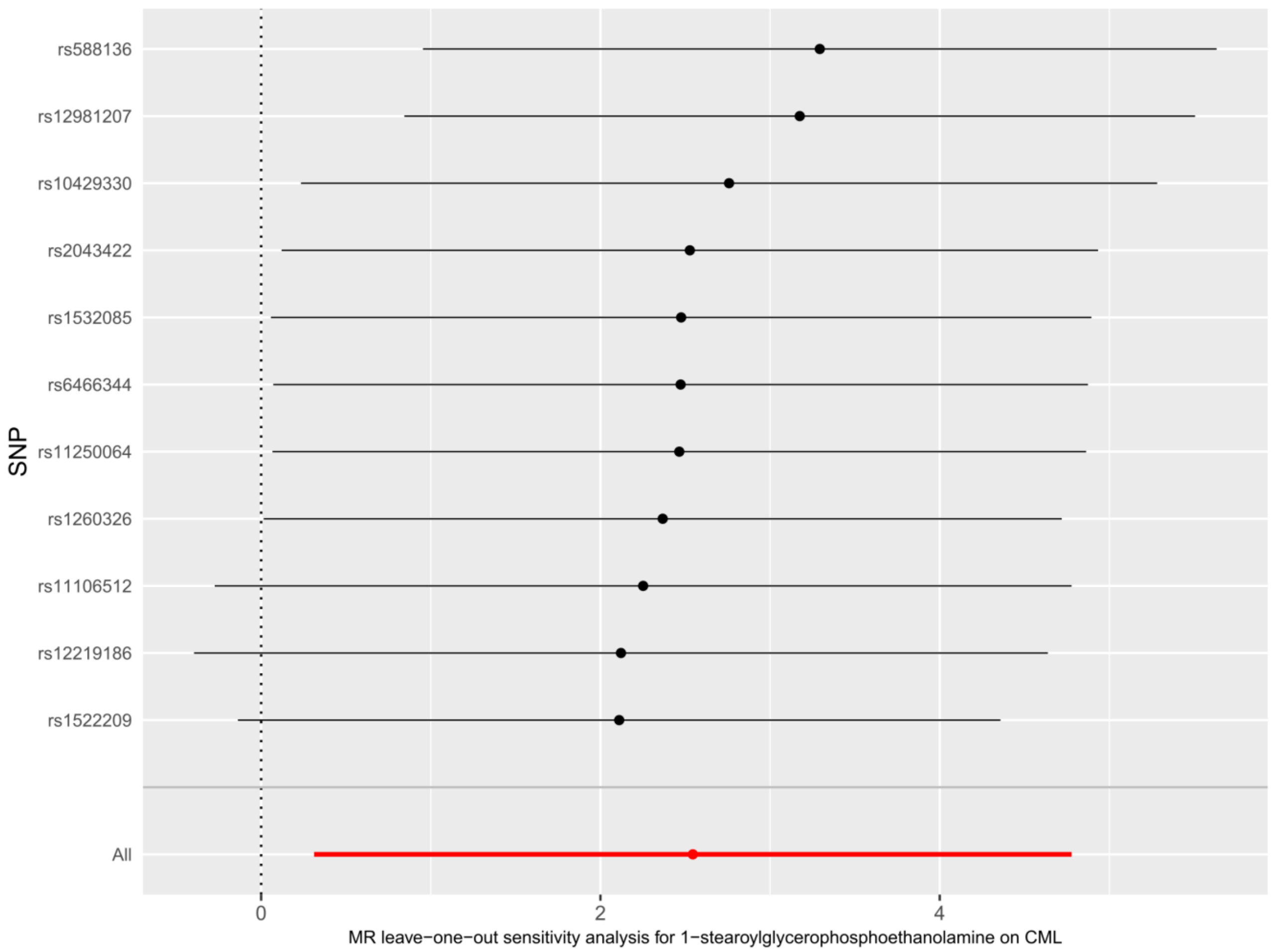
rs588136
rs12981207
rs10429330
rs2043422
rs1532085
rs6466344
rs11250064
rs1260326
rs11106512
rs12219186
rs1522209
All
SNP
0
2
4
MR leave−one−out sensitivity analysis for 1−stearoylglycerophosphoethanolamine on CML

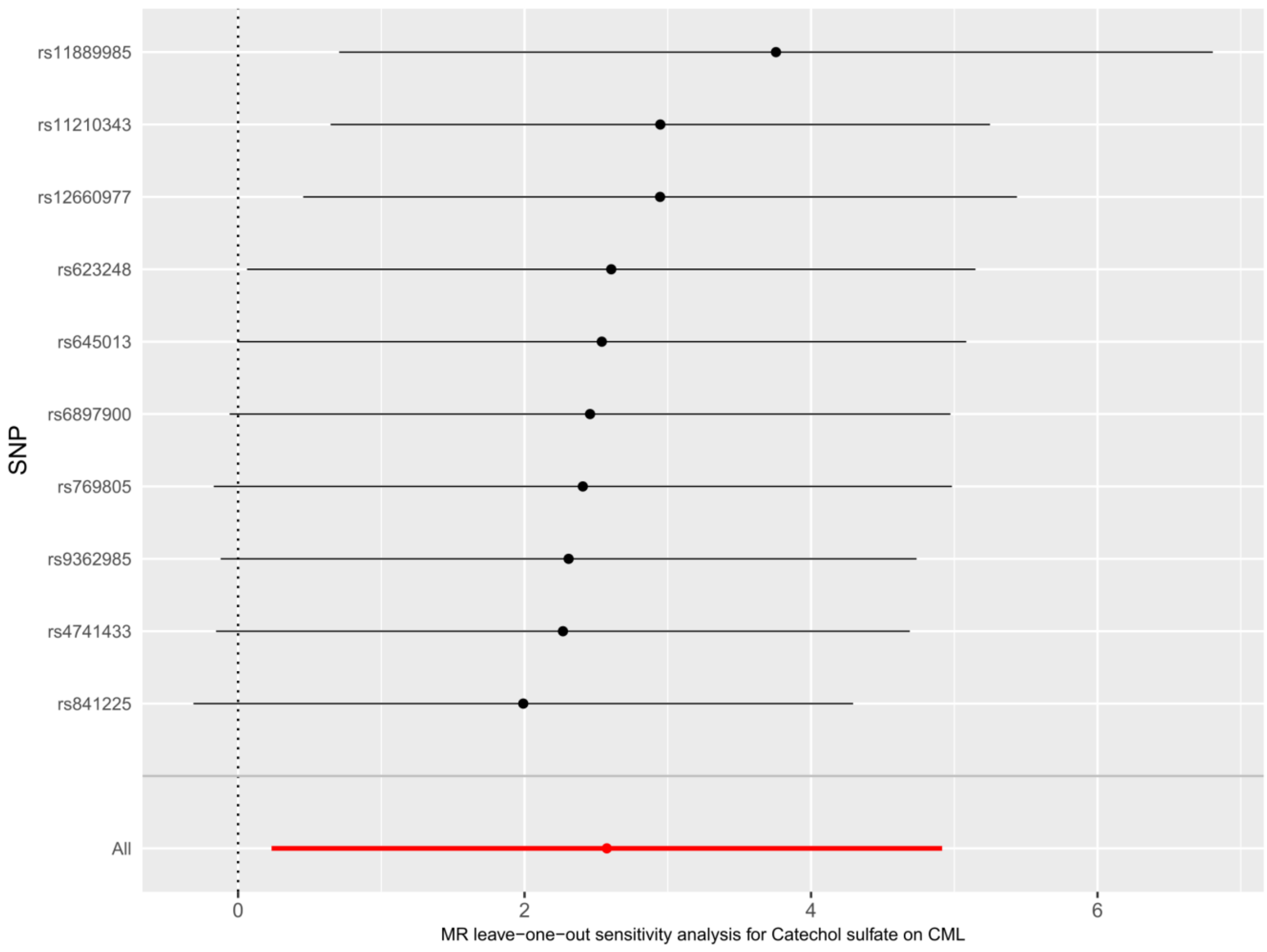
rs11889985
rs11210343
rs12660977
rs623248
rs645013
rs6897900
rs769805
rs9362985
rs4741433
rs841225
All
SNP
0
2
4
6
MR leave−one−out sensitivity analysis for Catechol sulfate on CML

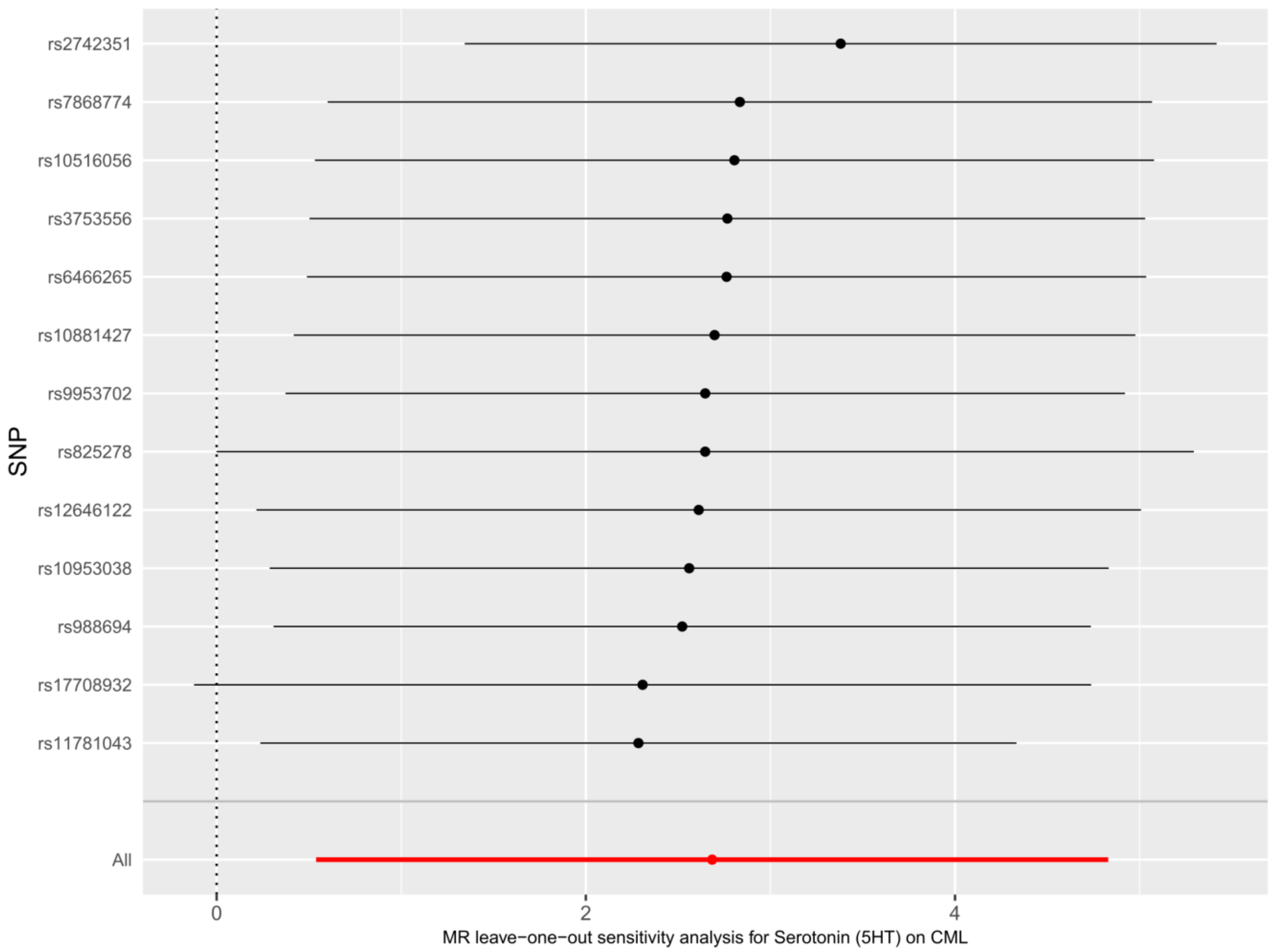
rs2742351
rs7868774
rs10516056
rs3753556
rs6466265
rs10881427
rs9953702
rs825278
rs12646122
rs10953038
rs988694
rs17708932
rs11781043
All
SNP
0
2
4
MR leave−one−out sensitivity analysis for Serotonin (5HT) on CML

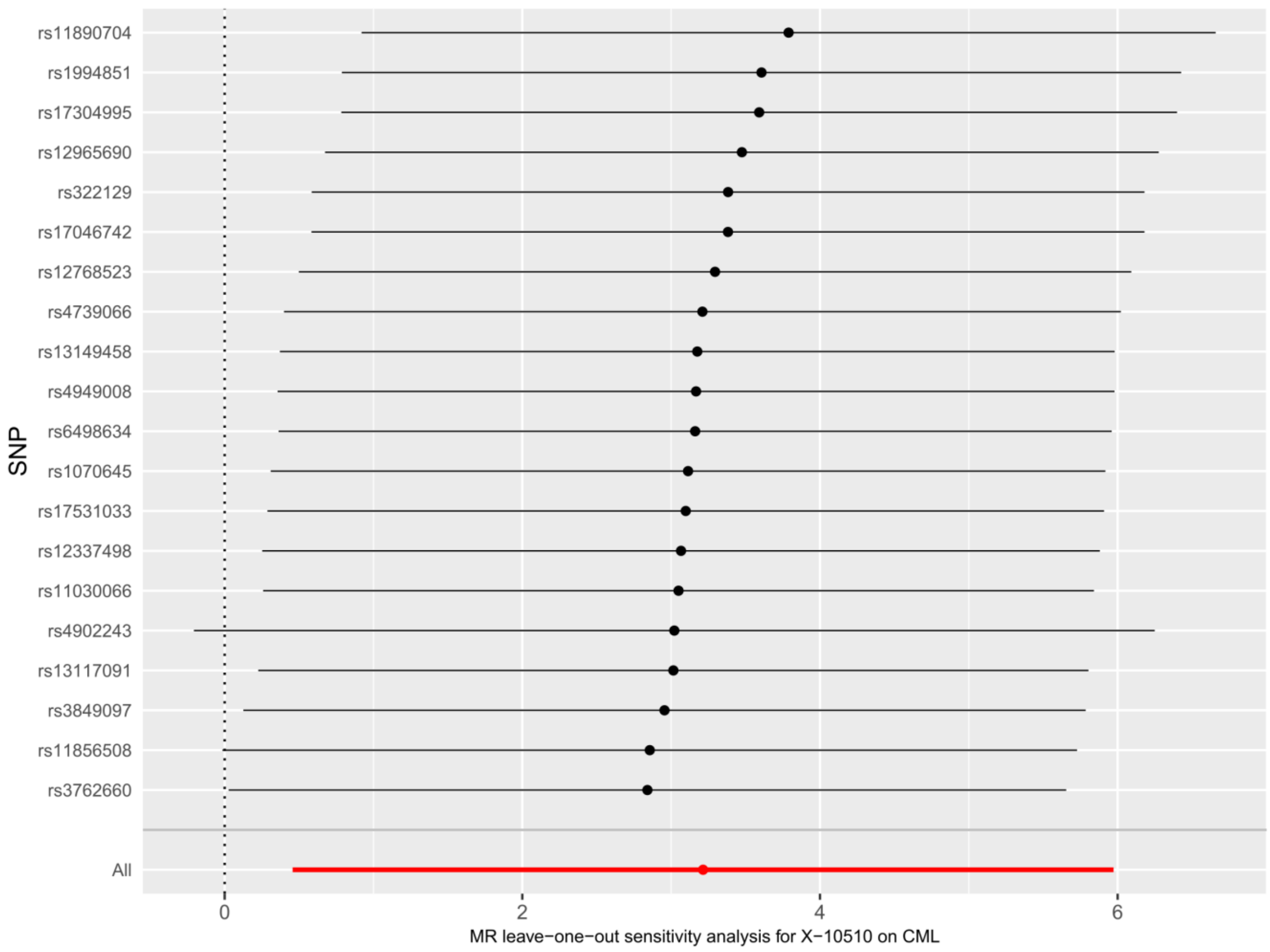

rs11890704
rs1994851
rs17304995
rs12965690
rs322129
rs17046742
rs12768523
rs4739066
rs13149458
rs4949008
rs6498634
rs1070645
rs17531033
rs12337498
rs11030066
rs4902243
rs13117091
rs3849097
rs11856508
rs3762660
All
SNP
0
2
4
6
MR leave−one−out sensitivity analysis for X−10510 on CML

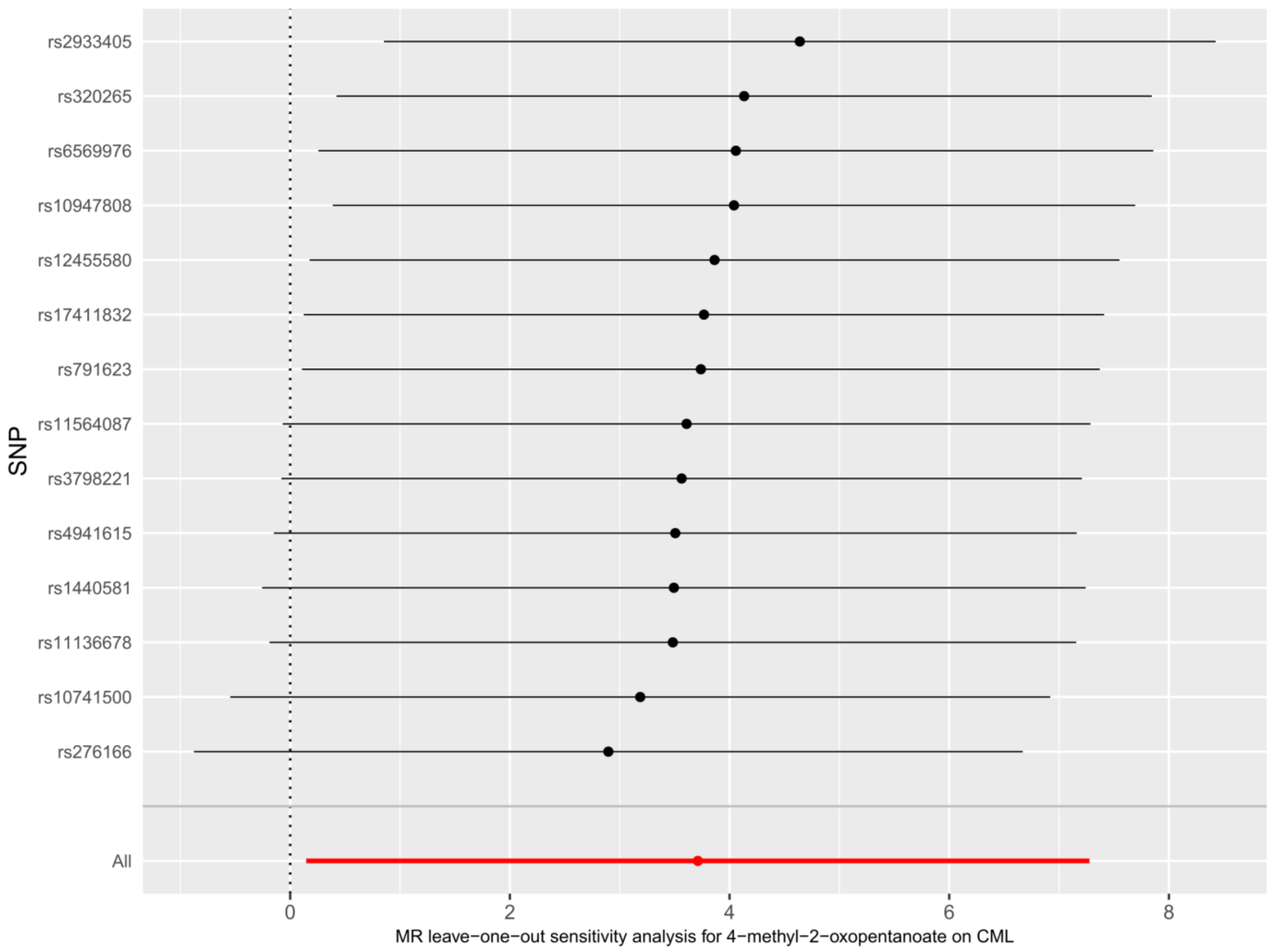
SNP
rs2933405
rs320265
rs6569976
rs10947808
rs12455580
rs17411832
rs791623
rs11564087
rs3798221
rs4941615
rs1440581
rs11136678
rs10741500
rs276166
All
0
2
4
6
8
MR leave−one−out sensitivity analysis for 4−methyl−2−oxopentanoate on CML

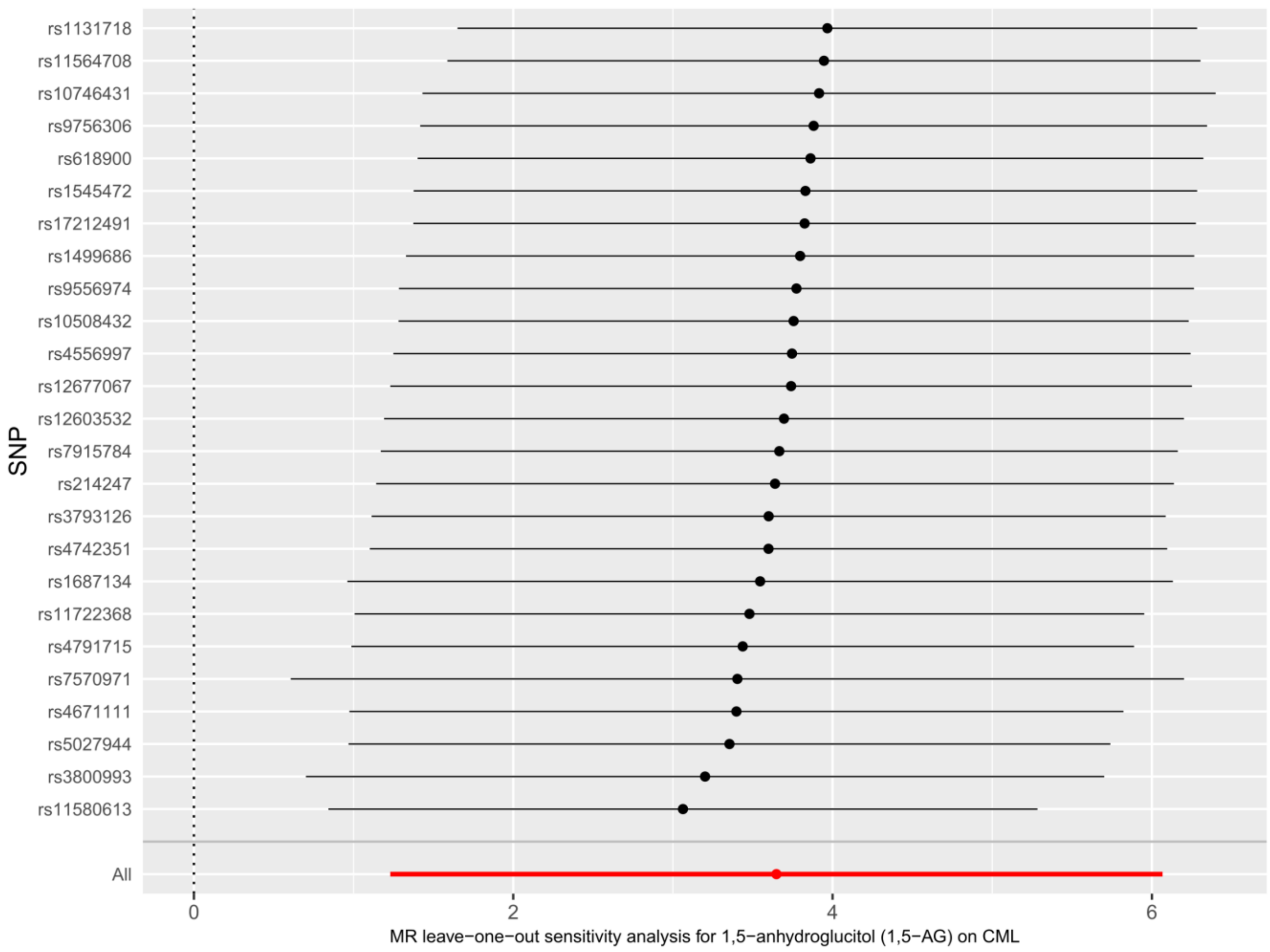

SNP
rs1131718
rs11564708
rs10746431
rs9756306
rs618900
rs1545472
rs17212491
rs1499686
rs9556974
rs10508432
rs4556997
rs12677067
rs12603532
rs7915784
rs214247
rs3793126
rs4742351
rs1687134
rs11722368
rs4791715
rs7570971
rs4671111
rs5027944
rs3800993
rs11580613
All
0
2
4
6
MR leave−one−out sensitivity analysis for 1,5−anhydroglucitol (1,5−AG) on CML

Supplementary Figure S4. GO enrichment of metabolite-associated genes linked to KEGG pathways in CML.

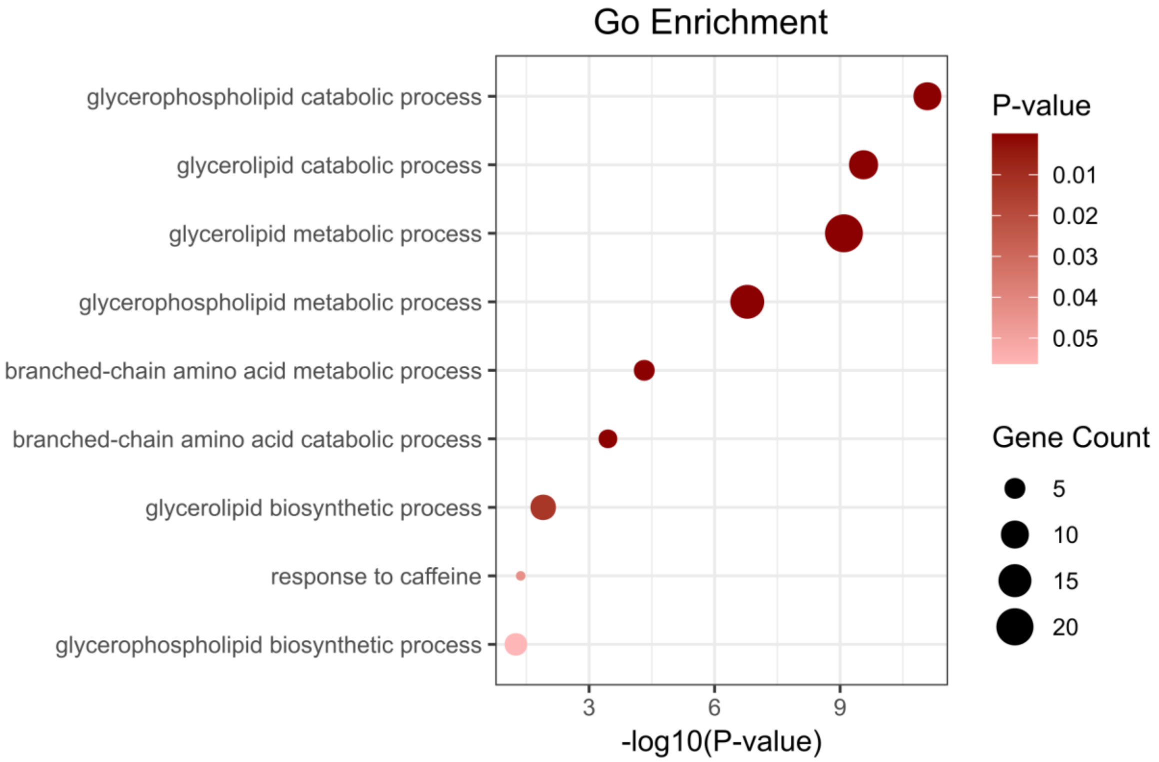

Supplement: Supplementary file 2 [file medi-104-e45217-s002.pdf]
